# Spatiotemporal organisation of residual disease in mouse and human BRCA1-deficient mammary tumours and breast cancer

**DOI:** 10.1038/s41467-026-74125-6

**Published:** 2026-06-11

**Authors:** Demeter Túrós, Morgane Decollogny, Anna Moyseos, Astrid Chanfon, Myriam Siffert, Joanne Bousmar, Lou Romanens, Jean-Christophe Tille, Olivier Tredan, Intidhar Labidi-Galy, Alberto Valdeolivas, Sven Rottenberg

**Affiliations:** 1 Institute of Animal Pathology, Vetsuisse Faculty, University of Bern, Bern, Switzerland; 2 Bern Center for Precision Medicine (BCPM), Department for BioMedical Research, University of Bern, Bern, Switzerland; 3 COMPATH, Institute of Animal Pathology, Vetsuisse Faculty, University of Bern, Bern, Switzerland; 4 Department of Medicine and Center of Translational Research in Onco-Hematology, Faculty of Medicine, University of Geneva, Swiss Cancer Center Leman, Geneva, Switzerland; 5 Division of Clinical Pathology, Department of Diagnostics, Hôpitaux Universitaires de Genève, Geneva, Switzerland; 6 Department of Medical Oncology, Centre Leon Berard, Lyon, France; 7 CRCL UMR INSERM 1052-CNRS, 5286 Lyon, France; 8 Department of Oncology, Hôpitaux Universitaires de Genève, Geneva, Switzerland; 9 Roche Pharma Research and Early Development, Roche Innovation Center Basel, F. Hoffmann-La Roche Ltd, Basel, Switzerland

**Keywords:** Breast cancer, Tumour heterogeneity, Machine learning

## Abstract

Breast cancer remains a leading cause of death worldwide. Although chemotherapy reduces primary and metastatic tumour burden, persisting drug-tolerant tumour cell populations, known as minimal residual disease (MRD), pose a significant risk of recurrence and therapy resistance. In this study, we describe the spatiotemporal organisation of therapy response and MRD in BRCA1;p53-deficient mouse mammary tumours and human clinical samples. By integrating single-cell RNA sequencing, spatial transcriptomics, and imaging mass cytometry across multiple treatment timepoints, we characterise dynamic interactions between tumour cell subpopulations and their surrounding microenvironment. Our multiomic analysis uncovers a distinct, chemotherapy-tolerant epithelial-mesenchymal transition (EMT) cancer cell population that displays a conserved expression programme in human BRCA1-deficient tumours, significantly correlates with adverse clinical outcomes, and can be pharmacologically targeted in preclinical models. We reveal the spatial distribution of residual EMT-like tumour cells within discrete anatomical niches, providing a framework for understanding the persistence of MRD and potential therapeutic vulnerabilities.

## Abstract (web summary)

The mechanisms of minimal residual disease (MRD) in breast cancer are not fully understood. Here, multi-omics on MRD in BRCA1;p53-deficient mouse mammary tumours and human samples reveal a chemotherapy tolerant epithelial-mesenchymal transition cell population that could be therapeutically targeted.

## Introduction

Breast cancer accounted for 31% of cancers in females in 2023^1^. Amongst these, triple-negative breast cancer (TNBC), the most aggressive molecular subtype with the worst prognosis, constitutes 10-15% of all breast cancer cases^2^. TNBC lacks the expression of oestrogen receptor, progesterone receptor, and human epidermal growth factor receptor-2, leading to poor response to hormone and targeted therapies^2^. Therefore, neoadjuvant chemotherapy is frequently applied as a systemic treatment option. A common combination of chemotherapeutic drugs, called TAC regimen, consists of a taxane (i.e., docetaxel), a topoisomerase II inhibitor (i.e., doxorubicin), and an alkylating agent (i.e., cyclophosphamide). To improve treatment outcomes, the regimen can be augmented with other classes of drugs, such as DNA-damaging agents and immune checkpoint inhibitors. For instance, platinum-based drugs and poly(ADP-ribose) polymerase inhibitors, both DNA-damaging agents, have been proven to be highly effective in cancers deficient in homologous recombination repair, e.g., due to *BRCA1* or *BRCA2* dysfunction. Given the overlap between *BRCA1* mutations and the TNBC phenotype^3^, the addition of such drugs has increased the pathological complete response (pCR) of non-metastatic TNBC up to 50%^4–6^. Similarly, immune checkpoint inhibitors have been shown to improve pCR^7–9^ and survival as well. Despite these advances towards personalised medicine, not all patients without distant metastasis achieve pCR and may still harbour MRD. Moreover, despite the initial chemotherapy sensitivity, patients with disseminated TNBC are rarely cured, as tumours relapse from MRD. Consequently, MRD is associated with an increased risk of recurrence and mortality^10^ and remains a major clinical challenge^11^.

MRD plays a crucial role in cancer recurrence, and the emergence of pan-resistance within residual cell populations underscores the urgent need to better understand the underlying mechanisms of drug tolerance^12–14^. MRD may comprise cancer cells in the original tumour that survive treatment by acquiring secondary drug resistance, for example due to random mutations arising in a pool of heterogeneous cancer cells before treatment and subsequently selected during therapy. Clinical observations, however, indicate that relapsing tumours may regain sensitivity to the same therapy with refractory disease only emerging after repeated dosing. Other studies similarly show that additional mutations causing refractory disease may arise during therapy, and that a non-mutational mechanism can mediate drug tolerance as a transient state preceding secondary resistance^15^. In this context, our previous studies using the maximum tolerated dose (MTD) of cisplatin in a genetically engineered mouse model for *BRCA1*-mutated breast cancer provided valuable insights into tumour response^16^. In this model, a large deletion in the *Brca1* gene prohibits the function restoration of BRCA1. Unlike PARP inhibition, which can lead to the emergence of secondary resistance, cisplatin MTD induces extensive DNA damage that BRCA1-deficient cells are unable to repair without regaining BRCA1 function, thereby preventing the development of secondary resistance mechanisms. Nevertheless, tumours persist, eventually recur, and remain responsive to subsequent cisplatin treatment^16^. This experimental setup ensures that residual tumour cells do not carry acquired resistance mechanisms, allowing us to focus specifically on transient drug tolerance as a contributor to MRD. Consistent with other studies, our previous experiments suggested that transient dormancy enables cells to evade drugs that primarily target rapidly proliferating cells^16–19^. Yet cycling residual cells have also been reported^20^, highlighting ongoing debate about the mechanisms involved. In addition, various studies have shown that drug-tolerant cells exhibit distinct transcriptional programmes driven by phenotypic plasticity, notably embryonic diapause-like signatures^21,22^, epigenetic modifications^23–25^, or metabolic shifts^26–29^. Furthermore, epithelial-mesenchymal transition (EMT) phenotypes have been shown to significantly influence poor prognosis in pan-cancer analyses^30^.

To better understand transient drug tolerance as a driver of MRD, we combine single-cell RNA sequencing (scRNA-seq), spatial transcriptomics (ST), and imaging mass cytometry (IMC) to BRCA1;p53-deficient mouse mammary tumours. These technologies allow us to resolve the spatiotemporal dynamics of several distinct tumour cell populations and their associated cellular niches, and to characterise their interplay with the tumour microenvironment. Here, we describe the functional characteristics of these molecular niches across spatial and temporal scales and directly assess their relationship with tissue-bound platinum-based chemotherapy agents through the integration of these modalities. Moreover, we identify a specific tumour cell niche and associated gene expression programme consistent with quiescence and EMT phenotypes, providing a prominent survival advantage irrespective of treatment modality. Notably, although residual tumour islands are mainly composed of the EMT niche, recurrent tumours display a composition similar to those of the primary tumours, highlighting the dynamic and reversible nature of MRD. Furthermore, we show that our findings are translatable to human BRCA1-deficient breast cancers, where the EMT niche expression programme is highly specific to residual tumour islands. Stratifying TNBC patients by EMT programme expression levels revealed a significant reduction in survival outcomes, and in vitro inhibition of key components of the EMT programme uncovers synergy with chemotherapy treatments, highlighting the clinical relevance of our findings. Beyond temporal profiling of MRD, we investigate the spatial distribution of the drug-tolerant cells and their possible interactions with the tumour microenvironment (TME). These findings unveil key components for further understanding MRD and may support the development of new therapeutic strategies aimed at eradicating residual cancer cells.

## Results

### Spatiotemporal transcriptomic profiling of MRD in the KB1P model

To chart intratumoural heterogeneity and identify key drivers of MRD, we collected primary (treatment-naïve), residual, and recurrent *K14cre*;*Brca1*^F/F^;*Trp53*^F/F^ (KB1P) mouse mammary tumours following chemotherapy. In this model, we previously demonstrated that the MTD of DNA-crosslinking therapies does not induce acquired secondary resistance, in contrast to PARP inhibitors, such as olaparib^16,31,32^. This allowed us to investigate transient mechanisms of drug tolerance in KB1P tumours, without the confounding effects of secondary resistance. Furthermore, unlike traditional xenograft models, this approach enables the investigation of residual disease in an immunocompetent context that closely mirrors human BRCA1-deficient breast cancer^33^.

**Fig. 1:**
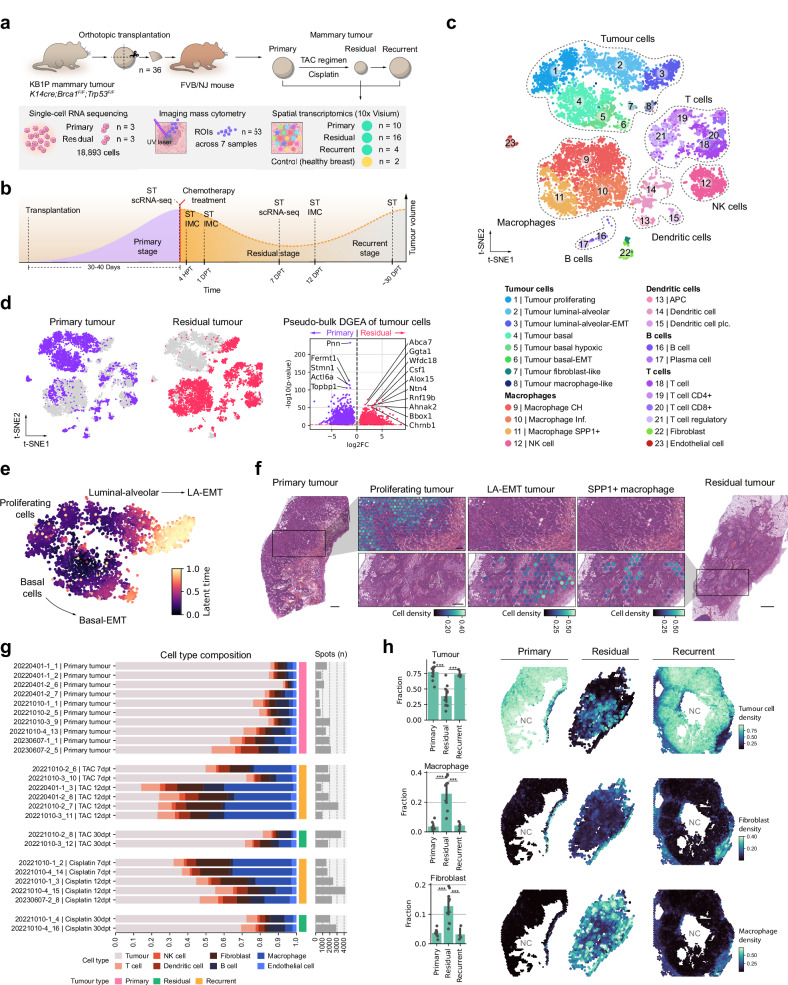
Single-cell and spatial transcriptomic profiling of MRD. **a** KB1P mouse mammary tumours were derived, subdivided, and transplanted into syngeneic FVB/NJ mice. Transplanted tumours were collected at defined stages of tumour progression (primary, residual, recurrent) and analysed by scRNA-seq, ST, and IMC. **b** After reaching the target size, transplanted tumours were either harvested as primary samples or treated with chemotherapy (TAC or cisplatin) to induce MRD. To assess early drug uptake, tumours were collected at early timepoints following TAC or cisplatin treatment (4 h post-treatment (hpt), 1-day post-treatment (dpt)) and analysed by ST and IMC. Residual tumours were sampled at 7 and 12 dpt corresponding to the period of maximal response. Recurrent tumours were sampled after regrowth to the target size. **c** T-distributed stochastic neighbour embedding (t-SNE) representation of single cells collected from primary and residual tumours, coloured by major cell types and subpopulations (*n*_Animal_ = 6*, n*_Cell_ = 11,566). **d** Cell type composition of primary and residual tumours visualised on the t-SNE plots (left panel, *n*_Primary_ = 3, *n*_Residual_ = 3*, n*_Cell_ = 11,566) and the corresponding pseudo-bulk DGEA results of tumour cells (right panel). **e** RNA velocity analysis of tumour cells indicating cell transitions from proliferating tumour cells to luminal-alveolar or basal cancer cell phenotypes (*n*_Primary_ = 3, *n*_Residual_ = 3*, n*_Cell_ = 4,347). **f** H&E-stained sections of primary and residual tumours overlaid with cell type deconvolution results of selected cell types, demonstrating distinct spatial and temporal patterning during tumour progression (scale bars: 500 µm, inset scale bars: 200 µm). Representative data are shown from a cohort of *n*_Primary_ = 10 primary and *n*_Residual_ = 9 residual tumour samples with similar results. **g** Overview of cellular composition per sample as estimated by cell type deconvolution. Bars on the right-hand side indicate the number of analysed captures spots per sample. **h** Changes in cellular fraction for selected cell types during tumour progression (left panel) and representative spatial maps (right panel, NC = necrotic core). Bar plots show the mean cell fraction across samples (black dots: individual ST samples, error bar: 95% CI, bar height: mean value). Statistical significance was assessed using a one-way ANOVA followed by Tukey’s HSD post-hoc test for pairwise comparisons (***, *p* < 0.001). Exact *p*-values for tumour cells are: Primary vs. Residual (*p* = 0.000006) and Residual vs. Recurrent (*p* = 0.000766). For macrophages: Primary vs. Residual (*p* = 0.000006) and Residual vs. Recurrent (*p* = 0.000383). For fibroblasts: Primary vs. Residual (*p* = 0.00002) and Residual vs. Recurrent (*p* = 0.00039). Sample sizes are *n*_Primary_ = 8, *n*_Residual_ = 9, and *n*_Recurrent_ = 4, where *n* values denote independent biological units (tumours, *n*_Spot_ = 45,543). Detailed sample metadata and technical replicate information are provided in Supplementary Data 13. Source data are provided as a Source Data file.

Using this model, we performed scRNA-seq on primary and residual tumours (*n* = 6), applied ST with the 10x Genomics Visium platform to primary, residual, and recurrent tumour tissue sections (*n* = 29), and conducted IMC on primary tumours immediately following chemotherapy treatment (4h-24h) as well as on residual tumours (*n* = 6) (Fig. 1a). To minimise the effects of individual genetic variability, all tumour samples were derived from two parental tumours that were subdivided and transplanted into the fourth mammary fat pad of syngeneic FVB/NJ mice. To induce MRD, animals were treated with two clinically relevant chemotherapy regimens targeting HR-deficient tumours by inducing DNA damage: cisplatin and the TAC regimen.

Tumour growth was closely monitored (Supplementary Fig. 1) and primary tumours (*n*_ST_ = 10, *n*_scRNA-seq_ = 3) were excised upon reaching 8 × 6 mm. Residual tumours were harvested at 7 days (TAC: *n*_ST_ = 2, Cisplatin: *n*_ST_ = 2) or 12 days (TAC: *n*_ST_ = 4, *n*_scRNA-seq_ = 3, Cisplatin: *n*_ST_ = 3) after treatment, corresponding to minimal tumour size (Fig. 1b). Recurrent tumours (TAC: *n*_ST_ = 2, Cisplatin: *n*_ST_ = 2) were collected once tumours again reached the target size following chemotherapy. Furthermore, tumours were sampled within the first 24 h post-treatment to assess cisplatin uptake and its relationship with tumour heterogeneity (*n*_IMC_ = 6, *n*_IMC-ROI_ = 50, *n*_ST_ = 4). This experimental design enabled the construction of a detailed spatiotemporal map of residual disease progression, capturing single-cell plasticity across multiple pivotal time points, along with spatial heterogeneity and tissue architecture.

### Single-cell dynamics of MRD

We first established the cellular composition of mammary tumours using scRNA-seq. We identified 23 distinct cell populations spanning 8 major cell types through unsupervised clustering of the integrated scRNA-seq data across tumour progression and MRD (Fig. 1c). We identified a substantial tumour cell cluster (*Krt14, Krt18*) alongside several components of the TME, including macrophages (*Cd68*), T cells (*Cd3*), B cells (*Cd19*), dendritic cells (*Lsp1, Flt3*), natural killer (NK) cells (*Ncr1, Klrb1c*), endothelial cells (*Pecam1*), and fibroblasts (*Fn1*).

Closer examination of the tumour cell cluster revealed a highly proliferative population characterised by high expression of *Mki67*, *Cdk1*, and other cell cycle-related genes, which branched into two separate lineages: basal (*Krt17*, *Krt14*, *Krt5, Acta2*) and luminal-alveolar (*Krt19*, *Krt18*) tumour cells, consistent with previous findings^34,35^ (Supplementary Fig. 2). Within the basal lineage, we identified a basal hypoxic cell group distinguished by the expression of hypoxia-responsive genes, such as *Egln1* and *Vegfa*. In both lineages, we further uncovered tumour cell populations exhibiting EMT signatures, marked by the expression of several EMT-associated genes (e.g., *Clu*, *Crip1*, *Lgals3*). In addition, two small tumour subpopulations were detected: macrophage-like tumour cells expressing genes typically associated with haematopoietic cells, mainly macrophages (*Ctss*, *Cd74*), and fibroblast-like tumour cells sharing expression signatures with fibroblasts (*Zeb2*, *Arpc1b*, *Vim*, *Mgp*).

Within the cell types of the TME, we further resolved *Cd4* + , *Cd8* + , and regulatory T cells, as well as naive, activated (*RelB* + ), and immunosuppressive (*Siglech*+ and *Ccr9* + ) dendritic cells, and plasma cells. Additionally, we identified multiple tumour-associated macrophage subtypes, including opsonin-induced M2 macrophages (*C1qa*, *C1qc*, *C1qb*), M1 macrophages, and *Spp1*+ macrophages which have previously been linked to ECM remodelling and immune evasion in other cancer types^36^.

Comparison of primary and residual cell-type compositions revealed a substantial enrichment of immune cells in the tumour microenvironment, including a significant increase in M2 and *Spp1*+ macrophages, T cells, and NK cells, as well as the EMT tumour cell populations (Fig. 1d and Supplementary Fig. 3). Differential gene expression analysis (DGEA) of tumour cells (Table 1 and Supplementary Data 1) identified a shared transcriptional programme in residual tumour cells characterised by upregulation of genes linked to lipid transport, immune modulation, macrophage polarisation, and EMT, alongside downregulation of genes associated with proliferation and cell cycle progression.

**Table 1:** Top differentially expressed genes in residual tumours

Gene	Upregulation	Gene description
Abca7	Residual	ABC transporter involved in lipid transport and mesenchymal transition^122^
Wfdc18	Residual	Marker of luminal-like states; associated with stemness^123^
Alox15	Residual	Immune-modulatory dioxygenase; suppresses immune responses^124^
Csf1	Residual	Promotes M2 macrophage polarisation; associated with poor breast cancer prognosis^125^
Ntn4	Residual	Laminin-like secreted protein associated with basement membrane interactions^126^
Rnf19b	Residual	E3 ubiquitin ligase; attenuates anti-tumour immunity^127^
Ahnak2	Residual	Regulates ERK/MAPK/WNT/MEK signalling; promotes EMT and tumour progression^128^
Bbox1	Residual	Essential for TNBC tumorigenesis^129^
Pnn	Primary	Associated with increased proliferation in cancer^130^
Fermt1	Primary	Regulates EMT and cell cycle progression^131^
Stmn1	Primary	Promotes proliferation, invasion, and anti-apoptotic signalling^132^
Actl6a	Primary	Linked to tumorigenesis across multiple cancer types^133^
Topbp1	Primary	Supports proliferation in BRCA1/2- and HR-deficient tumours^134^

RNA velocity analysis of the tumour cell clusters revealed pronounced cellular plasticity and differentiation trajectories, highlighting transitions from a proliferating tumour cell state towards luminal-alveolar and basal EMT states (Fig. 1e). These trajectories indicate dynamic reorganisation of tumour cell populations during disease progression. To examine these processes in situ, we mapped these cells within the tissue to examine their spatial organisation and the microenvironmental context in which these transitions occur.

### Spatial profiling reveals cellular heterogeneity in MRD

To characterise the spatiotemporal organisation of tumour and microenvironmental cell types and cell states in MRD, we analysed the ST data in conjunction with the corresponding histology. Cell type deconvolution informed by our single-cell reference, together with histopathological assessment, revealed that cancerous cells grow in solid nests embedded in a dense fibrous stroma (Fig. 1f).

In primary tumours, proliferating, basal, and luminal-alveolar tumour cells were broadly and evenly distributed, whereas EMT-associated tumour cell states were confined to smaller focal regions within the tumour mass. Proliferating tumour cells were densely packed, polygonal, and pleomorphic, with frequent mitotic figures, including atypical^37^ forms, indicative of high proliferative activity. A mixed immune cell infiltration is mildly present, particularly at the tumour periphery (Supplementary Fig. 4a).

In contrast, residual tumours exhibited drastic architectural remodelling. Tumour cells appeared elongated and were often difficult to delineate within a highly cellular and reactive stroma (Supplementary Fig. 4b, c), consistent with the enrichment of EMT-associated states. Mitotic figures were rare or absent, consistent with the depletion of proliferating tumour cells. Immune infiltration was predominantly mononuclear, and clusters of foamy macrophages formed discrete anatomical niches in the tumour, corresponding to regions occupied by *Spp1+* macrophages (Supplementary Fig. 4d). In the recurrent stage, however, tumour architecture and cell morphology largely resembled those of the primary tumours (Supplementary Data 2).

Across tumour stages, large necrotic areas were frequently observed, consistent with the histopathological features of *BRCA1*-mutated TNBC^33^ (Supplementary Fig. 5). Hypoxic tumour cell populations were predominantly localised in close proximity to these necrotic cores.

By analysing global changes in cell type composition across tumour-containing capture spots, we observed up to an 85% reduction in tumour cell fractions (Fig. 1g) in residual samples. This reduction was predominantly driven by the loss of proliferating, basal, and luminal-alveolar tumour cells, whereas basal and luminal-alveolar EMT phenotypes, along with the macrophage-like and fibroblast-like tumour cell types showed enrichment after treatment (Supplementary Fig. 6). In parallel, the TME underwent significant remodelling, characterised by a substantial increase in immune cells, particularly macrophages, and fibroblasts (Fig. 1h and Supplementary Fig. 7). Consistent with histopathological observations, the cellular composition of the recurrent tumours closely resembled that of the primary tumours.

Pseudo-bulk DGEA of tumour-containing capture spots between primary and residual tumours (Supplementary Data 3 and Supplementary Fig. 8) revealed enrichment of genes driving tumour-immune interactions in the residual state, including markers of innate immunity, mast cells, monocytes, and the complement system (*Cd68*, *Ms4a7*, *Fcgr1*, *Cd300a*, *Serping1*, *Cpxm1*, *Mpeg1*, *Cybb)*, and cancer proliferation-inducing genes and ECM remodelling genes, such as *Ctsk*^38^. In contrast, primary tumours showed higher expression of genes linked to cytoskeletal organisation, oxidative stress regulation, and tumour growth, including *Espn*^39^, *Egln3*^40^, *Mt1*^41^, and *Nqo1*^42^.

In summary, integration of scRNA-seq, spatial transcriptomics, and IMC enabled reconstruction of the spatiotemporal progression of residual disease in BRCA1;p53-deficient mammary tumours. This approach resolved tumour and microenvironmental cell states and identified tumour subpopulations with distinct responses to chemotherapy.

### Chemotherapy-induced changes in the transcriptional tissue landscape of MRD

To uncover the molecular changes during MRD and recurrence in an unbiased manner, we inferred cellular niches, defined as transcriptionally distinct tissue compartments, using ST. We integrated spatial gene expression profiles into a shared embedding and applied our recently published machine learning-based framework, Chrysalis^43^, to identify spatially and functionally distinct molecular tissue compartments. This approach enabled systematic analysis of tumour niche composition across space and time, as well as the corresponding gene expression programmes. In addition, it provided insight into how surviving cells are spatially organised into distinct niches that may support persistence during MRD.

**Fig. 2:**
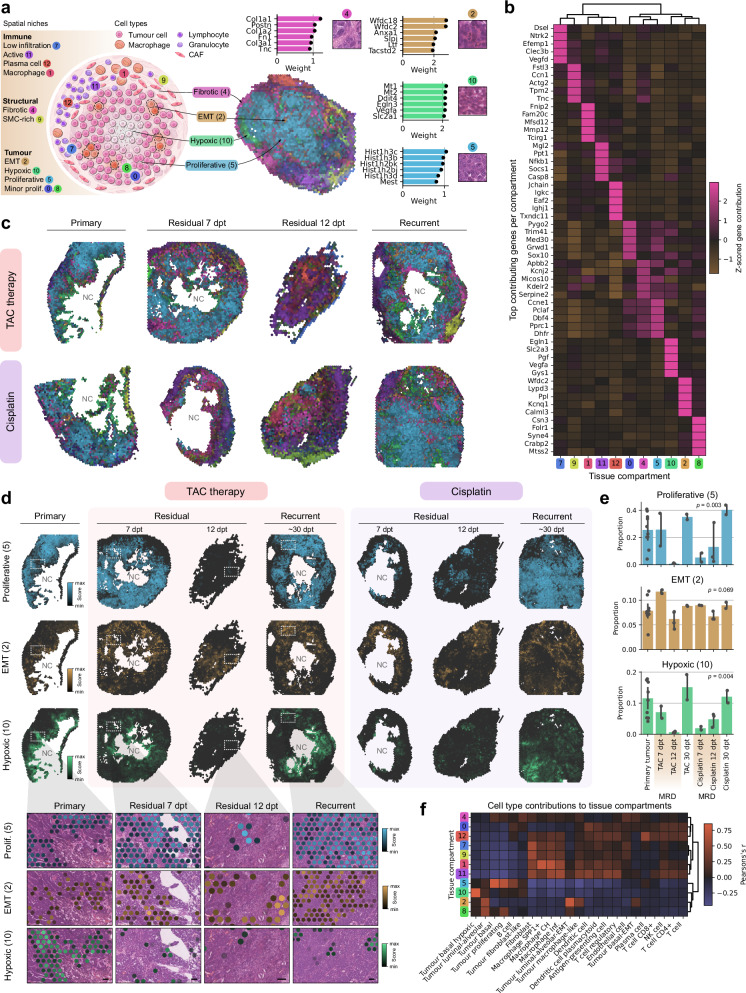
Spatiotemporal dynamics of cellular niches during tumour progression. **a** Schematic overview of the cellular niches identified by Chrysalis (left) and their MIP on a representative primary tumour tissue section (right). For niches 4, 2, 10, and 5, five genes with the highest weights from their respective gene expression programme are shown alongside representative histology image tiles (*n* = 25). **b** Heatmap showing the contribution of top-weighted genes to each identified tissue compartment (*n* = 25). **c** MIP visualisations of tissue compartments across representative tumour sections from primary, residual (7 and 12 dpt), and recurrent tumours (~30 dpt) following TAC or cisplatin treatment (NC = necrotic core). Representative data are shown from a cohort of *n* = 25 tumour samples. **d** Spatial maps of compartment scores of proliferating (5), EMT (2), and hypoxic (10) niches in representative primary, residual, and recurrent tumours after TAC or cisplatin treatment. Zoomed-in image tiles with overlaid histology highlight fine-scale spatial organisation (scale bar: 100 µm, NC = necrotic core). Representative data are shown from a cohort of *n* = 25 tumour samples. **e** Quantification of the proliferating (5), EMT (2), and hypoxic (10) niche proportions across all tumour samples (bar height: mean value, black dots: individual ST samples, error bar: 95% CI, statistical significance was assessed by one-way ANOVA: *n*_Tumour_ = 25, *n*_Spot_ = 45,543). Detailed sample metadata, including technical replicates, are provided in Supplementary Data 13). **f** Heatmap displaying correlations between cell type fractions derived from deconvolution and tissue compartment fractions across all tumour samples (*n*_Animal_ = 21, *n*_Spot_ = 45,543). Source data are provided as a Source Data file.

Characterisation of inferred tissue compartments identified five tumour cell-associated (0, 2, 5, 8, and 10) and six TME-associated (1, 4, 7, 9, 11, 12) compartments (Table 2), each with specific spatial localisation (Fig. 2a and Supplementary Fig. 9) and gene expression programmes (Fig. 2b, Supplementary Fig. 10a, and Supplementary Data 4).

**Table 2:** Spatially defined cellular niches during tumour progression and MRD

Niche ID	Category	Annotation	Description	Marker genes
5	Tumour	Proliferative tumour niche	Tumour niche occupying large regions of primary tumours - associated with high mitotic activity and tumour expansion	Histone genes, *Ncl*, *Ccne1*, *Pclaf*, *Dbf4*, *Pprc1*, *Dhfr*
10	Tumour	Hypoxic tumour niche	Niche adjacent to necrotic cores - reflects metabolic stress and hypoxia	Metallothioneins, *Egln1*, *Slc2a3*, *Pgf*, *Vegfa*, *Gys1*
2	Tumour	EMT-associated niche	Linked to cellular plasticity, migration and metastatic potential	Anxa1^135^, Wfdc family (e.g., *Slpi*)^136–139^, *Lypd3*, *Ppl*, *Kcnq1*, *Calml3*^140^, *Csn3*
0	Tumour	Minor proliferative niche	Subset of proliferative niche present in selected samples - overlaps with proliferative programmes	Proliferation-associated genes
8	Tumour	Minor proliferative niche	Proliferative niche selectively eliminated by chemotherapy	Proliferation-associated genes
4	TME	Fibrotic stroma	Proximal to tumour cells - ECM-rich reactive stroma	Collagens, *Tnc*, *Postn*, *Fn1*
9	TME	Smooth muscle-like stromal niche	Enriched for contractile and structural programmes	Collagens, smooth muscle-associated genes
7	TME	Low immune infiltration niche	Mild immune presence with complement and macrophage-associated features	*C4b*, *Retnla*
1	TME	Monocytic / macrophage niche	Dominated by myeloid cells	*Cd68*, cathepsins
11	TME	Activated immune niche	Lymphocyte infiltration and immune activation	*Retnla*, *Cd74*, *C3*, *Ccl8*
12	TME	Plasma cell / humoral immune niche	Co-localises with plasmacytic cells - active antibody production	*Igkc*, *Jchain*, *Ighj1*, immunoglobulin genes

Among the tumour-associated niches, a proliferative compartment (5) occupied large regions of the primary tumour mass and was characterised by high cell cycle activity, consistent with rapid tumour growth. A hypoxic tumour niche (10) was spatially associated with necrotic cores, reflecting metabolic stress and limited oxygen availability. In addition, an EMT-associated niche (2) displayed features linked to increased cellular plasticity and migratory potential, suggesting a role in tumour invasion and persistence following treatment. Two additional tumour-associated compartments were less prevalent, appearing only in subsets of samples (0), including one niche that was selectively lost following chemotherapy (8).

The TME was organised into multiple distinct niches spanning fibrotic stroma and increasing levels of immune infiltration. Fibrotic stromal compartment (4) proximal to tumour cells was enriched for extracellular matrix components, indicative of a fibrotic and reactive stroma. Smooth muscle-enriched stromal compartment was characterised by contractile and structural expression programmes consistent with smooth muscle cells (9). Progressively more immune-enriched niches (7, 11, 12) included regions with complement-associated programmes, followed by compartments dominated by activated immune cells and lymphocytes. The most immune-rich compartment showed strong signatures of B cell activation and humoral immune responses and spatially coincided with plasmacytic cell aggregates observed in histological sections. Alongside these niches, a macrophage-rich compartment (1) was dominated by myeloid gene expression, including macrophage and monocyte markers.

To investigate the temporal evolution of the uncovered cellular niches during tumour growth, MRD, and relapse, we analysed four different time points - primary tumour, MRD 7 dpt, MRD 12 dpt, recurrent tumour (~30 dpt) - in both the TAC and the cisplatin-treated samples. Maximum intensity projection-based (MIP) visualisation of compartment distributions revealed substantial changes in tissue composition during MRD (Fig. 2c and Supplementary Fig. 10b). Primary tumours were predominantly composed of the proliferating (5) tumour niche, surrounded by fibrotic stroma (4) and immune-associated compartments. Surrounding the necrotic cores, the hypoxic tumour niche (10) was dominant, whereas the EMT-associated niche was distributed throughout the tumour without a discernible spatial preference.

In the residual tumours, we observed extensive remodelling, with the majority of tumour tissue replaced by immune-associated compartments (11, 12), indicating significant immune cell infiltration and phagocytic activity. In contrast, recurrent tumours displayed a tissue composition closely resembling that observed before chemotherapy.

Next, we focused on characterising the temporal dynamics of the proliferating (5), hypoxic (10) and EMT-associated (2) niches (Fig. 2d). Both proliferating and hypoxic tumour cell niches diminished significantly after chemotherapy in both treatment groups, whereas the EMT (2) compartment remained largely unchanged. Quantification of tumour niche coverage confirmed the near-complete depletion of the proliferating and hypoxic compartments in MRD (Fig. 2e). Yet no significant change for the EMT compartment was detected, indicating drug-tolerance and stability of this tumour niche and its associated cell types. Treatment-specific changes in remodelling kinetics were also observed. In the TAC-treated samples the maximal remodelling was detected at 12 dpt, whereas in the cisplatin-treated tumours it reached maximal effect at 7 dpt. Similar trends were observed in the TME-associated niches (Supplementary Fig. 11-12). Amongst these, the plasma cell-associated cellular niche was enriched specifically in the TAC-treated MRD samples.

To link tissue compartments to cellular identities we correlated niche composition with the cell type deconvolution results (Fig. 2f). Tumour niches showed strong correlation with the identified tumour cell types: the proliferating (5) niche correlated with proliferating and basal tumour cells, while the hypoxic tumour cells were exclusively mapped to the hypoxic compartment (10). The EMT niche was strongly correlated with the luminal-alveolar-EMT cell population. Immune cell types corresponded to the TME niches with substantial enrichment in macrophages in tissue compartments 1 and 11, and plasma cells in compartment 12. Some compartments could not be fully assigned to specific cell types, reflecting the complexity of the identified niches and cell states not fully represented in the single-cell reference.

Together, these results reveal dynamic yet reversible reorganisation and interplay between distinct cellular niches in MRD, while highlighting the persistence of a stable, chemotherapy-tolerant EMT-associated tumour niche.

### Functional characterisation of MRD

**Fig. 3:**
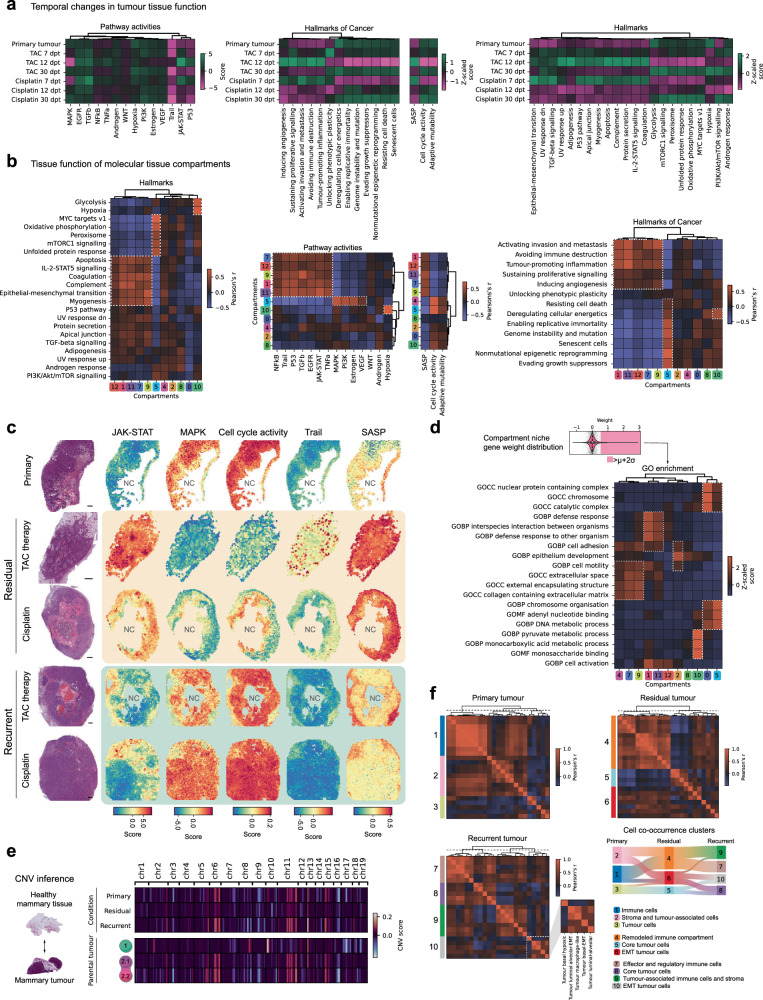
Functional dissection of tumour dynamics across spatial and temporal scales. **a** Heatmaps illustrating changes in intracellular pathway activities, significantly enriched Hallmark pathways, Hallmarks of Cancer gene sets, and additional gene set scores across tumour progression from primary tumours to MRD and recurrence (*n* = 25, *n*_Spot_ = 45,543). **b** Heatmaps showing correlations between cellular niches and intracellular pathway activities, significantly enriched Hallmark pathways, and Hallmarks of Cancer gene sets. Terms discussed in the main text are highlighted with white dashed rectangles (*n* = 25, *n*_Spot_ = 45,543). **c** Spatial maps of selected gene set and pathway activity scores in representative samples, illustrating distinct spatiotemporal organisation across tumour stages, alongside H&E staining (scale bar: 500 µm). Representative data are shown from a cohort of *n* = 21 tumour samples. **d** GO enrichment heatmap of gene expression programmes associated with each cellular niche. Genes were selected based on high contribution to each niche (μ + 2σ threshold). Terms highlighted in the main text are indicated by white dashed rectangles (*n* = 25, *n*_Spot_ = 45,543). **e** CNV inference based on contrasting mammary tumour samples with healthy mammary tissue samples. Heatmaps display chromosomal alterations ordered by tumour stage and parental tumour origin, revealing strong correlation with parental line–specific CNV signatures (*n* = 25, *n*_Spot_ = 45,543). **f** Heatmaps showing cell colocalisation clusters determined by hierarchical clustering with a dendrogram distance cutoff of 3.5 (dashed line). Sankey diagram illustrates the reorganisation of spatial clusters across tumour progression (*n* = 25, *n*_Spot_ = 45,543). Source data are provided as a Source Data file.

We next sought to characterise the spatiotemporal changes in functional treatment response and how the identified cellular niches can contribute to these dynamics. To this end, we inferred pathway activity scores for cancer-relevant signalling pathways using PROGENy^44^ (Fig. 3a, and Supplementary Fig. 13). In primary tumours, we observed strong downregulation of the Trail apoptotic pathway, which partially recovered at the time of maximal treatment response but was further reduced in recurrent tumours. When inhibited, the Trail apoptotic pathway has been implicated in tumour initiation and progression^45^. Similar temporal trends were detected for p53 and JAK-STAT pathways, while the activity of EGFR and TGF-β pathways was increased. While JAK-STAT activation has been linked to early cancer progression, in our data it was predominantly induced during MRD^46^. In contrast, the MAPK signalling pathway was significantly downregulated during MRD, consistent with the diminishing proliferating tumour cell population.

Correlation analysis between pathway activity and cellular niches revealed that TME-associated compartments were strongly correlated with increased NFkB, Trail, p53, TGF-β, EGFR, JAK-STAT, and TNFα signalling (Fig. 3b). The proliferating tumour niche (5) specifically correlated with MAPK, PI3K, and oestrogen signalling, while the hypoxic niche (10) aligned with increased hypoxia pathway activity. Investigation of the spatial pathway activity maps further supported the TME-associated JAK-STAT pathway enrichment and the association of MAPK activation and Trail inhibition with the proliferating tumour mass (Fig. 3c).

We extended this analysis using the Hallmarks of Cancer gene sets from a recent study^47^, identifying cancer hallmarks activated during MRD, including *tumour-promoting inflammation* and *evasion of immune destruction* (Fig. 3a and Supplementary Fig. 14). These cancer hallmarks were strongly correlated with TME-related compartments (Fig. 3b). In contrast, the proliferating tumour niche exhibited enrichment for hallmarks associated with *evading growth suppressors* and *enabling replicative immortality*, while the hypoxic niche correlated with *deregulating cellular energetics*. Consistent with niche remodelling during treatment, these tumour-associated programmes were markedly reduced during MRD as the corresponding tumour cell populations diminished in response to chemotherapy.

Since drug-tolerant residual cells have been reported to exhibit a senescence-like phenotype^48,49^, we sought to investigate the senescence-associated secretory phenotype (SASP) across MRD and the identified cellular niches. We observed significant upregulation of the SASP signature^50^ during MRD compared with both primary and recurrent tumour stages (Fig. 3a and Supplementary Fig. 15). Correlation of SASP activity score with tissue compartments revealed a strong anti-correlation with the proliferating tumour niche and strong positive correlations with the TME-associated immune cell compartments. However, no significant correlation was detected with either the EMT or hypoxic tumour niches (Fig. 3b). At the capture spot level (Fig. 3c), elevated SASP activity was predominantly localised to the TME, in particular to fibrotic structures surrounding the tumour mass. These data suggest a shift from proliferative signalling towards a SASP and immune-modulatory pathways, driven largely by the remodelling of the tumour microenvironment

Given that quiescence and cell cycle alterations are known features of drug-tolerant tumour states^16,51^, we next quantified the cell cycle activity using phase-specific marker gene expression. Our analysis revealed a significant reduction of cell cycle activity in residual tumours at 12 days after TAC treatment and 7 days after cisplatin treatment (Fig. 3a and Supplementary Fig. 15), followed by recovery in recurrent tumours. Cell cycle activity strongly correlated with the proliferating tumour niche and diminished only at time points corresponding to maximal treatment response, while showing moderate correlations with the hypoxic niche and moderate anticorrelation with TME-associated compartments (Fig. 3b). These trends were further corroborated by the spatial distribution of cell cycle activity scores (Fig. 3c).

Finally, we assessed the adaptive mutability signature^52^ (Fig. 3a, b and Supplementary Fig. 15), which reflects reduced expression of DNA repair factors, thereby enhancing the possibility of gaining additional mutations and secondary resistance. This signature was downregulated during MRD, consistent with the decrease of proliferating tumour cell abundance at this stage.

Continuing the functional characterisation, we assessed the enrichment of 50 gene sets from the Hallmarks MsigDB collection^53^ using overrepresentation analysis (ORA). Significantly enriched Hallmarks (adjusted *p* < 0.05) were first examined along the temporal axis (Fig. 3a and Supplementary Fig. 16, 17). Hallmarks that were downregulated at time points corresponding to maximal treatment responses included *MYC target genes*, *mTORC1 signalling*, *oxidative phosphorylation*, *unfolded protein response*, *peroxisome*, and *glycolysis*. Similarly, *hypoxia*, *PI3K/Akt/mTOR signalling*, and *androgen response* Hallmarks were depleted in the residual tumours but notably, their lowest enrichment values corresponded to 12 dpt in cisplatin-treated residual tumours, indicating delayed response of these expression programmes relative to TAC treatment. Amongst these, *glycolysis* and *hypoxia* signatures were strongly associated with the hypoxic (10) compartment, whereas *MYC target genes*, *oxidative phosphorylation*, *unfolded protein response*, *peroxisome*, and *mTORC1 signalling* was correlated with the proliferating (5) tumour niche (Fig. 3b). Conversely, Hallmarks associated with *epithelial-mesenchymal transition*, *complement*, and *IL-2-STAT5 signalling* were activated during MRD (Fig. 3a), consistent with immune activation and microenvironmental remodelling during this stage.

Building on these analyses, we acquired compartment-specific gene signatures (Supplementary Data 5) by selecting the top-weighted genes (mean + 2 SD) and performed ORA using Gene Ontology (GO)^54^ gene sets (Fig. 3d). The proliferating tumour niche (5) was enriched for terms related to cellular proliferation and DNA replication, whereas the hypoxic niche (10) showed enrichment for pathways related to altered metabolic processes. The EMT niche (2) was characterised by *cell motility* and *cell adhesion* terms. TME niches rich in fibroblasts and connective tissue (4, 7, 9) displayed expression programmes related to extracellular structure maintenance, while immune-rich compartments (1, 11) were associated with defence response terms.

To assess whether genetic variability contributes to the observed niche diversity, we performed copy number variation (CNV) inference, followed by unsupervised clustering based on CNV signatures (Fig. 3e). Consistent with the notion that transient transcriptional signatures drive drug tolerance rather than stable genetic alterations, CNV-based clusters did not align with specific cell types or tissue compartments (Supplementary Fig. 18). Instead, CNV signatures differed significantly between tumours of origin. Tumours derived from parental tumour 1 were characterised by chromosomal amplifications on chromosome 2, 8, 10, 13, and deletions on chromosome 17. Conversely, samples originating from parental tumour 2 (2.1, 2.2) showed amplified chromosomal regions on chromosomes 2, 5, 6, 11, 13, and 15, and deletions on chromosome 16. Furthermore, these CNV signatures were consistent with those inferred from the scRNA-seq data (Supplementary Fig. 19).

Given the differential treatment responses of tumour niches, with the EMT compartment remaining largely unaffected and the proliferating tumour niche undergoing pronounced depletion during MRD, we conducted detailed molecular profiling of these specific niches by performing pseudo-bulk DGEA on the subset of capture spots with high (>0.80) compartment scores exhibiting dominant niche signatures (Supplementary Fig. 20a). Transcription factor activity analysis revealed increased activity of *E2F1-4* and *Myc* in the proliferating tumour niche as opposed to the increased activity of *Smad3*, *Jun*, *Sp1*, and *Hif1a* in the EMT niche (Supplementary Fig. 20b). Differentially expressed genes in the proliferating tumour niche were linked to MAPK, oestrogen, and PI3K signalling pathways. In contrast, genes upregulated in the EMT tumour niche were linked to p53, JAK-STAT, and hypoxia pathways (Supplementary Fig. 20c). Hallmark pathway analysis further highlighted distinct functional programmes between the niches. The EMT tumour niche showed differentially expressed genes involved in epithelial-mesenchymal transition, TNFα signalling, interferon alpha/gamma response pathways, and hypoxia, whereas, the proliferating tumour niche exhibited strong enrichment of G2M checkpoint, *E2f* and *Myc* target gene signatures (Supplementary Fig. 20d, e).

To complete our spatiotemporal characterisation, we analysed the colocalisation of identified cell types across primary, residual, and recurrent tumour stages to capture dynamic reorganisation and compartmentalisation (Fig. 3f and Supplementary Data 6). In primary tumours, three distinct clusters were identified, corresponding to immune cells of the TME (1), stromal cells (2), and tumour cells (3). During MRD, stromal cells increasingly colocalised with lymphocytes and macrophages, forming a remodelled immune-stromal cluster (4). Residual tumour cells predominantly organised into an EMT-associated niche, with tumour cells showing minimal spatial association with other cells (6) and a small fraction of remaining core tumour cells (5). In recurrent tumours, immune clusters (7) became more segregated from infiltrated stroma (9), while the EMT cluster (10) remained distinct from the bulk tumour cell cluster (8). Overall, the spatial organisation observed in recurrent tumours closely resembled that of the primary tumours. Furthermore, moderate anticorrelation was observed between the TME immune cells and the tumour cells, suggesting limited tumour infiltration, especially during MRD.

To characterise cell-cell communication networks within the identified cellular niches, we performed ligand-receptor interaction analysis using LIANA + ^55^, identifying highly correlated signalling axes for each niche (Supplementary Fig. 21). The proliferative niche (5) was characterised by collagen-*Ddr1* and *Igf1*-integrin signalling, emphasising a growth-promoting environment^56,57^. Conversely, the EMT-associated niche (2) utilised *Wnt7b*-*Fzd1*/*Lrp6* pathways, facilitating cellular plasticity and migration^58^. Furthermore, *Calml3*-*Kcnq1* interaction was found to be highly associated with this niche, revealing the role of the potassium channel *Kcnq1*, which has been implicated in disrupting epithelial homeostasis^59^. In contrast, the hypoxic niche (10) exhibited high enrichment for the *Vegfa*-*Cd44* axis, suggesting a coordinated programme for metabolic adaptation and survival under stress^60^.

Within the TME, we observed collagen VI interactions in the fibrotic compartment (4), and *Itga5*-mediated interactions in the smooth muscle-like niche (9), which plays an important role in modulating antitumour immunity^61^. Immune compartments exhibited distinct signalling: the macrophage-rich compartment (1) was defined by *Trem2* and *Apoe*-mediated interactions, indicative of immunosuppressive myeloid activity^62^, while the activated immune niche (11) showed high enrichment for complement signalling. These results suggest that these ligand-receptor interactions coordinate metabolic stress, ECM remodelling, and immune evasion in a spatially organised manner.

In summary, the functional characterisation of MRD highlights dynamic tumour reorganisation marked by the downregulated apoptotic and proliferative pathways, persistence of EMT and immune-associated niches, and emergence of a senescence-like phenotype in drug-tolerant cells. These findings underscore the role of tumour plasticity and microenvironment interactions in therapy resistance and tumour recurrence.

### Multimodal characterisation of cellular cisplatin uptake in mammary tumours

**Fig. 4:**
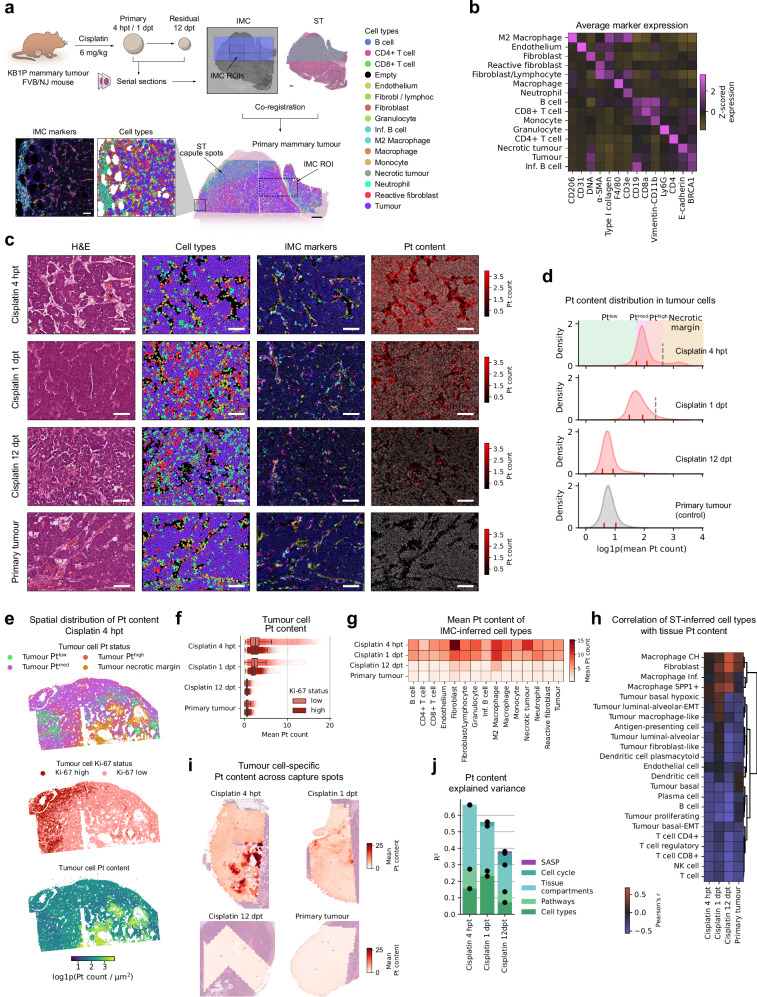
Spatial and transcriptional determinants of cisplatin uptake and distribution in mammary tumours. **a** Serial sections of cisplatin-treated mammary tumours were subjected to IMC (*n*_Animal_ = 6, *n*_ROI_ = 50) and ST (*n*_Animal_ = 4), followed by co-registration (*n*_Pair_ = 4) based on tissue morphology to align the two modalities. IMC-based single-cell annotations were coupled with transcriptional information (*n*_Animal_ = 6, *n*_ROI_ = 50, scale bars: 500 µm, inset scale bar: 50 µm). **b** Heatmap showing the relative expression of metal-conjugated antibodies used for cell type classification (*n*_Animal_ = 6, *n*_ROI_ = 50). **c** Representative IMC acquisitions of 6 mg/kg cisplatin-treated tumours collected at 4 hpt, 1 dpt, and 12 dpt, along with an untreated tumour. Shown are corresponding histological images, segmented single cells, MIP of six IMC channels (light blue: CD206, yellow: CD3, green: CD3e, brown: F40/80, dark blue: DNA, pink: αSMA), and the total Pt signal summed across detected Pt isotopes are shown (scale bar: 75 μm). Images are shown from a cohort of *n*_Animal_ = 6 with consistent phenotypes observed across all scanned regions. **d** KDE plots showing the distribution of normalised Pt counts per μm² in tumour cells across conditions. Vertical red lines represent the 15th and the 85th percentiles, and the dotted line indicates the GMM cutoff. These thresholds define Pt^low^, Pt^mod^, Pt^high^, and necrotic margin tumour cell populations (*n*_Animal_ = 6, *n*_ROI_ = 50). **e** Spatial map of a representative tissue section showing tumour cells classified by Pt and Ki-67 status as well as the measured intracellular Pt content at 4hpt. **f** Pt signal (counts / μm^2^) in Ki-67^+^ proliferative and Ki-67^-^ non-proliferative tumour cells across conditions (centre line: median, box limits: upper and lower quartiles, whiskers: 1.5x interquartile range, salmon-pink dots: individual cells; *n*_Animal_ = 6, *n*_ROI_ = 50; detailed sample metadata are provided in Supplementary Data 13). **g** Heatmap of mean intracellular Pt signal (counts / μm^2^) across cell types and conditions (*n*_Animal_ = 6, *n*_ROI_ = 50). **h** Heatmap showing the correlation between tumour cell Pt content mapped to capture spots and the deconvolved cell type proportions from ST data (*n*_Animal_ = 1 per condition). **i** Spatial maps of average tumour cell Pt content across capture spots at different time points (*n*_Animal_ = 1 per condition). **j** Coefficient of determination (*R*^2^) of spatially informed random forest models trained using MISTy to infer tumour Pt content. Contributions of individual transcriptional feature sets are indicated by stacked bar plots for individual biological subjects (*n*_Animal_ = 1 per condition; model training performed on individual spatial spots per sample). Source data are provided as a Source Data file.

To investigate spatial heterogeneity in chemotherapy uptake at single-cell resolution, we combined IMC and ST data from serial tumour sections (Fig. 4a). Co-registration of IMC (*n*_IMC-ROI_ = 50 across *n*_4 hpt_ = 2, *n*_1 dpt_ = 2, *n*_12 dpt_ = 1, *n*_Primary (non-treated)_ = 1 animals) and Visium sections (*n*_4 hpt_ = 1, *n*_1 dpt_ = 1, *n*_12 dpt_ = 1, *n*_Primary (non-treated)_ = 1) based on H&E staining and IMC bright-field images allowed transcriptomic features to be mapped onto individual cell populations. Using this integrated framework, we analysed tumours treated with 6 mg/kg cisplatin and harvested at 4 h, 1 day, and 12 days post-treatment, focusing on platinum (Pt) accumulation measured by IMC. Tissue sections were stained with a custom panel of metal-conjugated antibodies (Supplementary Data 7), and based on these markers, 15 distinct cell types were identified across tumour and microenvironment compartments, enabling direct assessment of cell type–specific cisplatin uptake and its potential contribution to the cellular and niche-level shifts observed during MRD. IMC data were processed using deep learning–based cell segmentation followed by cell phenotyping via Pixie pixel clustering^63^, enabling classification of tumour, immune, and stromal populations (Fig. 4a, b and Supplementary Fig. 22).

First, we quantified intracellular Pt signal intensity to infer cisplatin uptake within tumour tissue. As Pt content was directly measured by IMC detectors without signal amplification, we first evaluated the isotopic distribution and kinetics of Pt within the tissue. The measured abundances of ^194^Pt, ^195^Pt, and ^196^Pt closely matched the expected isotopic distribution (^190^Pt: 0.01%, ^192^Pt: 0.78%, ^194^Pt: 32.9%, ^195^Pt: 33.8%,^196^Pt: 25.2%,^198^Pt: 7.36%)^64^, and exhibited the anticipated clearance kinetics following treatment. In contrast, ^198^Pt showed higher intensity than expected and lacked spatial correlation with the other Pt isotopes, indicating nonspecific signal, leading us to exclude it from further analysis (Supplementary Fig. 23). Signals for ^190^Pt, and ^191^Pt were indistinguishable from untreated controls, suggesting that isotope levels below the detection limit, and were similarly omitted from the analysis.

Overall, IMC revealed high extratumoural Pt concentrations and a relatively even intracellular Pt distribution (Fig. 4c). Pt concentration peaked at 4 h post-treatment (hpt) and remained high at 1 dpt, followed by a pronounced decrease in residual tumours, where most cells lacked detectable Pt signal, resembling the untreated sample. This indicates efficient removal of Pt adducts induced by cisplatin treatment^65^ (Supplementary Fig. 24a, b). To further stratify tumour cells based on Pt burden, we classified cells into Pt^low^ (below the 15th percentile), Pt^mod^ (between the 15th and 85th percentile), and Pt^high^ (above the 85th percentile) groups based on intracellular Pt content. We introduced a Gaussian mixture model-based (GMM) thresholding to identify cells with prominent Pt levels at 4 hpt and 1 dpt, as visualised by kernel density estimate (KDE) plots (Fig. 4d). Spatial mapping revealed that tumour cells with very high Pt accumulation resided in perinecrotic regions, consequently, we labelled this population as necrotic margin tumour cells (Fig. 4e and Supplementary Fig. 24c).

Next, we classified tumour cells by Ki-67 status to examine whether active cell cycling influences cisplatin uptake (Fig. 4f). We found minimal variation in mean intracellular Pt content in cycling and non-cycling cells, suggesting largely uniform drug distribution irrespective of cell cycle status. However, at 4 hpt, a subset of non-cycling (Ki-67^low^) tumour cells exhibited markedly elevated Pt levels. Spatial mapping revealed that these cells localised to the necrotic tumour margin, a region characterised by the absence of cycling tumour cells. Of note, significant Pt accumulation was also observed within necrotic tissue as early as 4 hpt, preceding cisplatin-induced cell death^16^. Together these results suggest that local tissue context, such as the presence of fibroblasts, immune cells and necrotic tissue, might influence Pt distribution within the tumour.

We followed the analysis by examining Pt accumulation across cell types of the TME. The highest Pt concentrations were detected in fibroblasts and M2 macrophages (Fig. 4g), consistent with previous findings reporting extensive cisplatin binding to collagen-rich stroma^66^. Notably, the M2 macrophages retained elevated Pt levels in residual tumours, which may contribute to the attenuation of immunosuppressive microenvironment following cisplatin treatment^67^. Neighbour enrichment analysis revealed dense spatial co-localisation of M2 macrophages and fibroblasts across tumour stages, with the exception of residual tumours (12 dpt). This is also evident upon visual inspection of the fibrotic capsule surrounding the tumour cells. Pt^mod^ tumour cells, which constitute the majority of the tumour mass, exhibited negative neighbour enrichment with most other cell types across all conditions, indicating spatial segregation (Supplementary Fig. 25).

To explore whether transcriptional features influence drug distribution, we analysed the co-registered ST data by mapping the Pt intensity from IMC measurements to spatial capture spots. We first assessed correlations of intracellular Pt content with the inferred cell type abundances (Fig. 4h-i). Fibroblasts, *Spp1*+ macrophages and CH macrophages showed moderate correlations with Pt content. In contrast, transcriptionally defined tumour cell states only showed weak correlation with Pt, with the highest Pearson’s r of 0.25 at 1 dpt exhibited by hypoxic tumour cells, followed by luminal-alveolar-EMT tumour cells. Weak anticorrelations were also found at 4 hpt and 1 dpt for lymphocytic immune populations and proliferating tumour cells.

To further investigate transcriptional features that might influence drug distribution specifically within tumour cells, we applied MISTy^68^, an explainable machine learning framework, to model Pt concentration measured in the tumour cells using spatially-informed relationships derived from selected transcriptional features (Fig. 4j). This feature set included tissue compartments predicted with Chrysalis (Supplementary Fig. 26 and Supplementary Data 8), along with inferred cell type proportions, pathway activities, SASP, and cell cycle scores. The trained decision tree models explained 66% of the variance in tumour cell Pt content at 4 hpt. This power decreased steadily over time (1 dpt: *R*^2^ = 0.56, 12 dpt: *R*^2^ = 0.38). Most of the explained variance was attributed to the tissue compartments followed by inferred cell type proportions. Analysing the feature importances revealed that the strongest contributors were tissue compartment and cell types associated with hypoxic tumour neighbourhoods adjacent to necrotic cores (compartment 6, 11, 3, and basal hypoxic tumour cells), underscoring the importance of local tissue architecture (Supplementary Fig. 27).

Overall, these findings indicate that drug tolerance in residual subpopulations is not directly linked to reduced cisplatin uptake, consistent with previous immunohistochemical analyses using Pt-DNA adduct-specific antibodies^16^.

### Tumour niches share molecular characteristics with MRD in human breast cancer

To translate our findings from the mouse model to human disease, we analysed ST data from human BRCA1-deficient breast cancers to characterise intratumoural heterogeneity and conserved molecular features across species. We profiled primary and post-neoadjuvant residual tumours using ST (*n*_Primary_ = 2, *n*_Residual_ = 3, *n*_Spot_ = 17,340). Tissue sections were first annotated by a pathologist, and capture spots containing tumour tissue were selected for downstream analysis **(**Supplementary Fig. 28a).

**Fig. 5:**
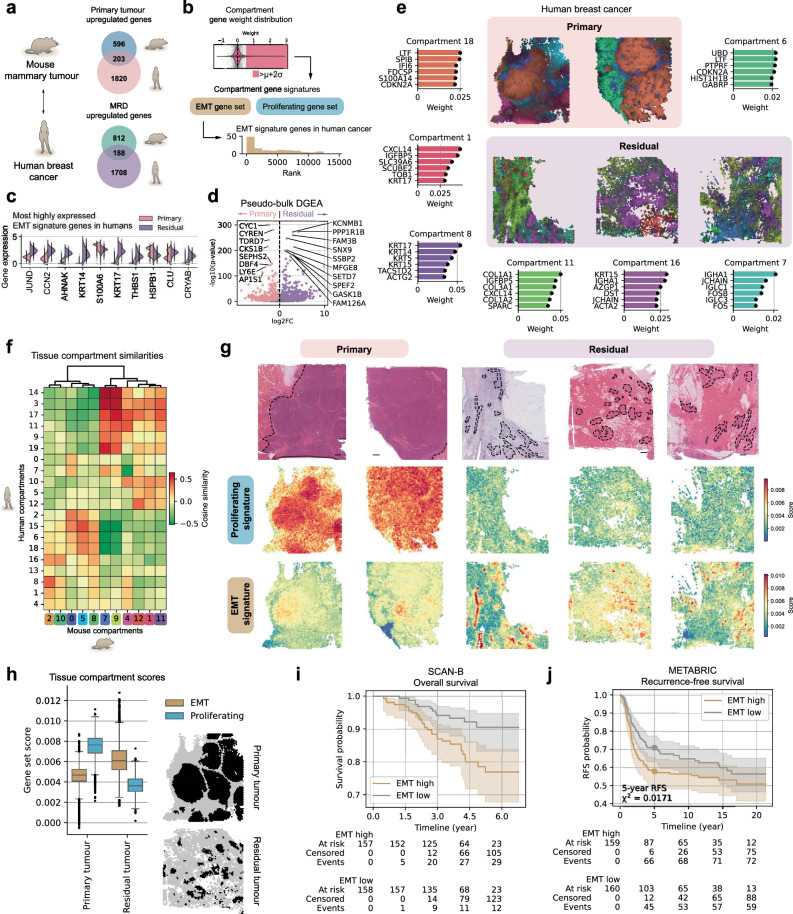
Molecular characteristics of tumour niches are shared with MRD in human breast cancer. **a** Mouse primary tumours share 203 upregulated genes with primary human breast tumours and 188 genes in their residual state. **b** EMT and proliferating compartment gene signatures were determined by taking top-weighted genes exceeding the mean plus two standard deviations. **c** Top ten most highly expressed EMT signature genes in human primary and residual tumours (*n*_Primary_ = 2, *n*_Residual_ = 3). **d** Pseudo-bulk DGEA of primary and residual human breast cancer ST samples (*n*_Primary_ = 2, *n*_Residual_ = 3, *n*_Spot_ = 17,340). **e** MIP of the identified tissue compartments on primary and residual human tumour tissue sections (*n*_Primary_ = 2, *n*_Residual_ = 3, *n*_Spot_ = 17,340). **f** Heatmap showing cosine similarity between the mouse and human cellular niches identified with Chrysalis (*n*_Human_ = 5, *n*_Mouse_ = 25). **g** Spatial visualisation of EMT and proliferative signature scores in primary and residual human tumour tissue sections (*n*_Primary_ = 2, *n*_Residual_ = 3, *n*_Spot_ = 17,340). **h** Distributions of EMT and proliferative signature scores across individual capture spots in primary and residual human tumour tissue (centre line: median, box limits: upper and lower quartiles, whiskers: 1.5x interquartile range, black dots: outliers). Capture spots were identified as tumour-containing based on expert annotations of the corresponding H&E-stained sections (black spots: tumour tissue, light grey spots: non-tumour tissue; statistical significance was assessed using one-way ANOVA with Tukey’s HSD post hoc test confirming significant differences between all pairwise group comparisons, adjusted *p* < 0.001; sample sizes: primary (*n* = 3 tumors, 5,971 spots) and residual (*n* = 2 tumors, 1220 spots)). **i** Kaplan–Meier survival curves of TNBC patients (*n* = 315) from the SCAN-B cohort (solid lines represent the cumulative survival probability, shaded areas denote the 95% confidence interval), stratified by EMT signature expression (log-rank test *p* = 0.0049). **j** Kaplan–Meier analysis of 5-year recurrence-free survival in TNBC patients (*n* = 319) from the METABRIC cohort stratified by EMT signature expression (solid lines represent the cumulative survival probability, shaded areas denote the 95% confidence interval), demonstrating significantly reduced recurrence-free survival in EMT-high tumours (log-rank test *p* = 0.0171). Source data are provided as a Source Data file.

Pseudo-bulk DGEA between primary and residual tumour-associated capture spots identified 2023 upregulated genes in the primary human tumours, of which 203 overlapped with the 799 differentially expressed genes identified in the mouse dataset (Fig. 5a). Conversely, among the 1896 upregulated genes in the human residual tumours and 1000 in the mouse residual tumours, 188 genes were shared between the two species, indicating conserved transcriptional programmes associated with residual disease.

Next, we assessed the conservation of tumour niche-associated transcriptional signatures by applying the signatures derived from the EMT and proliferating tumour niches identified in mouse tumours (Fig. 5b). Of the 120 genes driving the EMT tumour niche in the mouse model, 50 were among the top 10% most highly expressed genes in the human tumours. Several genes within the EMT signature showed marked differences in expression between the primary and residual states (Fig. 5c) and are known drivers of tumour progression in humans, including JUND, which suppresses p53-dependent senescence, and *CCN2*, *AHNAK*, *THBS1*, which modulate cell differentiation, motility, and metastasis.

Consistent with these findings, differentially expressed genes in the pseudo-bulk DGEA (Fig. 5d and Supplementary Data 9) revealed genes upregulated in residual tumours, such as *PPP1R1B*, which has been linked to trastuzumab resistance in HER2-positive breast cancer^69^ and *SNX9*, which promotes metastasis across multiple cancer types^70^. In contrast, upregulated genes in the primary tumours include several cell cycle regulators (e.g., *CYC1*, *CYREN*, *CKS1B*), reflecting the more proliferative state of the untreated disease.

We again leveraged Chrysalis to identify molecular tissue compartments in the human tumours (Fig. 5e and Supplementary Data 10). The majority of the primary tumour tissue (Compartment 6 and 18) was characterised by the expression of genes such as *LTF*, *SPIB*, or *IFI6*, with pronounced immune infiltration captured in Compartment 10, represented by the expression of multiple immunoglobulin genes (Supplementary Fig. 28b). In the residual tumour samples, we characterised three distinct tumour-associated niches (1, 8, 16) composed of malignant epithelial cells, as confirmed by histopathological examination. Additionally, an inflammatory microenvironment (Compartment 11) was observed surrounding subsets of residual tumour islands, exemplified by *CXCL14* expression, and discrete immune cell aggregates (Compartment 7).

Building on this characterisation, we quantified the cellular niche similarity between the mouse MRD model and residual disease in human TNBC (Fig. 5f). Using cosine similarity, we observed moderate alignment of cellular niches across species. Notably, the EMT-associated mouse niche (2) showed the strongest similarity to the human compartment 8 and 16, while the proliferative tumour niche (5) showed parallels with human counterparts 6, 15, and 18. TME-associated compartments also displayed moderate cross-species similarity, with ECM-rich mouse niches (7, 9) showing the strongest alignment, followed by immune-rich niches (12, 1, 11).

To further assess transcriptional concordance, we performed pseudo-bulk DGEA on high-scoring (>0.80) primary (6, 18) and residual (1, 8, 16) tumour-associated tissue compartments. This analysis revealed further similarities with the mouse mammary tumour EMT and proliferating niches, such as downregulated *E2F1* and *MYC* activity and enrichment of the EMT Hallmark gene set in the residual tumours (Supplementary Fig. 29). Notably, pathways related to JAK-STAT, TNFα, and interferon signalling, were downregulated in the human residual tumour niches but upregulated in mice, consistent with the differences in immune infiltration observed between species.

To assess the relevance of the proliferating and EMT expression programmes in human MRD, we evaluated their average expression score in the human ST samples (Fig. 5g). We found elevated EMT signature expression in the residual tumours, correlating with the presence of malignant epithelial cells. Whereas in the proliferating tumours, the signature’s expression was moderately elevated in a limited number of capture spots. Consistently, the proliferating signature was significantly higher across the primary tumour tissue compared to the residual tumour samples. Using the pathologist annotations, we selected capture spots corresponding to primary and residual tumour regions and quantified their expression profiles. This analysis revealed significant changes in the distributions of the proliferating and EMT signature scores between the two treatment groups, confirming differential regulation of these expression programmes in primary versus residual disease (Fig. 5h).

As differences in EMT and proliferating signatures were evident in the spatial datasets, we next examined their clinical relevance at the patient level. We assessed the prognostic value of these signatures using large-scale breast cancer transcriptomic cohorts.

First, we evaluated the association between EMT signature expression and patient outcome in TNBC patients from the Sweden Cancerome Analysis Network - Breast (SCAN-B) cohort^71^ (*n* = 315) (Fig. 5i). Patients were stratified into EMT^High^ and EMT^Low^ groups based on EMT signature expression. Kaplan-Meier analysis revealed significantly poorer overall survival for EMT^High^ patients compared to EMT^Low^ patients, as assessed by the log-rank test.

To further validate the prognostic relevance of the EMT programme in an independent cohort, we analysed TNBC patients from the METABRIC dataset (*n* = 319)^72^, focusing on 5-year recurrence-free survival (RFS). Consistent with the SCAN-B results, EMT^Low^ patients exhibited significantly improved RFS compared to EMT^High^ patients (log-rank test *p* = 0.0171) (Fig. 5j), supporting a link between elevated EMT signature expression and adverse clinical outcome. An additional analysis using the Cancer Genome Atlas Breast Invasive Carcinoma Collection (TCGA-BRCA)^73^ cohort (*n* = 53), incorporating both EMT and proliferating signatures, yielded concordant results (Supplementary Fig. 30).

Together, these analyses demonstrate that the molecular characteristics defining tumour niches in the BRCA1-deficient mouse mammary tumour models closely align with human breast cancer, particularly in residual disease, with shared EMT and proliferative gene expression programmes closely associated with poor clinical outcome, including reduced survival and increased risk of recurrence.

### Targeting a top EMT-signature hit selectively sensitises organoids to chemotherapy

To showcase the translational relevance of the transcriptional signature associated with the EMT-rich tumour compartment, we assessed whether pharmacological inhibition of a top-ranked gene from this signature could synergise with standard chemotherapeutic agents. First, proteins encoded by genes within the EMT signature were cross-referenced against PubChem to identify targets with commercially available inhibitors (Supplementary Data 11). From this list, we selected the highest-weighted gene with an available small-molecule inhibitor, *Lgals3*, encoding Galectin-3 (Gal-3).

**Fig. 6:**
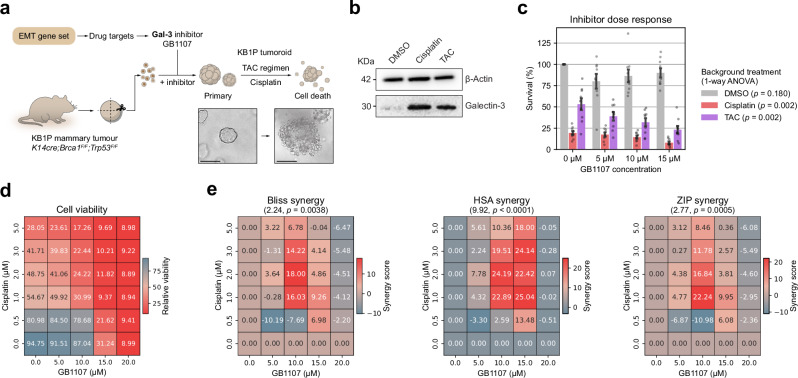
Functional validation of the EMT-niche signature identifies Galectin-3 as a synergistic partner to chemotherapy. **a** KB1P Mouse mammary tumour (*n* = 1) was dissociated and cultured to form tumoroids. For synergy experiments, Gal-3 inhibitor GB1107 was added during tumoroid formation. Tumoroids were subsequently treated with cisplatin or TAC therapy, and cell viability was assessed (scale bar: 200 µm). **b** Effect of chemotherapy treatment on Gal-3 expression measured by Western blotting (representative of 3 experiments). **c** Dose response of GB1107 monotherapy compared to combination with cisplatin or TAC therapy measured with Trypan Blue assay (bar height: mean value, black dots: individual measurement points, error bar: SE; sample sizes: *n* = 3 independent biological replicates, each performed with 3 technical replicates). Statistical significance was determined by one-way ANOVA performed within each treatment background (*p-*values are indicated in the legends). **d** Dose response viability matrix of tumoroids treated with GB1107 and cisplatin, measured with CellTiter-Blue. Values in each tile indicate the mean viability of triplicate measurements (*n* = 3 independent biological replicates, each performed with 3 technical replicates). **e** Synergy matrices representing interactions between GB1107 and cisplatin. Tiles indicate the localised synergy scores, with global scores and *p*-values (one-sample t-test) provided in the subtitles (*n* = 3 biological replicates). Source data are provided as a Source Data file.

Gal-3 is a secreted β-galactoside–binding lectin that has been reported to play important roles in tissue development and tumorigenesis by regulating cell adhesion, proliferation, invasion, and metastasis via binding to cell-surface glycolipids^74–76^. To investigate the relevance of Gal-3 inhibition, we generated mouse mammary tumour organoids from KB1P mice, as previously established^77^. These organoids were pretreated with the selective Gal-3 small-molecule inhibitor GB1107, then treated with cisplatin or the TAC regimen, while maintaining constant GB1107 concentration, in order to assess their potential synergistic effect on tumour cell viability (Fig. 6a).

To determine whether these organoids undergo molecular changes upon chemotherapy that resemble those observed in the EMT niche of tumours, we first analysed Gal-3 protein expression following chemotherapy treatment with Western blotting. The analysis revealed that Gal-3 was weakly detectable in untreated organoids. However, its expression was strongly increased following treatment with both cisplatin and TAC regimen (Fig. 6b), mirroring the enrichment observed in cells residing within the EMT niche in vivo. This provided a basis for testing if pharmacological inhibition of Gal-3 could sensitise the organoids to cisplatin and TAC.

Following the treatment protocol outlined in Fig. 6a, we first evaluated the interaction between the GB1107 and chemotherapy by analysing the effect of inhibitor concentration in the context of TAC (2.5 nM docetaxel, 0.01 μM doxorubicin, 2 μM cyclophosphamide), cisplatin (2 μM), and DMSO treatment backgrounds, using a Trypan Blue-based cell survival readout (Fig. 6c). One-way ANOVA confirmed that the inhibitor alone had no significant effect on cell survival in the DMSO control (*p* = 0.179). In contrast, combining it with the chemotherapy drugs significantly potentiated the cytotoxic effects of both cisplatin (*p* = 0.002) and TAC (*p* = 0.002).

To further characterise the nature of this interaction across a broader range of concentrations, we performed a comprehensive dose-response matrix analysis using a metabolic viability readout (CellTiter-Blue), focusing on cisplatin. The corresponding dose-response matrix revealed a consistent two-dimensional gradient, confirming a dose-dependent reduction of cellular fitness across the entire combination landscape (Fig. 6d). We utilised SynergyFinder+^78^ to calculate synergy scores across four independent mathematical models: ZIP, HSA, Loewe, and Bliss. The analysis confirmed that the interaction is statistically significant across Bliss (*p* = 0.0037), ZIP (*p* = 0.0005), and HSA (*p* < 0.0001) models. While the Loewe model did not reach significance (*p* = 0.796), the high significance observed across models assuming independent mechanisms of action (Bliss/HSA) aligns with the distinct biological roles of DNA damage and Gal-3 inhibition in our model. Bliss synergy revealed a distinct synergy window at 10 μM of GB1107 and 1-3 μM of cisplatin, where the combined effect was more pronounced, similarly to the other models (Fig. 6e).

These findings suggest that targeting genes of the EMT-niche signature, such as *Lgals3*, may provide a potential strategy to enhance the efficacy of chemotherapeutic agents.

## Discussion

Our study presents a comprehensive multimodal framework integrating scRNA-seq, ST, and IMC to resolve the spatiotemporal organisation of MRD in the KB1P mouse model for hereditary breast cancer. By jointly profiling cellular niches, signalling pathways, and key transcriptional regulators, and by demonstrating that they are conserved across mouse and human tumours, our approach enables the systematic identification of MRD-associated molecular programmes with potential therapeutic relevance.

Using sequencing-based analyses in our mouse model, we identify EMT-associated tumour niches as a central feature of residual disease following chemotherapy. While EMT-associated and quiescent drug-tolerant states have been previously linked to therapeutic resistance and adverse prognosis^16,79,80^, our study extends these observations by resolving their spatial organisation, temporal stability, and reversibility during minimal residual disease using longitudinal multimodal profiling. Importantly, the EMT-associated tumour niche was already detectable at low abundance in treatment-naïve primary tumours and persisted with largely unchanged frequency throughout chemotherapy, indicating selection of a pre-existing drug-tolerant state rather than de novo induction by treatment. Residual tumour cells within these niches exhibit a distinct transcriptional programme characterised by genes such as *WFDC2*, *ANXA1*, *SLPI*, *LTF*, and *TACSTD2* (Supplementary Data 5). This programme persists across different chemotherapeutic regimens, as well as in human MRD, where it is associated with adverse clinical outcomes.

EMT has long been implicated in drug resistance through multiple mechanisms, such as increased drug efflux, reduced proliferation, evasion of apoptotic signals, and fostering immune evasion^81–85^. In breast cancer specifically, EMT-associated transcription factors, such as Twist, Snail, and Foxc2 have been shown to upregulate ABC transporters^86^. Further evidence showed that activation of Twist and Snail confers resistance to commonly used chemotherapeutic agents such as doxorubicin and docetaxel^87,88^. In TNBC cell lines, preferential expression of RHOJ in EMT cells enhances the DNA damage response, rendering these cells more resilient to cytotoxic therapy^89^. Consistent with these findings, our data highlight the importance of considering EMT-associated cell states when developing therapeutic strategies for residual disease.

Our spatiotemporal profiling uncovered a rich ecosystem composed of tumour cells together with stromal and immune components of the TME. We identified highly proliferative, basal, and luminal-alveolar tumour cell lineages that transition into dormant EMT phenotypes, which showed significant enrichment during MRD. Both EMT^90^ and cellular dormancy^19,91^ have been shown to be regulated or modulated by the TME, underscoring the importance of studying MRD in an immunocompetent model. Additionally, we identified *Spp1*+ and complement-high (CH) macrophage populations, which have been previously linked to pro-tumour microenvironments^36,92^, and may facilitate the survival of EMT and other tumour cell states or support their re-entry into a proliferative programme. Even though our data do not allow us to determine how or when the dormant EMT phenotype is induced in residual tumour cells, they highlight the contribution of both tumour-intrinsic and tumour-extrinsic factors of therapy response.

Cell type deconvolution further revealed the in situ localisation of these populations, while cellular niche analysis revealed their spatial organisation and distinct temporal dynamics during tumour progression, confirming that the EMT niche remains unaffected by treatment. We observed a high degree of similarity in spatial organisation between primary and recurrent tumours, pointing to the highly dynamic and reversible nature of residual disease. Although the majority of tumour cells are eliminated at the time of maximal response to both platinum-based and combination chemotherapy treatments, surviving residual cancer cells eventually regrow into recurrent tumours that recapitulate the intratumoural heterogeneity of the primary tumours. This process appears to be supported by a permissive TME, characterised by elevated SASP factors, tumour-promoting cell interactions, inflammatory pathways, immunosuppressive macrophages, which, similarly to the tumour compartments, undergo extensive remodelling following treatment. While our analysis does not identify a specific tumour cell subpopulation responsible for recurrence, it provides insight into the heterogeneity and plasticity underlying drug tolerance. Notably, the consistency of these observations across distinct therapeutic regimens suggests that, under these conditions, drug tolerance is associated with a unified transcriptional state rather than being drug specific.

Moreover, we revealed several key pathways and processes driving tumour progression and critical stages of MRD and recurrence, including a steep downregulation of MAPK signalling and an upregulation of JAK-STAT activity. We characterised several functional components attributed to key tumour or TME-associated tissue compartments. Notably, numerous ECM-remodelling and immunosuppressive processes were spatially correlated with persisting cells within the EMT niche, highlighting a supportive TME for drug-tolerant tumour states.

We further developed a multimodal assay to quantify intracellular Pt content and investigate its relationship to transcriptional changes in cisplatin-treated tumours. We confirmed that necrotic regions and fibroblasts serve as major Pt sinks within the tumour tissue. Additionally, macrophages that retained elevated Pt levels two weeks after treatment may contribute to the impaired immune-mediated tumour clearance, consistent with previous studies showing that cisplatin-exposed macrophages can promote EMT programmes in ovarian cancer^93^. Consistent with previous findings^16^, we demonstrated that tumour cells exhibit comparable cisplatin uptake across transcriptional states, indicating that drug tolerance may not arise from heterogeneous distribution of DNA damage across tumour cell populations.

The translational relevance of the EMT niche signature is underscored by its conservation in human clinical samples, where its spatial localisation overlapped with tumour islands found in residual tumours. This clinical significance is further supported by analyses of multiple large-scale TNBC cohorts, where high expression of the EMT signature correlated with significantly poorer overall survival and 5-year recurrence-free survival. To provide functional validation, we focused on one of the highest-weighted components, Gal-3. Western blot analysis revealed that Gal-3 was only weakly detectable in untreated organoids, whereas its expression became strongly enriched following chemotherapy treatment. This shift may indicate a selective survival advantage of the pre-existing EMT subpopulation, consistent with its presence in residual islands. Importantly, we demonstrated statistically validated synergy between Gal-3 inhibition and chemotherapy, which positions our findings as a transcriptional map of functional vulnerabilities.

In conclusion, we present a comprehensive spatiotemporal framework capturing the unique transcriptional states of cancer cells, including chemotherapy-tolerant populations, together with the dynamic remodelling of the TME during MRD and recurrence. By integrating ST and multimodal functional profiling, our study provides a foundation for mechanistic interrogation of MRD-associated cell states and identifies molecular programmes that may be exploited to enhance therapeutic efficacy in TNBC.

## Methods

### Experimental work

#### Collection of mouse mammary tissue

All animal experiments were approved by the Animal Ethics Committee of the canton of Bern (Switzerland) (protocol numbers BE69/2021 and BE60/2023) and conducted in accordance with the current Swiss Acts on Animal Experimentation. Mice were housed in standard IVC cages with bedding, a red mouse house, a tunnel, nestlets, and wood sticks. A maximum of five mice were kept per cage. The light cycle consisted of 12 hours of light and 12 hours of darkness. The room temperature averaged 22 °C (range: 21–24 °C), with a humidity averaging 55% (range: 45–65%). The cages were opened only under a laminar flow hood.

Two independent parental tumours (T1, T2.1-2.2) were used in this study. While they are both derived from the *K14cre;Brca1*^*F/F*^*;Trp53*^*F/F*^ (KB1P) mouse mammary tumour model, Tumour 1 represents a GFP-labelled *Brca1*^*-/-*^*;p53*^*-/-*^ mammary tumour, whereas samples from Tumour 2 were expanded in two different experiments (T2.1 and T2.2).

The DMSO-preserved tumour materials were thawed, washed with PBS, and dissected into small fragments (~2 mm in diameter, approximately the size of a needle head). Tumour fragments were orthotopically transplanted in the fourth mammary fat pad (right side or bilaterally) of 2–4 months old female FVB/NJ mice. Tumour growth was monitored by calliper measurements and the volume was calculated (length × width^2^ / 2). The maximal tumour volume permitted by our ethics committee is 1,000mm^3^, and we confirm that this maximal volume was not exceeded. Animals were randomly assigned to treatment groups (untreated *n* = 10, TAC treatment *n* = 8, cisplatin treatment *n* = 12).

Once tumours reached a volume of approximately 150mm^3^, mice were either euthanised to collect primary tumours or treated with a single dose of chemotherapy. TAC treatment considered a single dose of 2.5 mg/kg doxorubicin (Teva, Pharmacode 4439164) injected intravenously, combined with 12.5 mg/kg docetaxel (Teva, Pharmacode 6984902) and 120 mg/kg cyclophosphamide (Baxter, Pharmacode 2731162), both administered intraperitoneally. Alternatively, mice received a single dose of 6 mg/kg cisplatin (Teva, Pharmacode 4333164) injected intravenously.

Residual tumours were collected 7 (*n* = 2 per treatment) or 12 days (*n* = 4 per treatment) after treatment, while recurrent tumours (*n* = 2 per treatment) were harvested once they reached their original size. For IMC analyses, a subset of cisplatin-treated tumours was collected 4 (*n* = 2) or 24 h (*n* = 2) post-treatment. Animals were euthanised with CO_2_, and tumour measurements were performed by animal technicians blinded to treatment allocation. Mammary fat pads from non-transplanted mice were collected as healthy controls

Tumours were harvested at different experimental endpoints and processed either for tissue-based analysis or for scRNA-seq. For ST and IMC, tissues were fixed immediately after harvesting in 4% paraformaldehyde, trimmed, paraffin-embedded, and stained with H&E for initial histological assessment.

#### Mouse mammary tumour dissociation and FACS

Mice were euthanised, tumours were collected and directly dissociated into single-cell suspensions. First, tumours were washed with PBS, and the surrounding tissue, including blood vessels, fat, and connective tissue, was removed. Tumours were then minced into very small pieces and transferred together with PBS into 15 ml tubes. Samples were centrifuged at 600 × g for 5 min at 4 °C, after which the supernatant was discarded. Tumour fragments were incubated in 8.5 ml of digestion mix (0.02 g collagenase type IV from *Clostridium histolyticum* (Sigma, C5138) in 10 ml of Advanced DMEM/F-12 (Gibco, Thermo Fisher Scientific 12634010) supplemented with a final concentration of 100 m2M HEPES pH 7.5 (Sigma, H4034), 1x GlutaMAX (Gibco, 35050038), 50 units/ml penicillin-streptomycin (Gibco, 15070063)) at 37 °C for 15 min while shaking. The tissue was further mechanically dissociated by gently pipetting up and down. This enzymatic and mechanistic dissociation step was repeated twice.

Next, samples were centrifuged at 600 × *g* for 5 min at 4 °C, washed with supplemented Advanced DMEM/F-12 and centrifuged again. Pellets were resuspended in 4 ml of DNase solution (45 μl DNase I Solution 2500 units/ml (Thermo Fisher Scientific, 90083) in 5 ml supplemented Advanced DMEM/F-12) while gently pipetting up and down for 5 min. Cells were centrifuged again at 600 ×* g* for 5 min at 4 °C, resuspended in 4 ml of the supplemented Advanced DMEM/F-12, and shaken by hand for 2 min. At this stage, the tissue was completely dissociated, if necessary, visible tissue fragments were removed from the liquid fraction with a pipette. Cells were centrifuged one last time at 600 × *g* for 5 min at 4 °C and resuspended in 600-800 μl freezing medium (Recovery™ Cell Culture Freezing Medium, Gibco, 12648010). The suspension was filtered through 40 μm Flowmi^®^ Cell Strainers (Sigma, BAH136800040) into cryogenic tubes. A 10 μl aliquot was taken for manual cell counting to estimate the number of viable single cells prior to freezing. If aggregates were present, cells were centrifuged, resuspended, and filtered again. Cells were frozen and stored at −150 °C until further use.

Frozen vials were thawed and stained with the near-IR fluorescent reactive dye of the LIVE/DEAD™ Fixable Dead Cell Stain Kits (Invitrogen, Thermo Fisher Scientific, L34960) according to the manufacturer’s instructions. All steps were performed protected from light. Tubes and pipette tips were coated with 0.1% BSA (Bovine Serum Albumin Fraction V, Roche, 10735086001) in PBS to prevent cell loss, and a wide-bore pipette was used for gentler handling. Cells were resuspended in a solution of 0.4% BSA in PBS and kept on ice.

Cell sorting was performed using a Beckman Coulter MoFlo ASTRIOS EQ cell sorter with a 120 μm nozzle at 10 psi system pressure, using Beckman Coulter IsoFlow sheath fluid. Sample and collection devices were maintained at 4 °C. Single-stained controls and unstained cells were used to set the gates for the cell viability (ex. 640 nm, em. 795 nm) and GFP expression (ex. 488 nm, em. 526 nm). After exclusion of doublets, contaminating red blood cells, and dead cells, three primary tumours and three residual tumours were sorted based on their GFP expression. Tumour cells were enriched in the GFP-positive population, while cells of the TME in the GFP-negative population. At least 25,000 cells per condition were collected for scRNA-seq.

#### scRNA-seq data generation

Cells were processed immediately after the FACS sorting. ScRNA-seq libraries were prepared at the Next Generation Sequencing Platform of the University of Bern, using the 10x Genomics Chromium Single Cell 3′ Library and Gel Bead Kit v3.1 according to the manufacturer’s standard protocol (cDNA amplification in 14 cycles). A targeted cell recovery of 8000 cells per sample was used. Libraries were sequenced on an Illumina NovaSeq 6000 platform using an S4 flow cell.

#### Collection of human tissue

Primary human breast tissue was obtained from the Division of Clinical Pathology, Geneva University Hospitals (Geneva, Switzerland) and the Department of Pathology, Centre Léon Bérard (Lyon, France), in compliance with local ethical regulations. The study was approved by the local ethical committee of Geneva (Commission Cantonale d'éthique de la recherche Genève: CCER 2019-00004) and Lyon (Ethics Committee of Lyon Sud-Est IV and Institutional local Translational Research Review Committee: AC-2024-6625 and DC-2008-99). Tumour samples (tumorectomy) were obtained from women with germline mutations of BRCA1 with (*n* = 2) or without (*n* = 3) neoadjuvant chemotherapy. The patients provided informed written consent for the use of biological samples and clinical data for research purposes. There was no compensation in accordance with local ethical and legal regulations in France and Switzerland. Participants were not specifically recruited for this study. Sex was determined based on clinical records, only females were included in this study as the research focuses on BRCA1-mutated breast cancer. No sex-based disaggregated analysis was performed due to the single-sex nature of the cohort. FFPE samples were profiled using 10x Genomics Visium.

#### ST data generation

FFPE mouse mammary and human breast tumour tissues were histopathologically assessed, and samples with minor necrosis were selected for further ST profiling. RNA quality control was performed on 32 mouse and 5 human samples. Mouse samples and four human samples were processed following the Visium v1 Gene Expression protocol; one human sample was processed using the Visium v2 CytAssist workflow.

Briefly, 5 μm-thick sections were placed onto Visium slides. Sections were manually H&E stained and scanned using a S360 Nanozoomer (Hamamatsu) at 40x magnification. cDNA libraries were generated according to the Visium Gene Expression User Guide (10x Genomics) and sequenced at the Next Generation Sequencing Platform at the University of Bern on an Illumina NovaSeq 6000 system using different flow cells with 100-300 cycles.

#### Histopathological assessment

Whole-slide H&E-stained images from mouse mammary tumours and the human breast tissue samples were used for histopathological assessment. All slides were scanned on a Nanozoomer S360 (Hamamatsu) at 40x magnification (0.23 µm/pixel resolution). H&E images corresponding to Visium tissue sections were examined and annotated using QuPath^94^ (version 0.4.3). Regions of necrosis, lymph nodes, and surrounding adipose tissue were annotated and excluded from downstream ST analyses.

#### IMC staining and measurement

FFPE cisplatin-treated tumours sections were used for both ST and IMC analyses. First, the sections of 5 μm were cut for ST, directly followed by serial 3.5 μm sections for IMC. An additional 3.5 μm section was cut for H&E staining to assess the tissue morphology and define ROIs for IMC. Slides were processed and stained as previously described^95^.

Briefly, FFPE sections mounted on Superfrost Plus slides were dewaxed in xylol, rehydrated through graded ethanol (100%, 94%, and 70%), and rinsed in distilled water. Heat-induced epitope retrieval was performed in a Decloaking Chamber™ NxGen (Biocare Medical, DC2012) at 110 °C in citrate buffer (pH 6.0, Sigma, C9999) for 15 min. After cooling, sections were washed with PBS and blocked for 1 h in a hydration chamber at room temperature using protein blocking (Tris-buffered saline 1x pH 7.5, 0.1% NaN_3_ (Merck, 106688), 3% Goat serum (Thermo Fisher Scientific, 16210-064), 0.5% Casein (Sigma, C-8654), 0.025% Tween20 (Sigma, P1379)).

Samples were incubated overnight at 4 °C with a metal-conjugated antibody cocktail (19 antibodies, Supplementary Data 7). After washing twice with PBS containing 0.05% Tween-20 and twice with PBS alone, sections were incubated with a 1:100 dilution of 25 μmol/L Ir-Intercalator (Fluidigm, 201192 A) for 30 min at room temperature. Samples were quickly rinsed in distilled water and air-dried prior to acquisition.

IMC data were acquired using the Hyperion™ Tissue Imager (Standard BioTools). Tissue was ablated using a 1 μm-diameter UV laser operating at 200 Hz within the selected ROIs. The ablated material was transported into a Helios mass cytometer, where elements were atomised and ionised in an inductively coupled plasma and analysed in a time-of-flight (TOF) mass analyser based on their mass-to-charge ratios. Mass cytometry data (MCD files) were acquired using CyTOF software version 7.0.8493. In total, 44.4 mm² of tumour tissue was acquired and analysed across seven samples.

#### Three-dimensional cell culture

Three-dimensional cell culture and tumoroid assays were performed using KB1P tumoroids derived from *Brca1*^*−/−*^*;Trp53*^*−/−*^ mouse mammary tumours and cultured as described previously^77^. Cells were seeded in 24-well plates embedded in 40 µl Cultrex Reduced Growth Factor Basement Membrane Extract Type 2 (BME; Trevigen Bio-Techne, catalogue no. 3533-010010) and growth media (1:1) and cultured in Advanced DMEM/F12 medium (AdDMEM/F12, Gibco, catalogue no. 11550446) supplemented with 50 U/mL penicillin/streptomycin (Gibco, catalogue no. A9165), 1 mol/L HEPES (Sigma, catalogue no. H0887), GlutaMAX (Gibco, catalogue no. 35050061), B27 (Gibco, catalogue no. 17504044), 125 μmol/L N-acetyl-L-cysteine (Sigma, catalogue no. A9165), and 50 ng/mL murine EGF (Sigma, catalogue no. E4127). The organoids were cultured at 37 °C in the presence of 5% CO_2_ and regularly tested for Mycoplasma contamination.

#### Culture for tumoroid regrowth model

For the tumoroid regrowth model, single cells were generated using TrypLE (Gibco) and seeded at 50,000 cells per 40 μl drop in a 24-well plate. On day 4 post-splitting, tumoroids were treated with either cisplatin or TAC therapy or DMSO as control. For cisplatin (Teva, Pharmacode 4333164) a concentration of 2 μM was used. For TAC therapy, a combination of 2.5 nM Docetaxel (Teva, Pharmacode 6984902), 0.01 μM Doxorubicin HCl (Selleckchem, S1208), and 2 μM 4-Hydroperoxy-cyclophosphamide (NIOMECH, D18864) was used. Tumoroids were treated for 24 hours before the media was changed.

For combination treatments, the Galectin-3 small-molecule inhibitor GB1107 (MedChemExpress, HY-114409) was used. Briefly, 24 h and 72 h post-trypsinisation, cells were pre-treated with GB1107 (5 μM, 10 μM, and 15 μM), followed by the indicated concentrations of cisplatin on day 4 for 24 h before media change. Six days after drug removal, tumoroids were trypsinised and the viable single cells were counted using Trypan Blue Stain 0.4% (Gibco, 15250-061) and a LUNA II automated cell counter (Logos). Alternatively, cell viability was measured using the CellTiter-Blue Cell Viability Assay (Promega G8081) following manufacturer’s instructions.

#### Western blotting

Western blotting was performed on tumoroids collected six days after Cisplatin or TAC treatment. Cells were lysed in RIPA buffer (50 mmol/L Tris-HCl pH 7.4; 1% NP-40; 0.5% Na-deoxycholate; 0.1% SDS; 150 mmol/L NaCl, 2 nmol/L EDTA, 50 mmol/L NaF) supplemented with 1x complete protease inhibitor cocktail (Roche, catalogue no. 04693132001) for 60 min on ice, followed by homogenisation with a syringe and needle. Lysates were centrifuged at 14,000 rpm for 10 min, and the supernatant was collected to assess the protein concentration using the Pierce BCA assay kit (Thermo Fisher Scientific, catalogue no. 23225). Protein lysates were denatured at 95 °C for 5 min in 6x SDS sample buffer [Laemmli SDS sample buffer, reducing (6x); Thermo Fisher Scientific, catalogue no. J61337.AC] and separated by SDS-PAGE on 12% acrylamide gels. Subsequently, proteins were wet-transferred for 1 h at 100 V to 0.45 μm pore size polyvinylidene difluoride membranes (GE Healthcare, catalogue no. 10600018). Membranes were blocked in 5% BSA in Tris-buffered saline with Tween-20 (TBS-T, 100 mmol/L Tris, pH 7.5, 0.9% NaCl, 0.05% Tween-20) and then incubated with the following primary antibodies: anti-Galectin 3 rat monoclonal (1:1000, Invitrogen, catalogue no. 14-5301-82, RRID: AB_837132) and anti-beta Actin (1:1000, mouse monoclonal Sigma, catalogue no. A1978, RRID: AB_476697) in blocking buffer for 2 h at room temperature. After washing in TBS-T, the following horseradish peroxidase (*HRP*)-linked secondary antibodies were used: anti-mouse IgG (1:2500, Cell Signaling Technology, catalogue no. 7076, RRID:AB_330924) and anti-rat IgG (1:2500, DAKO catalogue no. P0450), applied for 1 h at room temperature. Images were acquired using the FUSION FX7 imaging system (Vilber GmbH).

### Computational analysis

#### scRNA-seq data processing and cell phenotyping

Read mapping and counting of the 3 primary and 3 residual tumour libraries (processed in a single sequencing run) were performed with CellRanger 6.1.2 (10x Genomics), using the mm10 reference genome for *Mus musculus*. Downstream analysis was carried out using SCANPY^96^ in a Python v3.9-based environment.

Quality control was performed by removing low-quality cells based on thresholds (Supplementary Data 12) defined by total gene count, number of expressed gene distributions (calculated with scanpy.pp.calculate_qc_metrics), as well as mitochondrial gene percentage (<5%). This resulted in 8470 cells from the control tumours (5665 tumour cells and 2805 cells from the microenvironment) and 9502 cells from the residual tumours (1384 tumour cells and 8118 cells from the microenvironment).

After integration in SCANPY, major cell types were manually annotated based on top-ranked genes identified using the Wilcoxon test (scanpy.tl.rank_genes_groups) applied to Leiden clustering results (scanpy.tl.leiden, resolution = 0.2). Cell subpopulations were identified either by subclustering major cell type clusters (scanpy.tl.leiden, resolution = 0.6) or, for T cells, by algorithmic T cell detection using TiLPRED^97^. Tumour cell populations and lineages were characterised using Wilcoxon tests in combination with previously published gene signatures^34,35^.

For visualisation, t-SNE embeddings were generated in SCANPY using the normalised, log1p transformed count matrix. Dimensionality reduction was performed with scanpy.tl.pca (svd_solver = ‘arpack’), followed by neighbourhood graph construction (scanpy.pp.neighbors) and t-SNE computation (scanpy.tl.tsne) using default parameters.

#### Pseudo-bulk DGEA

Pseudo-bulk profiles from tumour cells of the 3 primary and 3 residual scRNA-seq samples were generated by aggregating raw counts using decoupleR^98^. Lowly expressed genes were filtered using decoupler.plot_filter_by_expr. Differential gene expression analysis was performed with PyDESeq2^99^, with the Benjamini-Hochberg procedure to control the false discovery rate. Log2 fold changes were corrected with the apeglm^100^ shrinkage method. Differentially expressed genes were classified based on adjusted *p-*values (*p* < 0.05) and absolute log2 fold change values (> 0.5).

DGEA of ST mouse mammary tumour samples (primary *n* = 10, residual *n* = 9, samples used for multimodal IMC analysis were excluded) was performed by aggregating raw counts from all capture spots classified as tumour by histopathological annotation. This analysis included both cisplatin- and TAC-treated tumours, as well as both residual tumour time points (7 dpt, 12 dpt).

For human samples (primary *n* = 2, residual *n* = 3), DGEA was performed similarly by aggregating raw counts from all capture spots classified as malignant epithelium by expert pathologists.

#### RNA velocity

Velocyto^101^ (velocyto.run10x) was applied to all six samples to quantify the ratio of spliced and unspliced mRNAs using BAM files generated by CellRanger. RNA velocity analysis was performed using scVelo^102^ on the resulting loom files to infer latent cellular dynamics. Highly variable genes were selected using, scvelo.pp.filter_and_normalize (min_shared_counts = 20, n_top_genes = 2000) and neighbourhood moments were computed with, scvelo.pp.moments (n_pcs = 30, n_neighbors = 30). Splicing kinetics were modelled using scvelo.tl.recover_dynamics, and latent time was inferred with scvelo.tl.latent_time. Velocity results were visualised on the original tumour cell t-SNE embedding.

#### ST data processing

FASTQ files were processed with SpaceRanger (10x Genomics) *count*. Sequence reads were mapped to the mm10 (mouse) and GRCh38 (human) reference transcriptomes using the Mouse Transcriptome v1 and Human Transcriptome v1 probe sets, respectively. Loupe Browser (10x Genomics) was used for manual fiducial frame alignment and manual selection of tissue-covered spots.

Quality control was performed with SCANPY based on inspection of spatial and global distributions of the total counts and number of detected genes across capture spots. Sample-specific manual thresholds were applied to remove low-quality capture spots (insufficient number of counts, oversaturated capture spots due to lateral diffusion). Genes expressed in fewer than 10 capture spots were excluded (Supplementary Data 13 and Supplementary Data 14). Normalisation and log-transformation were performed with scanpy.pp.normalize_total (target_sum = 1e4, exclude_highly_expressed = True) followed by scanpy.pp.log1p.

Histopathological annotations were mapped to capture spots by querying QuPath project files using the Paquo (Bayer AG) Python package. Annotation polygons were extracted and matched to capture spots using the Shapely Python package by determining whether the centroid of each capture spot fell within a polygon. For improved visualisation of H&E images, OpenCV2 Python package was used to generate segmentation masks for background removal with contour detection (cv2.Canny, cv2.dilate, cv2.erode, cv2.findContours).

#### Cell type deconvolution

Cell-type deconvolution was performed using cell2location^103^. This probabilistic model integrates scRNA-seq reference data with spatial transcriptomics to map cell-type-specific expression patterns and estimate the abundance of cell types at each spatial location. Our scRNA-seq dataset was used to train the single-cell regression model and derive cell type-specific expression signatures.

Genes were first filtered (cell_count_cutoff = 5, cell_percentage_cutoff2 = 0.03, nonz_mean_cutoff = 1.12). The regression model was trained with a maximum number of epochs set to 1000. The cell2location model was subsequently trained (N_cells_per_location = 30, detection_alpha = 20) using a maximum of 3000 epochs.

Per-spot cell densities were calculated by dividing the cell type abundance values (Supplementary Data 2) by the total number of inferred cells per capture spot. Sample-level cell-type fractions were obtained by summing cell densities across all capture spots for each cell type and normalised by the total sum of all cell-type proportions, yielding values representing the contribution of each cell type to the overall tissue composition.

#### Cellular niche inference

Cellular niches (tissue compartments) were inferred by Chrysalis^43^ on Visium ST data. Chrysalis builds on archetypal analysis to identify spatially and functionally distinct tissue compartments. It operates by fitting a simplex to a latent representation of the gene expression matrix derived from principal components. The vertices of the simplex correspond to archetypes, which represent distinct biological programmes and define tissue compartments. Chrysalis decomposes the count matrix into a basis matrix containing a set of tissue compartment scores that sum up to one per spot, and a weight matrix describing the contribution of each gene to each compartment.

For the mouse mammary tumour dataset, spatially variable genes (SVGs) were on normalised and log-transformed data using chrysalis.detect_svgs (min_morans = 0.025, min_spots = 0.05). Samples were then integrated using HarmonyPy (Python implementation of Harmony^104^) with sample ID as a covariate. Principal component analysis (PCA) was performed on the SVG matrix (chrysalis.pca), followed by archetypal analysis (chrysalis.aa). Thirteen tissue compartments were selected based on the elbow point of the reconstruction error curve. Two compartments were excluded following inspection of gene weight distributions and spatial localisation, as they contained very few positively weighted genes and were likely driven by technical artefacts. This was further verified by inspecting the spatial localisation of these compartments.

For the human dataset, integration was performed using Scanorama^105^ after normalisation and log-transformation. This approach was chosen to better account for large interpatient heterogeneity by preserving local structures through graph-based matching rather than enforcing global alignment. Archetypal analysis was performed with n_archetypes = 20, selected using the reconstruction error heuristic.

Tissue compartments were annotated based on their top contributing genes, spatial localisation patterns, corresponding H&E image tiles (Supplementary Fig. 31), and correlation with cell-type density estimates from cell2location. Correlations were calculated using a pairwise Pearson correlation between inferred cell-type densities and tissue compartment scores. Sample-level compartment proportions were computed by summing compartment scores across all capture spots for each tissue compartment and normalising by the total sum of all compartment scores.

Comparison between human and mouse cellular niches was performed by first mapping the mouse genes to the human orthologs using a human-mouse mapping table from BioMart, followed by intersecting SVGs across species. Pairwise cosine similarity between compartments was calculated using the Scikit-learn^106^ implementation (metrics.pairwise.cosine_similarity).

#### Functional characterisation of mammary tumours

Pathway activities were inferred using PROGENy^44,98^, a curated collection of pathway-responsive genes, via decoupleR. The activity of 14 pathways across all capture spots was estimated using a multivariate linear model (decoupler.run_mlm), with the top 500 genes ranked by pathway responses in mice.

Hallmarks of Cancer gene sets were acquired from the original publication^47^. Mouse gene sets were constructed by mapping human genes to mouse orthologues using BioMart. Gene set enrichment scores were calculated for each capture spot using SCANPY’s gene set scoring function (sc.tl.score_genes). The same procedure was applied to calculate SASP^50^, adaptive mutability gene sets^52^. The cell cycle activity gene set^107^ was constructed by combining commonly overexpressed genes in specific cell cycle phases to provide an approximate measure of overall cell cycle activity.

Hallmarks gene set scores from MsigDB^53^ were calculated using Over Representation Analysis (ORA) via decoupleR. Gene sets were considered significant if the corresponding *p-*values were less than 0.05 in more than 90% of capture spots.

Enrichment of GO terms^54^ in cellular niches was calculated with ORA using the gene expression programmes of each niche. Genes were included if their weight exceeded the mean plus two standard deviations (niche signatures are listed in Supplementary Data 5). Enrichment scores were scaled, and the top three terms were visualised as a heatmap.

Mean pathway activities, Hallmarks of Cancer, SASP, adaptive mutability, Hallmarks, and GO gene sets were calculated by averaging across all capture spots for each condition and visualised as heatmaps. Associations with cellular niches were inferred by calculating pairwise Pearson correlations between tissue compartment scores and gene set activity.

Pseudo-bulk DGEA-based functional analysis was performed on capture spots dominated by the mouse EMT or proliferating niches, as well as human primary and residual tumour-containing niches. Spots with compartment scores ≥0.8 we selected. DGEA was performed as described in the *Pseudo-bulk DGEA* section with filtering parameters: mouse: (min_count = 10, min_total_count = 200), human: (min_count = 300, min_total_count = 200). The resulting gene-level statistics were further used to perform enrichment analyses. Transcription factor activity was inferred with decoupleR using a univariate linear model (decoupler.run_ulm) using the CollecTRI^108^ resource containing curated transcription factor-target interactions. Pathway activities were calculated using PROGENy (decoupler.run_mlm), and Hallmarks enrichment was calculated using ORA (decoupler.get_ora_df) on differentially expressed genes (adjusted *p* < 0.05, |log2FC | > 0.5).

Ligand-receptor interactions were analysed from the spatial transcriptomics dataset using LIANA + ^55^. Mouse genes were first mapped to human orthologues to enable cross-species analysis, and low-confidence mappings were removed. For each sample, spatial neighbours were calculated (li.ut.spatial_neighbors, bandwidth = 200, cutoff = 0.1, kernel = ‘gaussian’), and per-spot ligand-receptor interactions were computed using local Moran’s R statistics (li.mt.bivariate, local_name = ‘morans’). The resulting interaction datasets were merged across samples, including their spatial connectivity information. Top niche-specific ligand-receptor interactions were identified by calculating Pearson correlations between cellular niches from Chrysalis and interaction scores. The top interactions were visualised using a matrix plot, highlighting the compartment-specific ligand-receptor activity across the tumour architecture.

#### CNV inference

CNV in the ST samples was inferred using inferCNVpy, a Python implementation of the inferCNV^109^. Genomic positions of genes were identified from the mm10 mouse genome, and CNV scores were calculated on the normalised and log-transformed data using infercnvpy.tl.infercnv (window_size = 100, step = 10, chunksize = 100) on capture spots from healthy epithelial cells from control samples, as well as immune, muscle, and stromal cells as references. CNV profiles were grouped by disease condition (primary, residual, recurrent) and parental tumour lines. To generate CNV clusters, the default clustering workflow was performed with inferCNVpy (PCA, neighbourhood graph computation, Leiden clustering). CNV cluster composition across tissue compartments and inferred cell types was visualised with stacked violin plots (scanpy.pl.stacked_violin).

#### Cell type colocalisation

Cell type colocalisation was quantified by calculating pairwise Pearson correlations between cell-type densities inferred with cell2location, and clusters of co-localising cell types were defined with hierarchical clustering (using SciPy’s^110^ cluster.hierarchy.linkage function with the Ward variance minimisation algorithm using a height threshold of 3.5. Temporal flow of cell-cell colocalisation clusters was visualised with a Sankey diagram.

#### IMC data co-registration and processing

IMC data were co-registered with ST data from serial tissue sections (Supplementary Data 15). For each sample pair (*n* = 7), manual landmark points (20-40) were selected on the Visium H&E and the optical microscopy panorama that was captured during the IMC protocol prior to tissue ablation. These landmark point pairs were used to estimate an affine transformation matrix via the RANSAC algorithm (cv2.estimateAffine2D, parameters: cv2.RANSAC, ransacReprojThreshold = 100), which was subsequently applied to map Visium capture spot coordinates to the IMC space

Multimodal datasets were constructed using the SpatialData^111^ Python library, integrating Visium capture spots with the 26 IMC channels corresponding to the detected heavy metal isotopes extracted from the MCD files (see Supplementary Data 7 for antibody mapping to isotope channels).

IMC images were preprocessed to remove hot pixels with a median filter (3×3 sliding window), replacing pixels exceeding the local median plus a threshold (default 100) with the median. Cell segmentation was performed using Mesmer^112^, a deep learning-based algorithm specifically designed for multiplexed tissue imaging. Mesmer predicted segmentation masks using two input channels: one assigned to cell nuclei (constructed by aggregating the following channels: H3/^176^Yb, DNA1/^191^Ir, DNA2/^193^Ir), and one assigned to cytoplasms (vimentin/^150^Nd, e-cadherin/^158^Gd, pan-actin/^175^Lu). Cell contour polygons were generated with the marching squares algorithm implemented in the scikit-image^113^ Python library (skimage.measure.find_contours, level = 0.5).

Cell phenotyping was performed using Pixie^63^, a pixel-clustering pipeline for quantitative annotation of pixel-level features through unsupervised clustering. Pixie employs Self-organizing Map (SOM), to identify pixel-level clusters, which are subsequently refined through hierarchical clustering. Pixel clusters were mapped to segmented cells, resulting in cell-level clusters that were classified based on input channel intensities. Selected IMC channels containing cell-type markers were preprocessed by applying Gaussian blurring, normalisation of pixel intensities across channels, and empty pixel removal using create_pixel_matrix from ark.phenotyping.pixie_preprocessing. This was followed by SOM training (train_pixel_som from ark.phenotyping.pixel_som_clustering), pixel clustering (cluster_pixels from ark.phenotyping.pixel_som_clustering), and hierarchical clustering (pixel_consensus_cluster from ark.phenotyping.pixel_meta_clustering) to generate pixel clusters.

Cell-level clustering was performed by training a cell SOM (train_cell_som from ark.phenotyping.cell_som_clustering), clustering cells (cluster_cells from ark.phenotyping.cell_som_clustering), and applying hierarchical clustering (cell_consensus_cluster from ark.phenotyping.cell_meta_clustering). Cell-level clusters were manually refined to generate the final cell-type labels.

#### IMC data analysis

IMC measurements produced highly consistent raw pseudocount profiles across ROIs. Nevertheless, detectable variation remained in the distributions of measured heavy metal isotope intensities. To ensure accurate quantification of intracellular Pt intensities, Pt distributions across ROIs were equalised. A cell neighbourhood graph was constructed with Squidpy’s^114^ gr.spatial_neighbors (radius = (0, 40), coord_type = “generic”, delaunay = False) function, based on a spatial proximity of 40 µm jointly across all ROIs for each sample. For each ROI, a border region was defined by shrinking its bounding box by a 25 µm margin, thereby restricting the correction to cells located near ROI boundaries. Cells within this margin were used to evaluate differences in Pt intensity between neighbouring ROIs. The Pt intensity of all cells within each ROI was compared to the average Pt intensity of cells in neighbouring ROIs, with differences weighted by the number of neighbouring cells. A normalisation factor for each ROI was then calculated by comparing its weighted average Pt intensity to the global mean, and these factors were applied to scale Pt intensity values.

Intracellular Pt concentration was calculated by summing the corrected raw counts across all Pt channels within each cell segmentation mask and dividing by the cell area. Log transformation was performed with scanpy.pp.log1p. Tumour cells were classified based on intracellular Pt concentration as follows: Pt^low^ cells were defined as those below the 15th percentile, Pt^mod^ cells as those between the 15th and 85th percentiles, and Pt^high^ cells as those above the 85th percentile of the log-transformed Pt concentration distribution. GMM-based cutoff for perinecrotic tumour cells was determined using mixture.GaussianMixture from the Scikit-learn Python package with two components.

Pt concentration was averaged per cell type across conditions and visualised as a heatmap. Ki-67^high^ and Ki-67^low^ tumour cells were classified using a cutoff determined by fitting a GMM to the log-transformed intracellular Ki-67 concentration.

To correlate the cell types inferred by cell2location with intracellular Pt concentrations, the centroids of mapped Visium capture spots were expanded into discs with a diameter of 55 µm. Mean Pt concentration was calculated across all cells within each disc and pairwise Pearson correlations were subsequently calculated between the mean Pt concentration and the inferred cell-type densities across conditions.

The contribution of molecular features to intracellular Pt content was quantified using MISTy^68^ via LIANA + ^55^. MISTy is an explainable machine learning framework that models interactions between features in a spatial context and evaluates the contribution of distinct interaction sources, referred to as views. Custom views were constructed for predictor variables (cell2location-inferred cell types, PROGENy pathway activities, Chrysalis compartments, cell cycle activities) using liana.ut.spatial_neighbors with bandwidth = 500 and cutoff = 0.1. Log-transformed Pt concentration was used as a target variable and modelled as an intra-view. A random forest model was trained with the bypass_intra parameter set to True. *R*^2^ for each predictor variable set were visualised as stacked bar plots, and the scaled importance of individual features was visualised as rank plots.

#### Niche signatures in human breast cancer

EMT and proliferating niche signatures were preprocessed by mapping mouse gene signatures to human orthologues and filtering out genes that were not expressed in the human samples. Gene set expression scores were calculated in the human ST samples with scanpy.tl.score_genes. Score distributions were subsequently assessed in selected capture spots annotated as tumour by pathologists.

#### Survival analysis

SCAN-B^71^ and TCGA-BRCA^73^ breast cancer patient cohorts were first filtered according to receptor expression status. Provided metadata were verified with GMM-s to create cutoffs for the expression of ER, PR and HER genes to identify TNBC patients.

In the SCAN-B cohort, this resulted in 315 TNBC patients. These patients were stratified according to their EMT expression signature calculated with scanpy.tl.score_genes to EMT^High^ (*n* = 157) and EMT^Low^ (*n* = 158) groups using a cut-off set at the median of the EMT signature distribution. Survival analysis was performed using the lifelines^115^ Python package. The Kaplan-Meier estimator (lifelines.KaplanMeierFitter) was used to estimate the survival functions, followed by a log-rank test (lifelines.statistics.logrank_test) to assess statistical differences between survival distributions, resulting in a *p-*value < 0.005 between the examined groups.

In the METABRIC^72^ cohort, predefined receptor expression status was used to filter TNBC samples, resulting in 319 patients. Stratification was performed identically to SCAN-B, yielding EMT^High^ (*n* = 159) and EMT^Low^ (*n* = 160) groups. Five-year recurrence-free survival was analysed using survival_difference_at_fixed_point_in_time_test.

In the TCGA-BRCA cohort, 163 TNBC patients were identified. Patients were stratified based on a combination of EMT and proliferating expression signatures. For each signature, a gene expression matrix was constructed using the genes comprising the respective signatures. PCA (sc.pl.pca) was performed on each matrix, followed by K-means clustering (sklearn.cluster.KMeans, n_clusters = 2). Patients were stratified based on the combination of EMT and Proliferating clusters, resulting in EMT^High^-Proliferating^Low^, EMT^Low^-Proliferating^High^, EMT^Low^-Proliferating^Low^, and EMT^HIgh^-Proliferating^High^ groups. The Kaplan-Meier estimator (lifelines.KaplanMeierFitter) was used to estimate the survival functions between EMT^High^-Proliferating^Low^ (*n* = 19), and EMT^Low^-Proliferating^High^ (*n* = 34) groups, followed by a log-rank test (lifelines.statistics.logrank_test) to assess statistical differences between survival distributions, resulting in a *p-*value < 0.005.

### Statistics and reproducibility

Statistical methods for each analysis are described in their respective sections. Pearson correlation coefficients were computed using the pearsonr function from SciPy’s stats module. One-way ANOVA was implemented in pingouin package, with cell type proportions used as dependent variables and experimental condition as the between-group factor. Where significant effects were detected, pairwise differences between conditions were evaluated using Tukey’s HSD test. Statistical validation and modelling of drug synergy experiments were conducted with SynergyFinder+^78^. Visualisations, including scatterplots, box plots, violin plots, bar plots, KDE plots, heatmaps, and line plots, were generated using matplotlib^116^, SCANPY, ausankey, lifelines, chrysalis, and seaborn^117^. Mice were randomly allocated to experimental groups to ensure unbiased distribution. Investigators were blinded to treatment status during the experiments and unblinded during the analysis phase. No statistical method was used to predetermine the sample size. Instead, sample size was determined based on empirical considerations and cost constraints. Data were excluded from the analyses only based on pre-established quality metrics, as described in the respective Methods sections.

### Reporting summary

Further information on research design is available in the Nature Portfolio Reporting Summary linked to this article.

## Supplementary information

Supplementary Information

Description of Additional Supplementary Information

Supplementary Data 1

Supplementary Data 2

Supplementary Data 3

Supplementary Data 4

Supplementary Data 5

Supplementary Data 6

Supplementary Data 7

Supplementary Data 8

Supplementary Data 9

Supplementary Data 10

Supplementary Data 11

Supplementary Data 12

Supplementary Data 13

Supplementary Data 14

Supplementary Data 15

Reporting Summary

Transparent Peer Review file

## Source data

Source Data

## Data Availability

All raw RNA sequencing data (scRNA-seq and ST) have been deposited in the Gene Expression Omnibus (GEO) database under accession number GSE299631. All processed ST (10x Visium), scRNA-seq (10x Chromium), and IMC (Hyperion) data, together with the supplementary data required to reproduce the analyses presented in this study, are available through the following Zenodo repositories (ST: 15102983^118^, scRNA-seq: 15103411^119^, IMC co-registered with Visium: 15096025^120^). The deposited datasets include count matrices (Space Ranger and Cell Ranger outputs), AnnData objects containing cell type deconvolution results, tissue compartment inference, histopathological annotations, and gene set signatures, as well as raw and processed IMC acquisitions stored as SpatialData Zarr archives. The publicly available mouse mammary scRNAseq data used in this study are available in the Gene Expression Omnibus database under accession codes GSE149949^34^ and GSE150580^35^. Survival data for the SCAN-B, METABRIC, and TCGA cohorts were derived from their respective original publications and public repositories, specific accession details can be found within those primary sources^71–73^. Source data are provided with this paper. The remaining data are available within the Article, Supplementary Information or Source Data file. Source data are provided with this paper.

## Code availability

The code used for the data analyses presented in this article is available on GitHub at (https://github.com/rottenberglab/residual-disease^121^).
