# Supplementary material for: Spatiotemporal organisation of residual disease in mouse and human BRCA1-deficient mammary tumours and breast cancer
Source: Nat Commun. 2026 Jun 11;17:7456. doi: 10.1038/s41467-026-74125-6 (PMC13408813; doi:10.1038/s41467-026-74125-6)
Supplement: Supplementary file 1 — Supplementary Information [file 41467_2026_74125_MOESM1_ESM.pdf]

Spatiotemporal organisation of residual disease in mouse and human BRCA1-deficient mammary tumours and breast cancer

Demeter Túrós[1,2,‡], Morgane Decollogny[1,2,‡], Anna Moyseos[1,2], Astrid Chanfon[1,3], Myriam Siffert[1], Joanne Bousmar[1,2], Lou Romanens[4], Jean-Christophe Tille[5], Olivier Tredan[6,7], Intidhar Labidi-Galy[4,8], Alberto Valdeolivas[9], Sven Rottenberg[1,2,†]

[1]Institute of Animal Pathology, Vetsuisse Faculty, University of Bern, Bern, Switzerland
[2]Bern Center for Precision Medicine (BCPM), Department for BioMedical Research, University of Bern, Bern, Switzerland
[3]COMPATH, Institute of Animal Pathology, Vetsuisse Faculty, University of Bern, Bern, Switzerland
[4]Department of Medicine and Center of Translational Research in Onco-Hematology, Faculty of Medicine, University of Geneva, Swiss Cancer Center Leman, Geneva, Switzerland
[5]Division of Clinical Pathology, Department of Diagnostics, Hôpitaux Universitaires de Genève, Geneva, Switzerland
[6]Department of Medical Oncology, Centre Leon Berard, Lyon, France
[7]CRCL UMR INSERM 1052-CNRS 5286, Lyon, France
[8]Department of Oncology, Hôpitaux Universitaires de Genève, Geneva, Switzerland
[9]Roche Pharma Research and Early Development, Roche Innovation Center Basel, F. Hoffmann-La Roche Ltd, Basel, Switzerland

[‡]These authors contributed equally to this work.

[†]Correspondence to:
sven.rottenberg@unibe.ch

Supplementary Figures 3
Supplementary Fig.1 | Tumour growth in the KB1P mouse model 3
Supplementary Fig.2 | Cell-type marker genes in scRNA-seq 4
Supplementary Fig.3 | Cell-type composition in primary and residual tumours 5
Supplementary Fig.4 | Spatiotemporal histopathological changes during MRD 6
Supplementary Fig.5 | Histopathological annotations of capture spots 8
Supplementary Fig.6 | Sample-wise changes in tumour cell composition following treatment 9
Supplementary Fig.7 | Changes in cell-type composition during tumour progression 10
Supplementary Fig.8 | Pseudo-bulk DGEA of spatial transcriptomics 11
Supplementary Fig.9 | Cellular niche composition during MRD 12
Supplementary Fig.10 | Cellular niches identified by Chrysalis 13
Supplementary Fig.11 | Spatiotemporal dynamics of cellular niches 14
Supplementary Fig.12 | Temporal changes of cellular niches 15
Supplementary Fig.13 | Spatial pathway activity maps inferred with PROGENy 16
Supplementary Fig.14 | Spatial Hallmarks of Cancer enrichment scores 17
Supplementary Fig.15 | Cell cycle, SASP, and adaptive mutability gene set activity 18
Supplementary Fig.16 | Spatial activity of Hallmarks gene sets 1/2 19
Supplementary Fig.17 | Spatial activity of Hallmarks gene sets 2/2 20
Supplementary Fig.18 | CNV clusters and cellular composition 21
Supplementary Fig.19 | CNV signatures across scRNA-seq-defined cell types 22
Supplementary Fig.20 | DGEA of EMT and proliferating tumour niches 23
Supplementary Fig.21 | Cell-cell communication within tissue compartments 24
Supplementary Fig.22 | In situ single-cell composition of mammary tumours by IMC 25
Supplementary Fig.23 | Intracellular Pt isotope distribution 26
Supplementary Fig.24 | Intracellular Pt concentration in tumour cells 27
Supplementary Fig.25 | Neighborhood enrichment in IMC data 28
Supplementary Fig.26 | Cellular niches in the multimodal ST-IMC dataset 29
Supplementary Fig.27 | Feature importance in the MISTy model 30
Supplementary Fig.28 | Histopathological annotation and niche gene weights in human ST 31
Supplementary Fig.29 | DGEA of residual and proliferating tumour niches in humans 32
Supplementary Fig.30 | Survival stratification by EMT–proliferative signatures 33
Supplementary Fig.31 | Image tiles of highest-scoring capture spots for cellular niches 34
Supplementary Fig.32 | Source data for western blots 35

Supplementary Figures

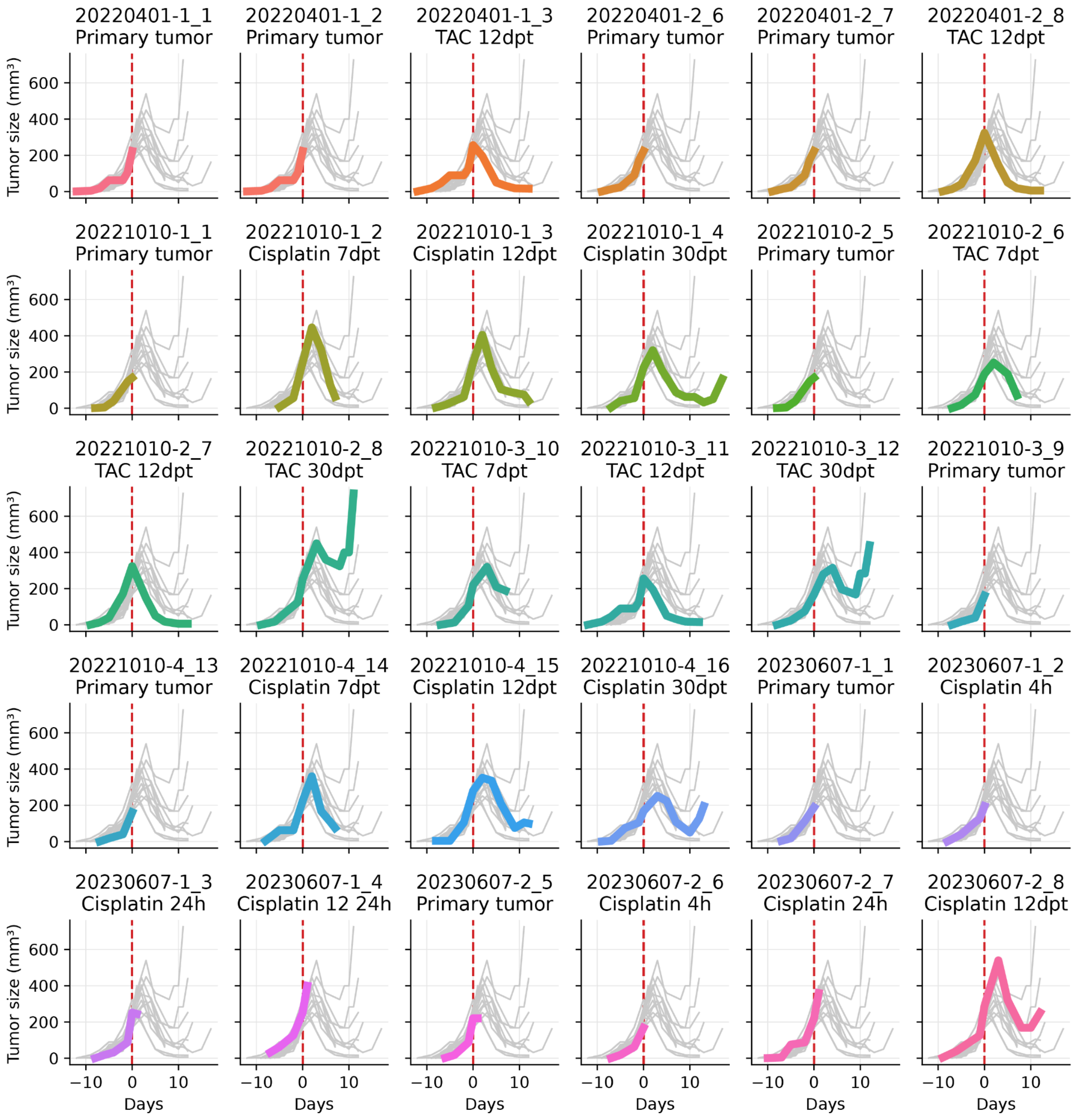

Supplementary Fig.1 | Tumour growth in the KB1P mouse model

Tumour growth curves of KB1P mammary tumours. Red dashed lines indicate chemotherapy treatments (cisplatin, TAC).

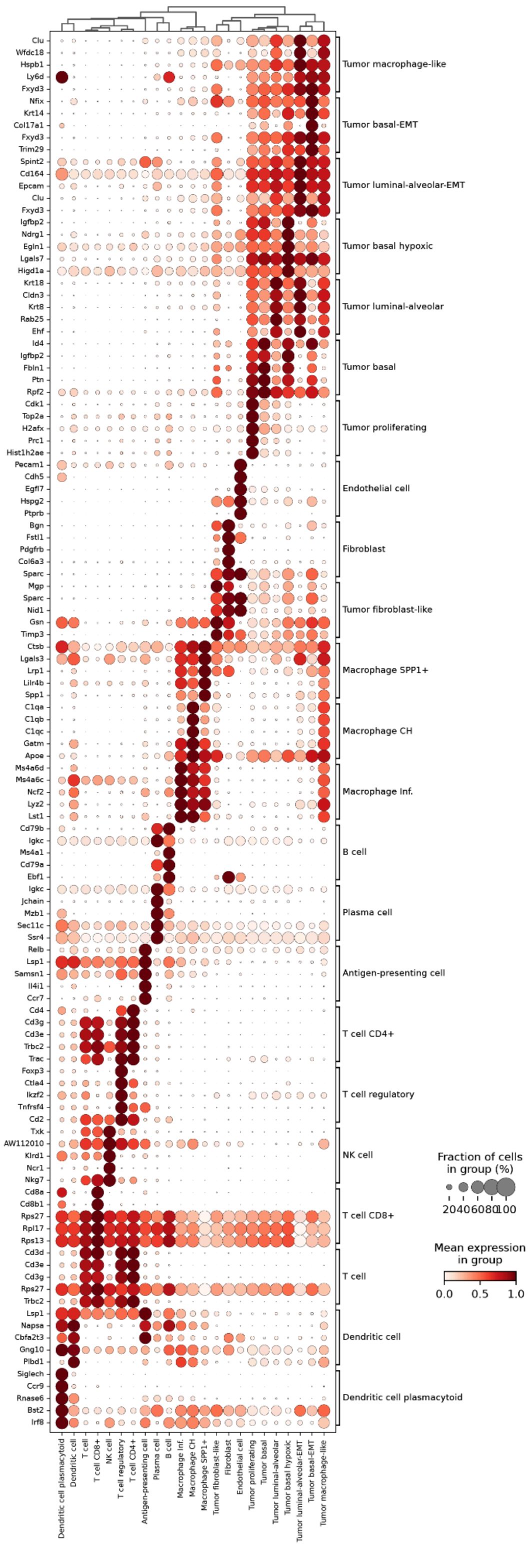

Supplementary Fig.2 | Cell-type marker genes in scRNA-seq

Dot plot showing the top five marker genes for each identified cell type, calculated using the Wilcoxon rank-sum test on scRNA-seq data (n_{Primary} = 3, n_{Residual} = 3, n_{Cell} = 11,566).

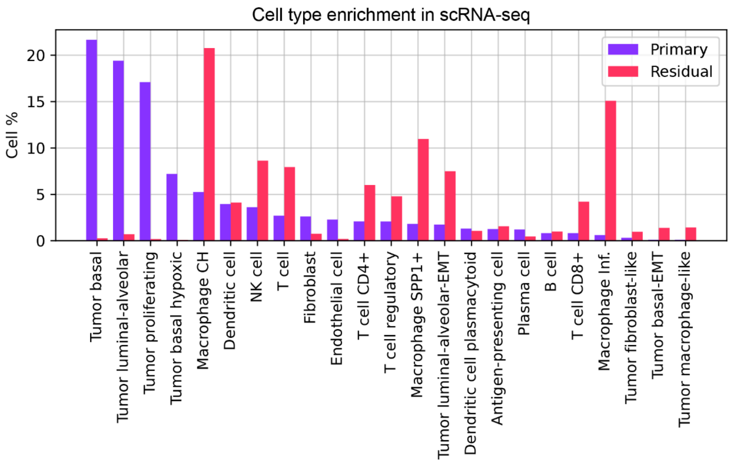

Supplementary Fig.3 | Cell-type composition in primary and residual tumours

Bar plot showing the fractional cell-type composition in primary and residual tumours ($n_{Primary}$ = 3, $n_{Residual}$ = 3, n_{Cell} = 11,566).

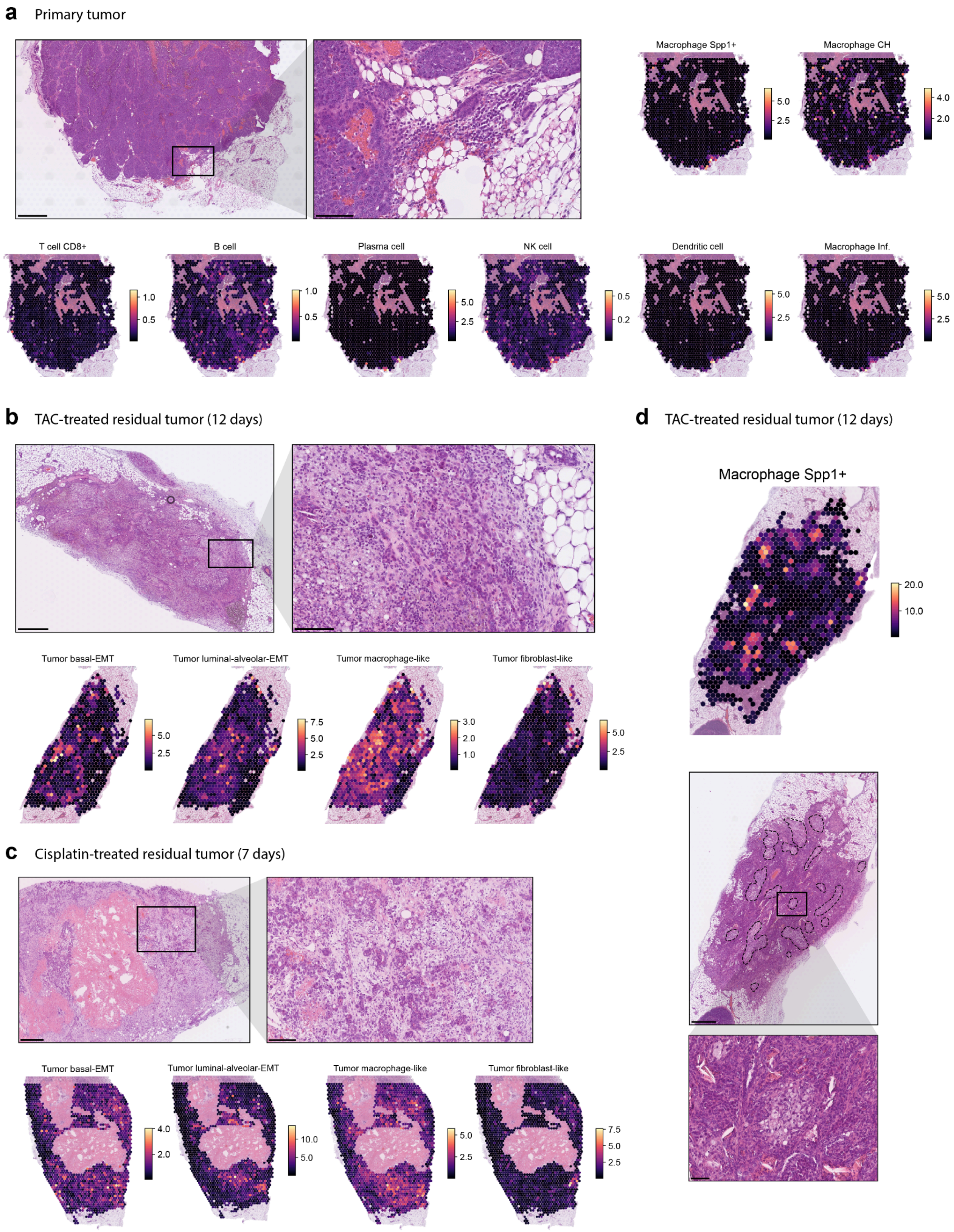

Supplementary Fig.4 | Spatiotemporal histopathological changes during MRD

a, Representative H&E images of a primary tumour sample showing mixed immune cell infiltration along the tumour margins, with the corresponding spatial localisation of the indicated cell types within the Visium tissue section (scale bars: left 500 µm, right 100 µm, n = 32, n_{Spot} = 65,304). **b,** Representative H&E images of a TAC-treated residual tumour with the corresponding spatial localisation of the indicated tumour cell types within the Visium tissue section. The epithelial tumour cells gradually lost cell-cell contact, became more elongated, and were increasingly difficult to identify within the reactive stroma (scale bar: left 500 µm, right 100 µm, n = 32, n_{Spot} = 65,304). **c,**

Representative H&E images of a cisplatin-treated residual tumour with the corresponding spatial localisation of the indicated tumour cell types within the Visium tissue section. Similar morphological changes were observed in the tumour tissue, albeit to a lesser extent. Epithelial clusters remained more defined (scale bar: left 500 µm, right 100 µm, n = 32, n_{Spot} = 65,304). **d,** Spatial localisation of *Spp1*+ macrophages within the Visium tissue section, alongside the corresponding H&E image in a TAC-treated residual tumour. Foamy *Spp1*+ macrophages are delineated in blue (scale bar: 500 µm) and depicted at higher magnification (lower panel). The spatial plots in panels a, b, and d depict cell type abundance data inferred with cell2location (n = 32, n_{Spot} = 65,304).

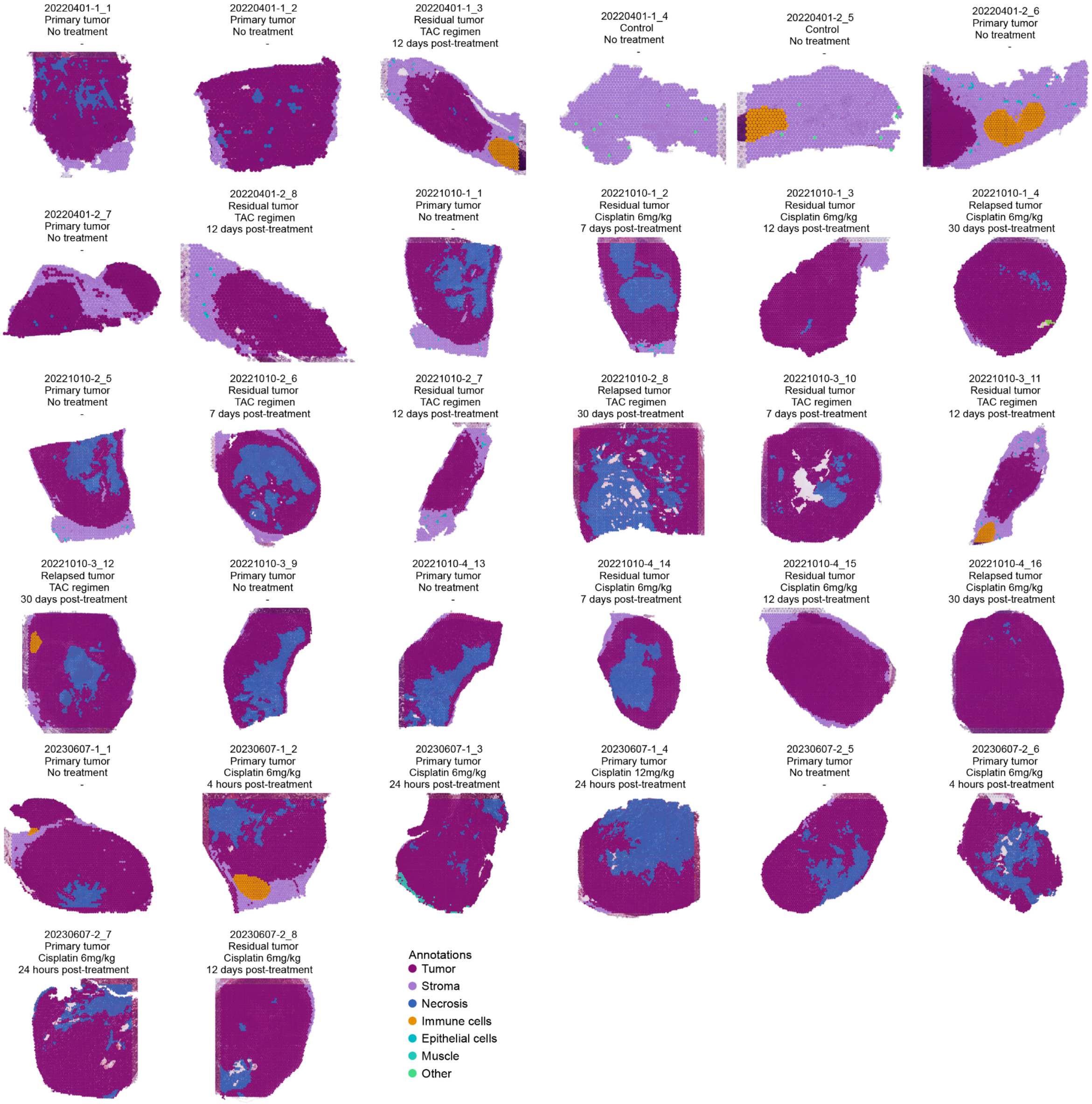

Supplementary Fig.5 | Histopathological annotations of capture spots

Labelling of capture spots based on histopathological annotations (n = 32, n_{Spot} = 65,304). For downstream analysis, only capture spots containing tumour tissue were considered. Categories include: Tumour (tumour tissue), Stroma (mammary fat pad), Necrosis (dead tissue), Immune cells (lymph node), Epithelial cells (mammary gland ducts), Muscle (muscle tissue), Other/ignore (undefined tissue).

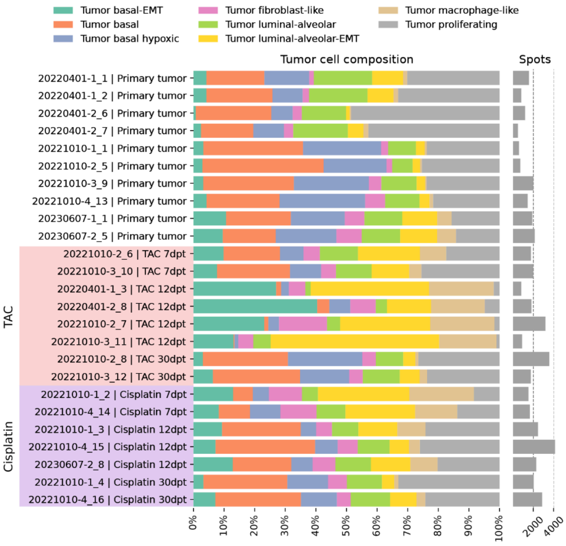

Supplementary Fig.6 | Sample-wise changes in tumour cell composition following treatment
Tumour cell composition of ST samples after cell-type deconvolution. Proliferating, basal, luminal–alveolar and basal hypoxic tumour cell fractions decrease in residual tumours, whereas EMT, macrophage-like and fibroblast-like tumour cell fractions increase, independent of treatment regimen. These changes revert in recurrent tumours (n = 25, n_{Spot} = 45,543).

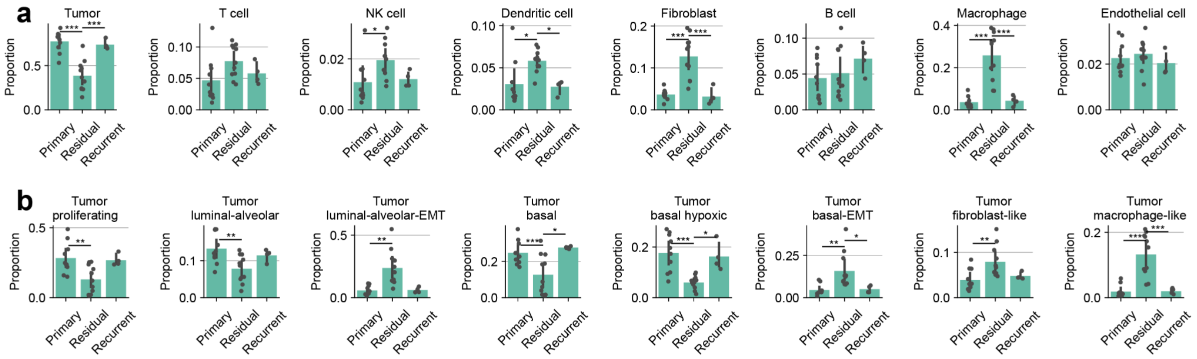

Supplementary Fig.7 | Changes in cell-type composition during tumour progression

Barplots showing the mean cell-type fraction across samples (black dots: individual ST samples, error bar: 95% CI, bar height: mean value, statistical significance was assessed using one-way ANOVA with Tukey's HSD post hoc test for pairwise comparisons: ***, $p < 0.001$). Sample sizes are $n_{\text{Primary}} = 8$, $n_{\text{Residual}} = 9$, and $n_{\text{Recurrent}} = 4$, where n values denote independent biological units (tumours).

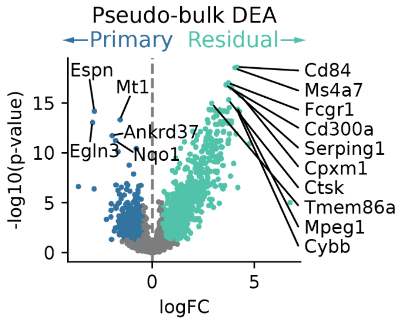

Supplementary Fig.8 | Pseudo-bulk DGEA of spatial transcriptomics

Volcano plot showing the DGEA of ST samples between primary and residual tumours (n_{Primary} = 10, n_{Residual} = 11, n_{Spot} = 33,862).

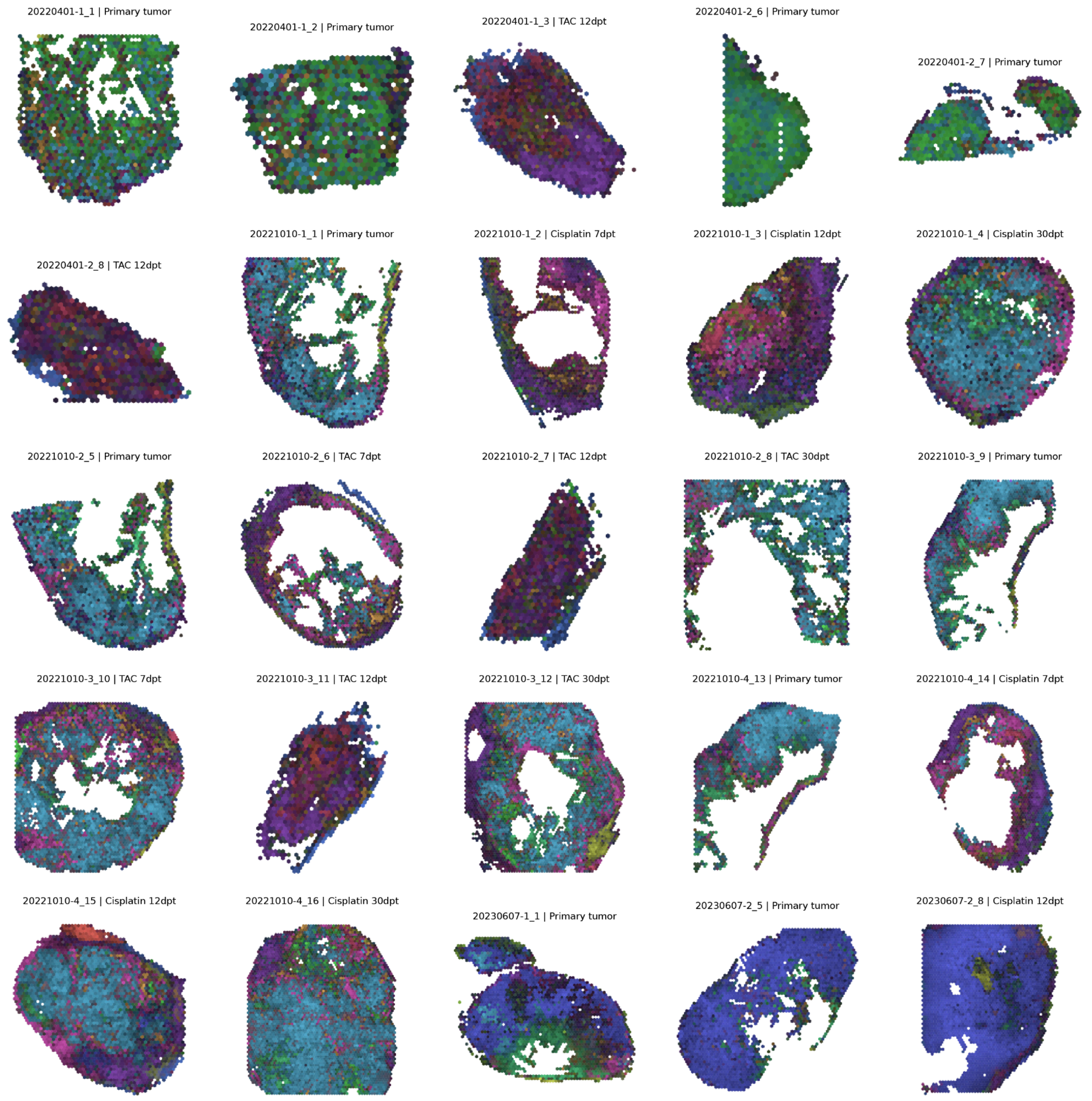

Supplementary Fig.9 | Cellular niche composition during MRD

MIP of molecular tissue compartments (cellular niches) identified by Chrysalis across all samples in the main ST dataset (n = 25, n_{Spot} = 45,543).

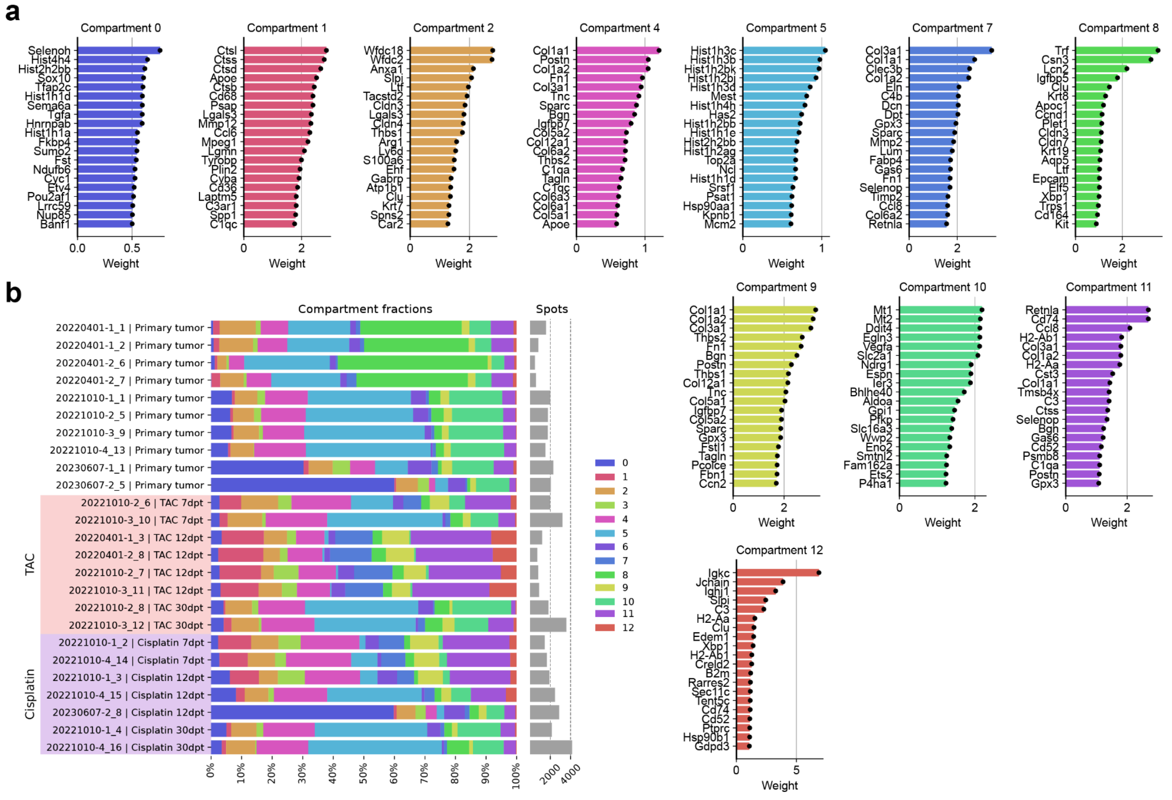

Supplementary Fig.10 | Cellular niches identified by Chrysalis

a, Top 20 genes with the highest weights for each cellular niche identified in the main ST dataset (n = 25, n_{Spot} = 45,543). **b,** Cellular niche composition across all Visium tissue samples (n = 25, n_{Spot} = 45,543).

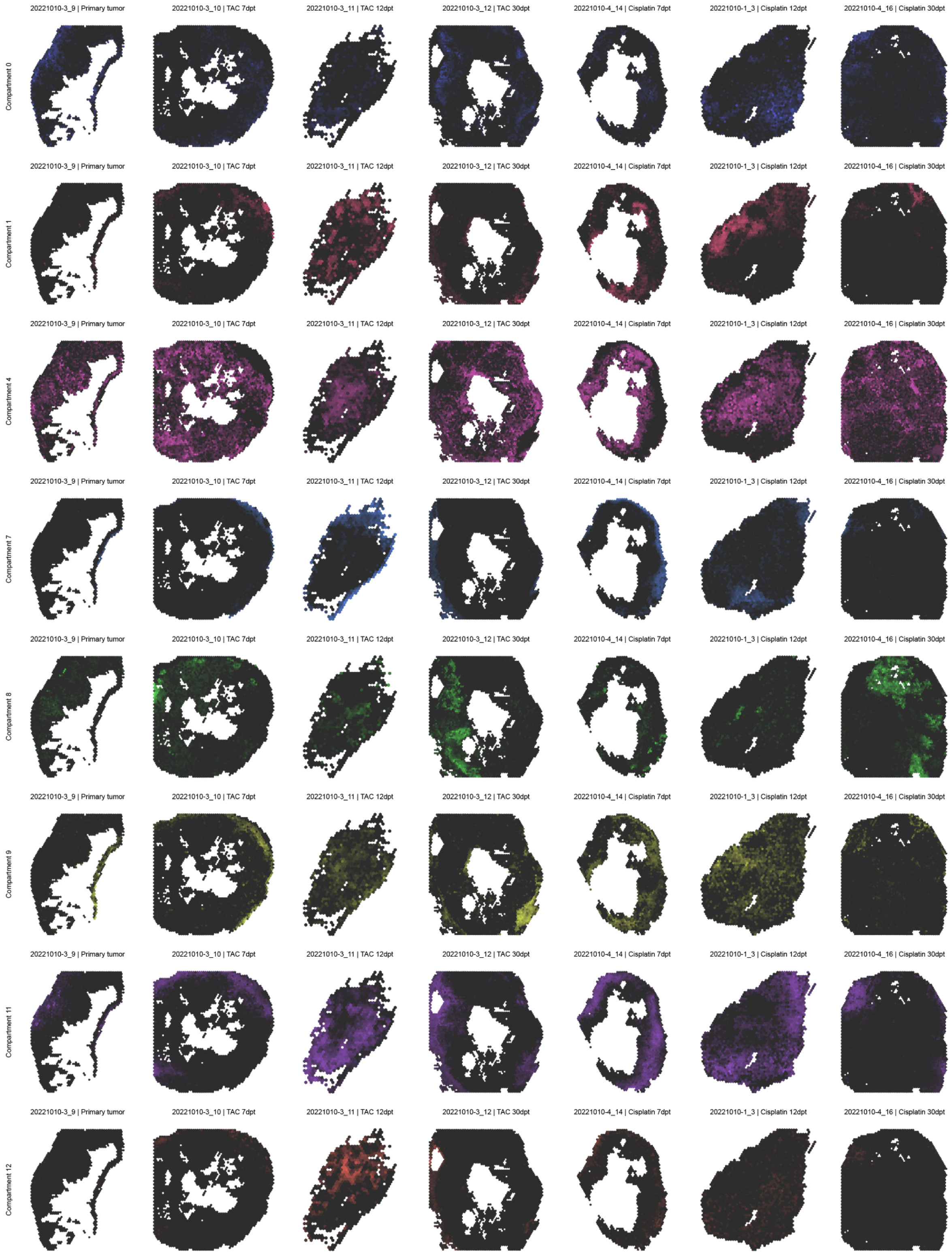

Supplementary Fig.11 | Spatiotemporal dynamics of cellular niches
Spatial maps of compartment scores for representative primary, residual and recurrent tumours following TAC or cisplatin treatment. Compartments 2, 5, and 10 are shown in **Fig.3d** (n = 25, n_{Spot} = 45,543).

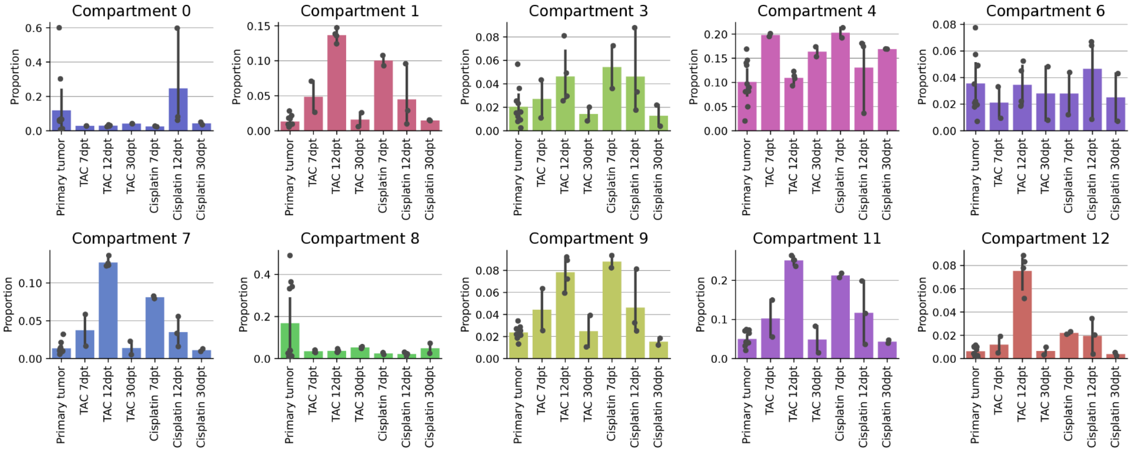

Supplementary Fig.12 | Temporal changes of cellular niches

Cellular niche proportions across tumour samples. Compartments 2, 5, and 10 are shown in **Fig.3e**. (bar height: mean value, black dots: individual ST samples, error bar: 95% CI; sample sizes: n_{Primary} = 8, $n_{\text{TAC 7 dpt}}$ = 2, $n_{\text{TAC 12 dpt}}$ = 2, $n_{\text{TAC 30 dpt}}$ = 2, $n_{\text{Cisplatin 7 dpt}}$ = 2, $n_{\text{Cisplatin 12 dpt}}$ = 3, $n_{\text{Cisplatin 30 dpt}}$ = 2, all n values denote independent biological units (tumours)).

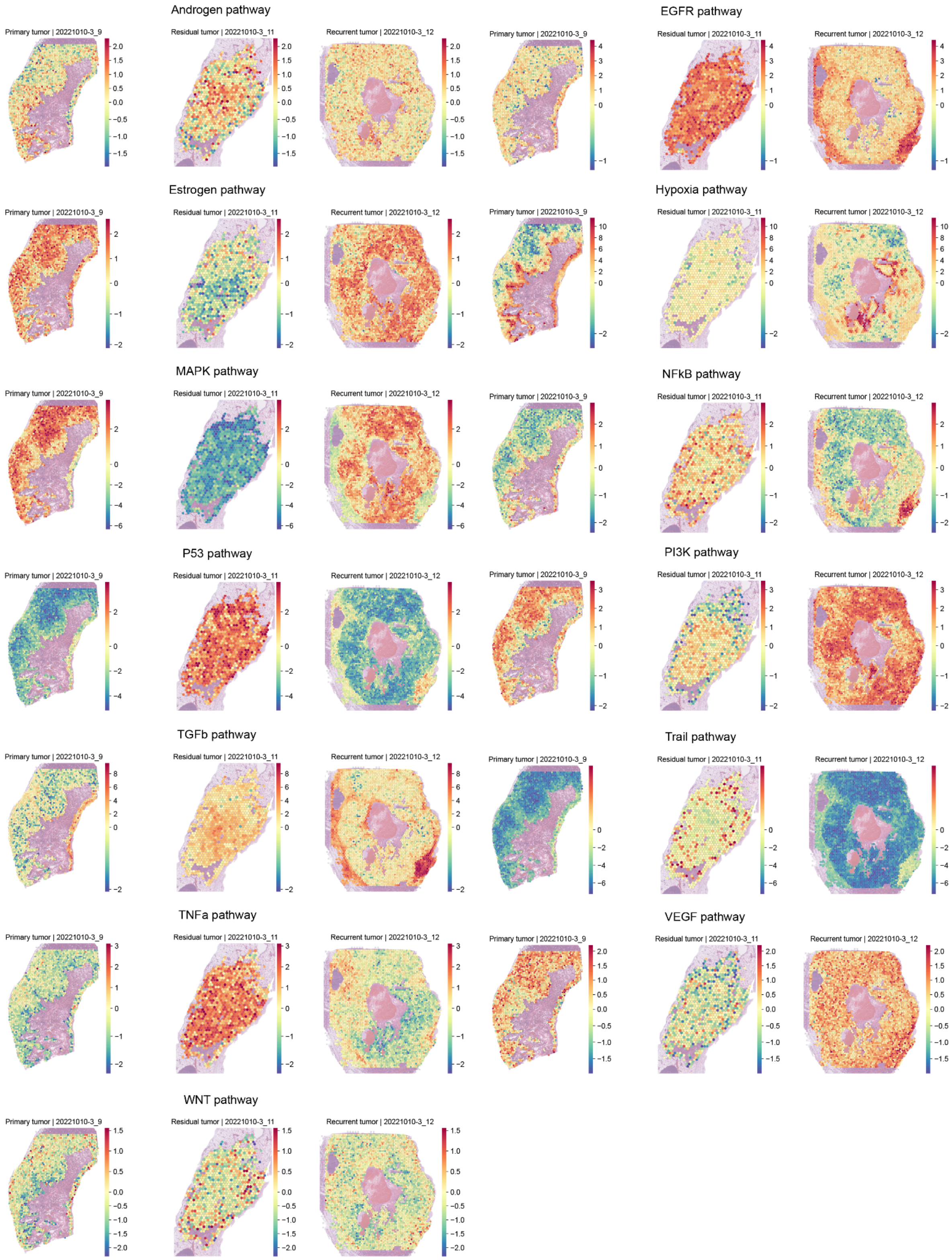

Supplementary Fig.13 | Spatial pathway activity maps inferred with PROGENy

Spatial maps of pathway activities inferred using PROGENy for representative samples (n = 25, n_{Spot} = 45,543).

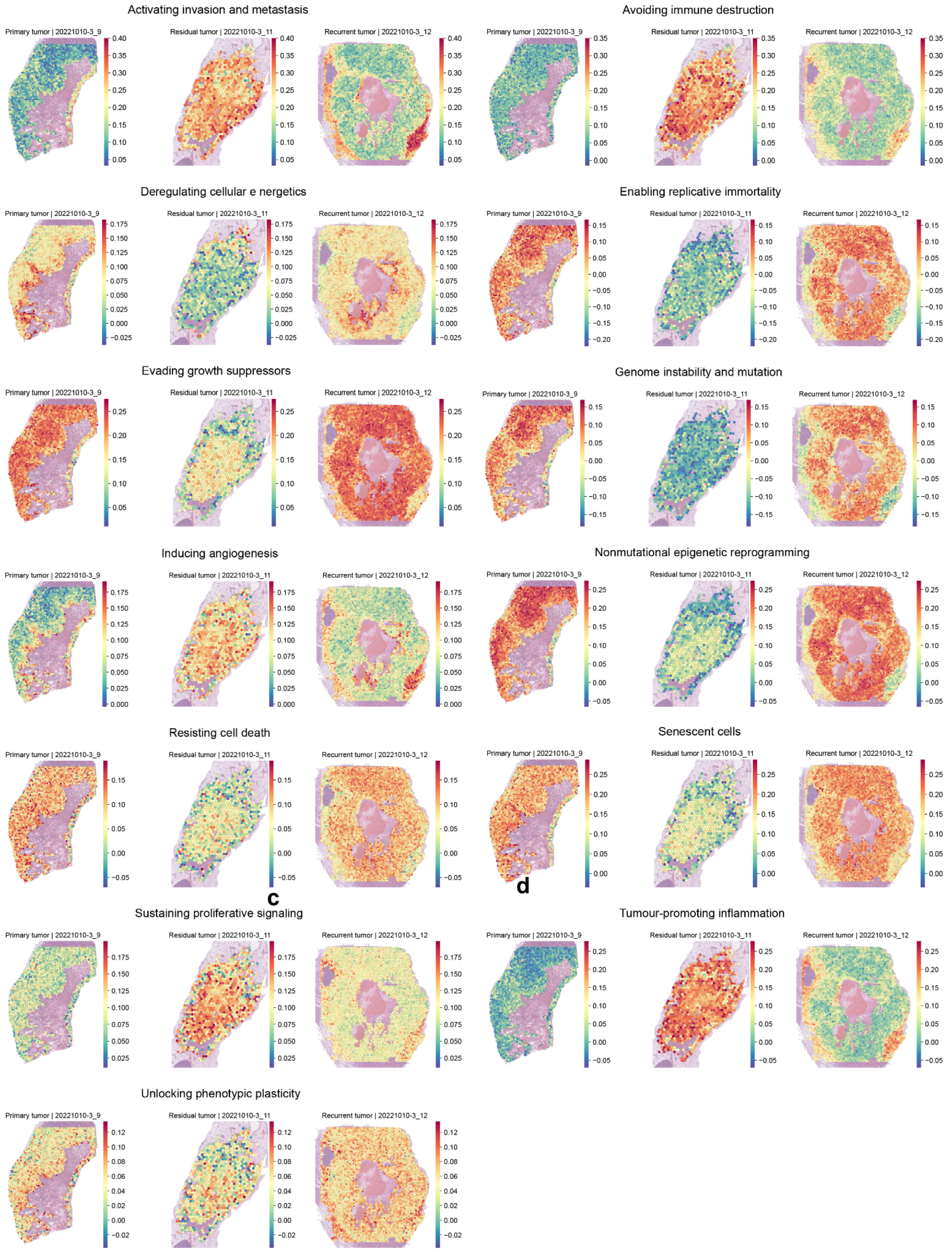

Supplementary Fig.14 | Spatial Hallmarks of Cancer enrichment scores

Spatial maps of Hallmarks of Cancer enrichment scores for representative samples (n = 25, n_{Spot} = 45,543).

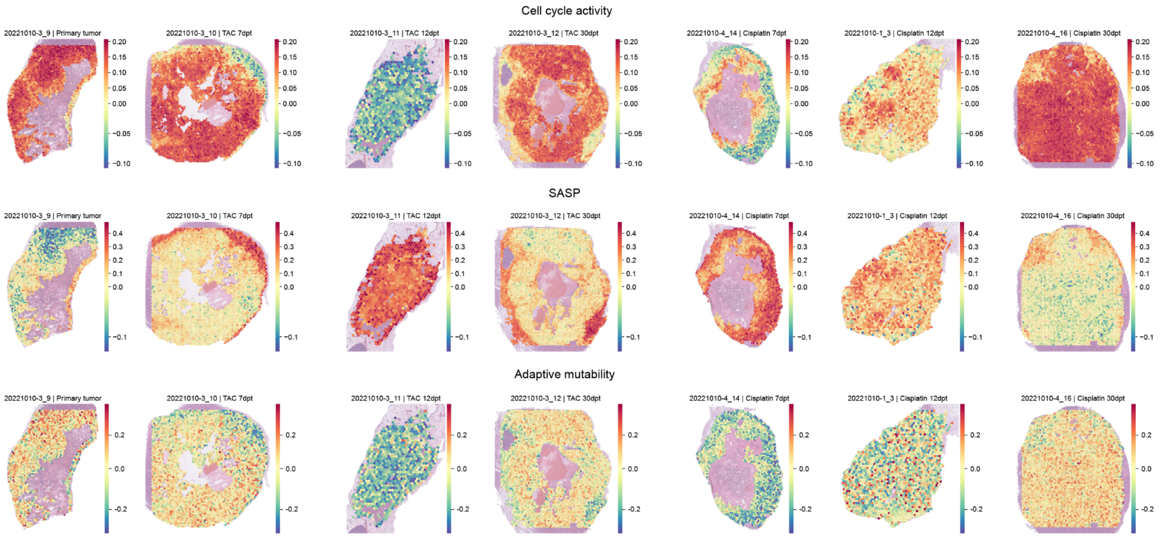

Supplementary Fig.15 | Cell cycle, SASP, and adaptive mutability gene set activity

Spatial maps of cell cycle, SASP and adaptive mutability gene set scores for representative samples (n = 25, n_{Spot} = 45,543).

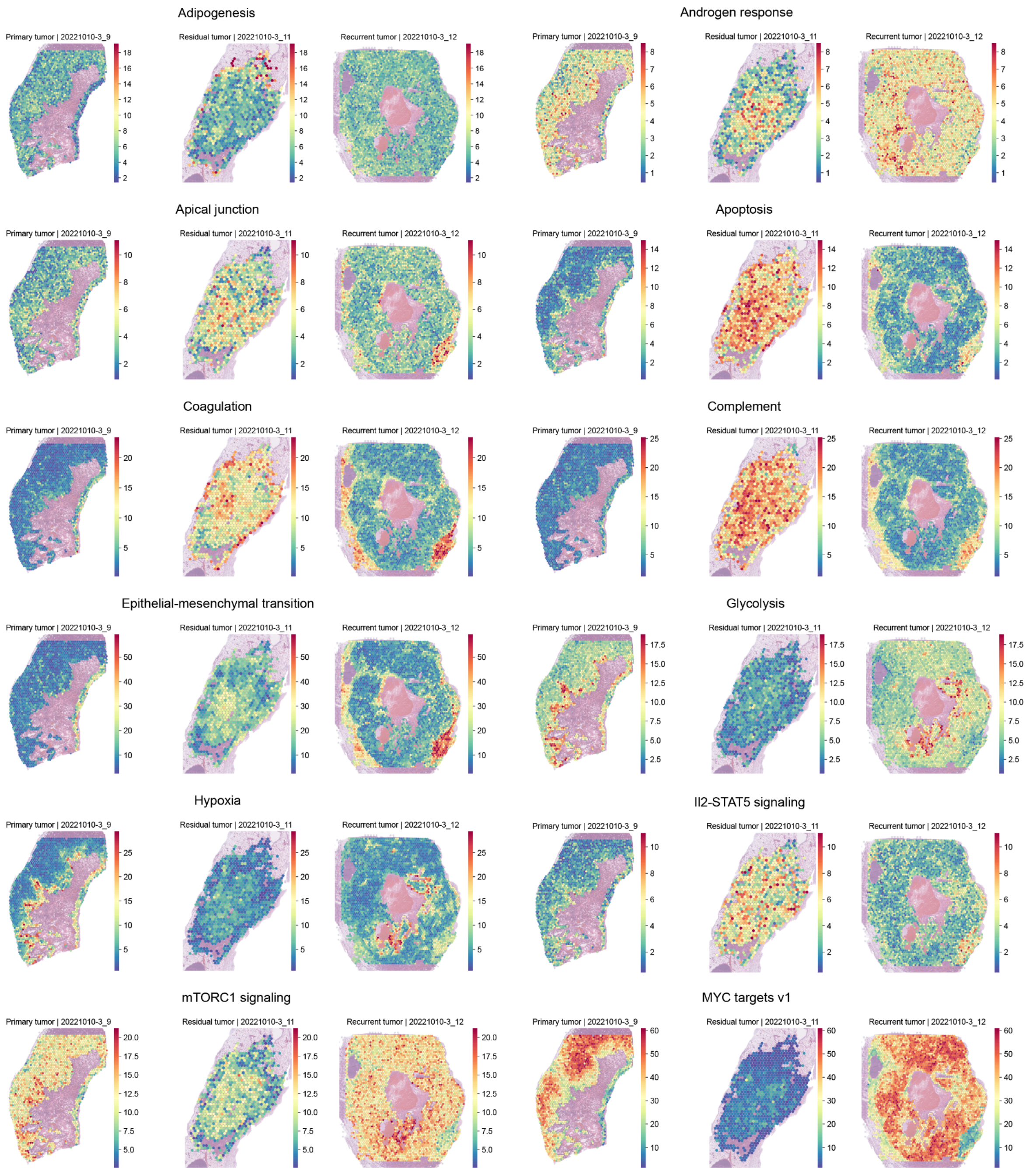

Supplementary Fig.16 | Spatial activity of Hallmarks gene sets 1/2

Spatial maps of significantly enriched Hallmarks gene sets shown for representative samples (n = 25, n_{Spot} = 45,543).

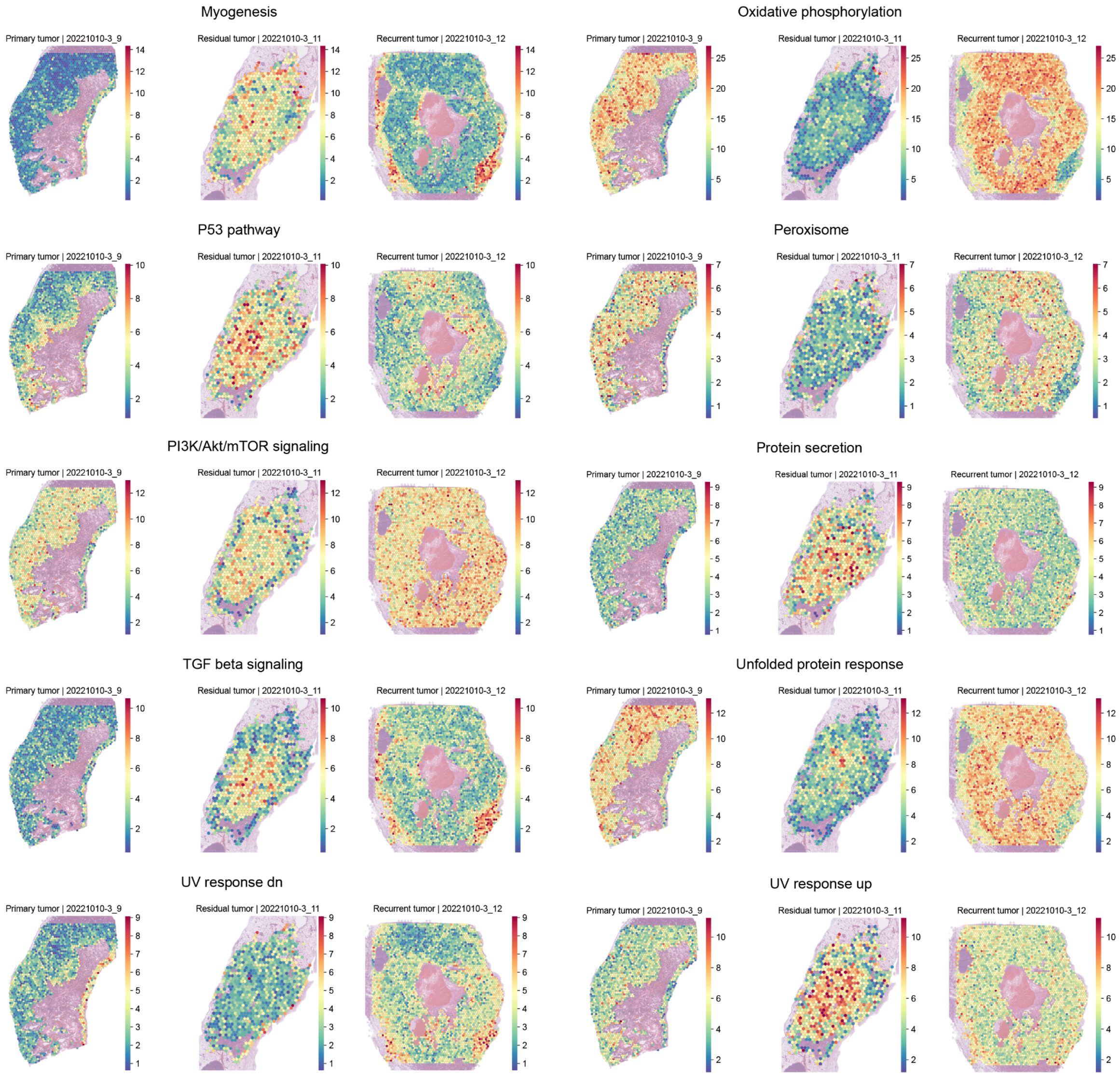

Supplementary Fig.17 | Spatial activity of Hallmarks gene sets 2/2

Spatial maps of significantly enriched Hallmarks gene sets shown for representative samples (n = 25, n_{Spot} = 45,543).

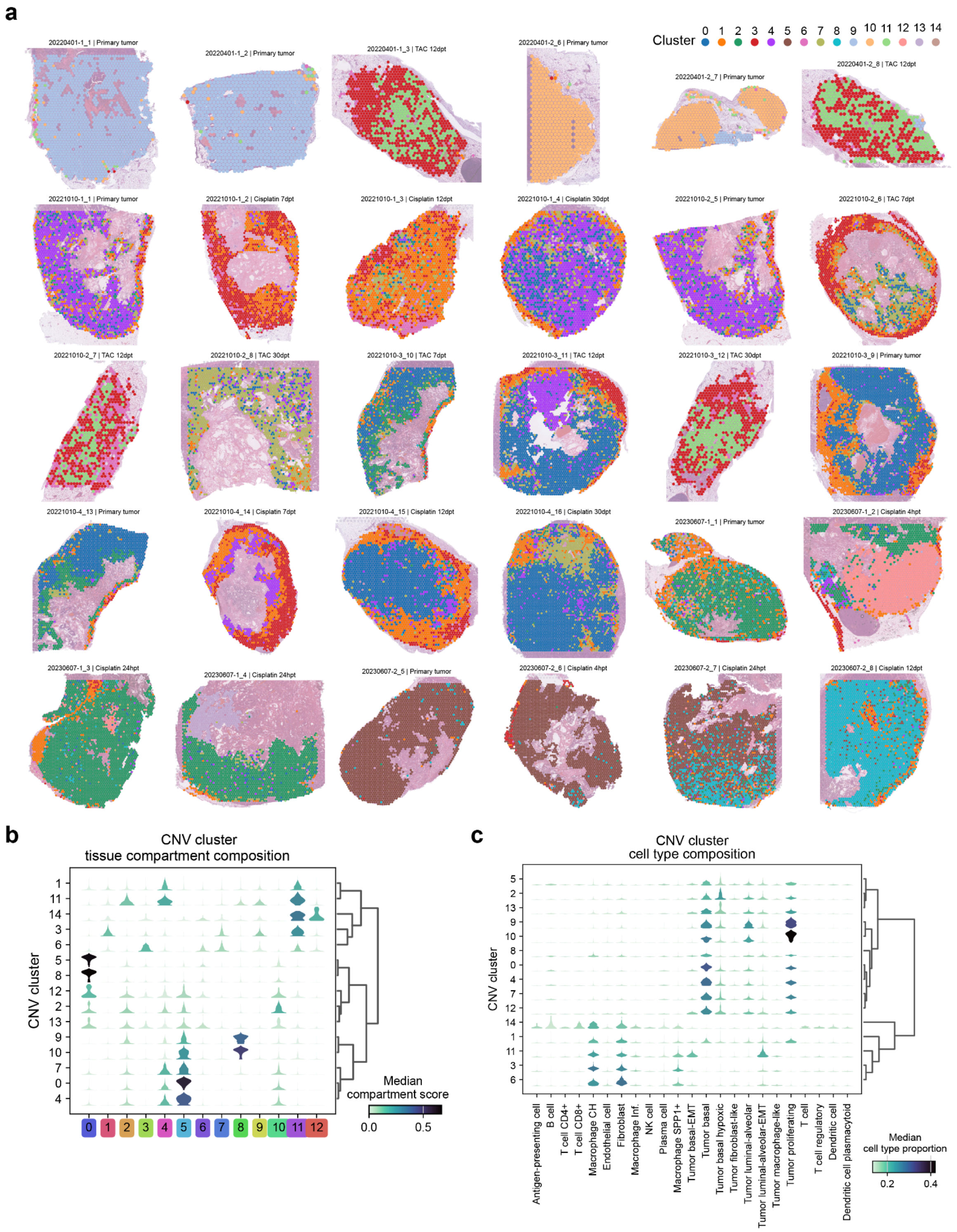

Supplementary Fig.18 | CNV clusters and cellular composition

a, Spatial distribution of CNV clusters across all Visium samples, showing that cluster distribution is more closely tied to their respective parental tumours than treatment conditions (n = 25, n_{Spot} = 45,543). **b,** Stacked violin plots depicting the cellular niche composition of each CNV cluster. **c,** Stacked violin plots showing the inferred cell type abundance composition of each CNV cluster.

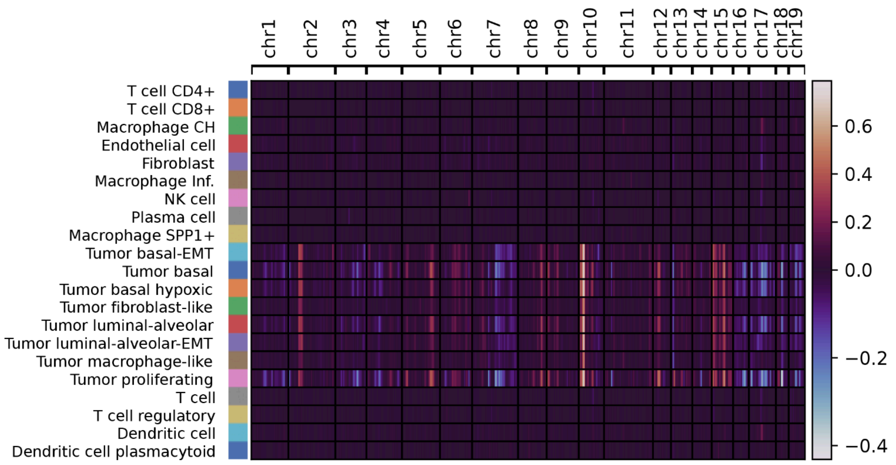

Supplementary Fig.19 | CNV signatures across scRNA-seq-defined cell types

CNV inference based on comparison of tumour and stromal cells. Heatmaps show chromosomal alterations ordered by cell type (n_{Primary} = 3, n_{Residual} = 3, n_{Cell} = 11,566).

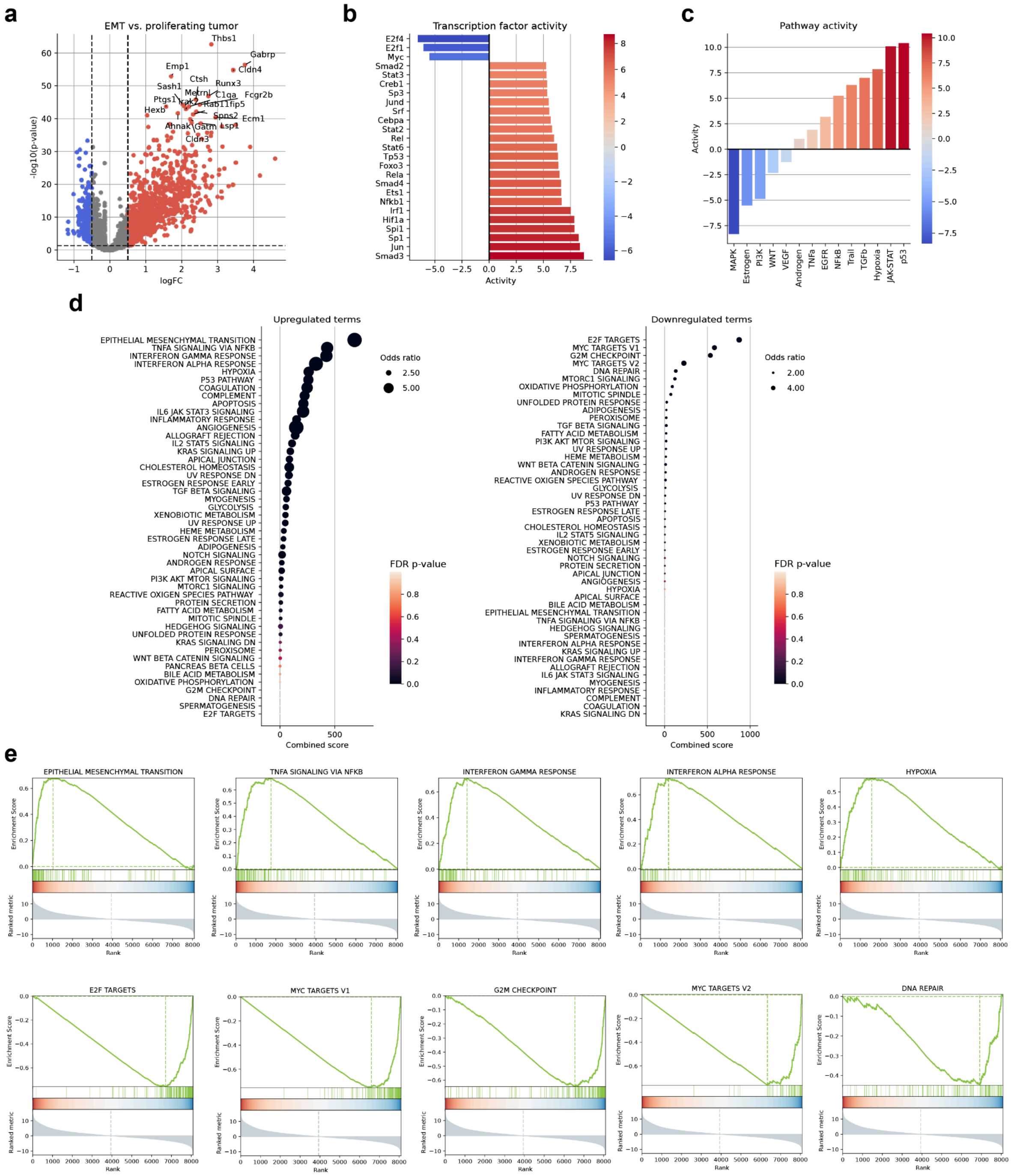

Supplementary Fig.20 | DGEA of EMT and proliferating tumour niches

Across all panels, differential gene expression and enrichment analyses were performed using n_{Primary} = 10, n_{Residual} = 11, $n_{\text{Spot-EMT}}$ = 564, $n_{\text{Spot-Proliferating}}$ = 940, n_{Gene} = 8,093. **a,** Pseudo-bulk DGEA results for capture spots with dominant EMT and proliferating niche scores. **b,** Transcription factor activity inferred from the DGEA results. **c,** Pathway activity inference based on differentially expressed genes. **d,** Dot plots showing upregulated and downregulated terms from the Hallmarks gene set collection. **e,** Enrichment plots of the top five upregulated and downregulated gene sets from the Hallmarks gene set collection.

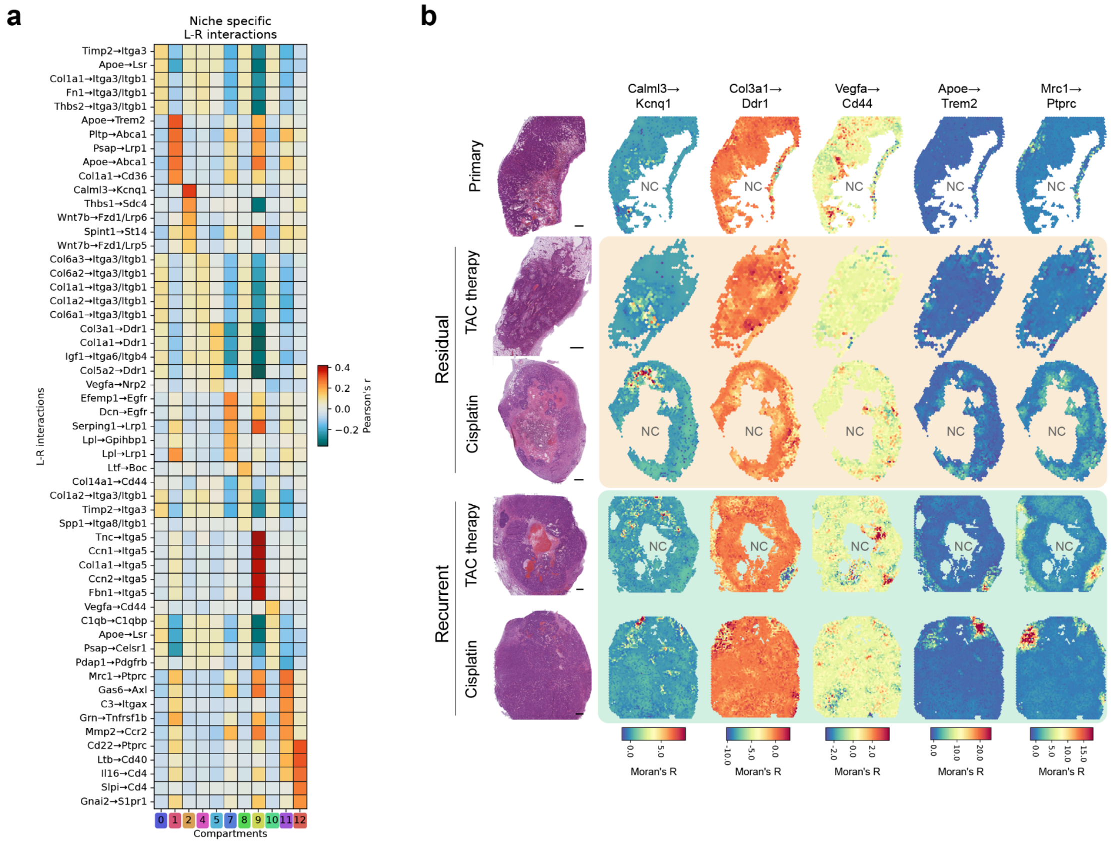

Supplementary Fig.21 | Cell-cell communication within tissue compartments

a, Heatmap of the top five most strongly correlating ligand–receptor interactions per compartment (n = 25, n_{Spot} = 45,543). **b,** Spatial maps of selected interactions shown in representative samples.

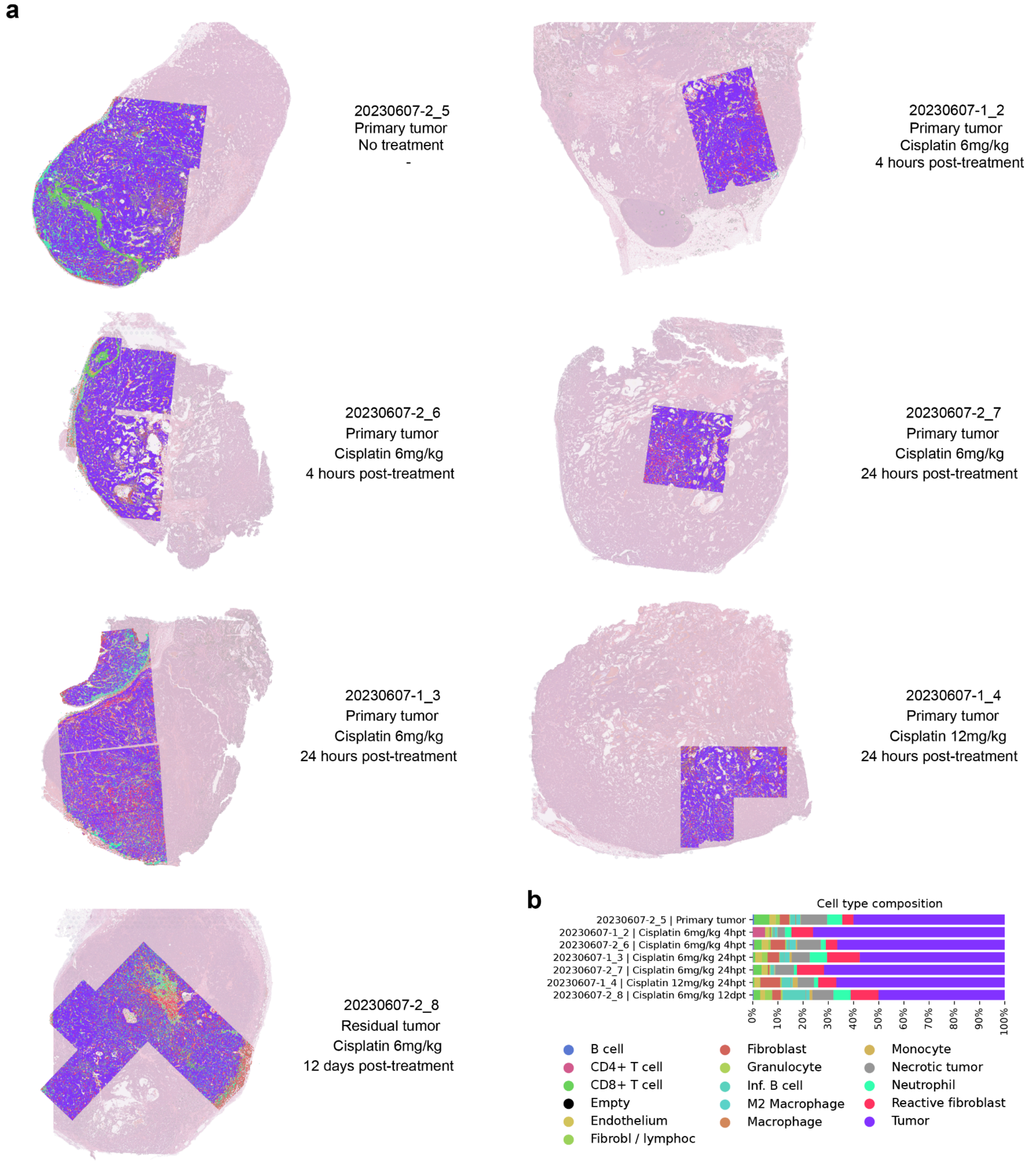

Supplementary Fig.22 | *In situ* single-cell composition of mammary tumours by IMC
a, Reconstructed in situ single-cell composition of tumour sections derived from IMC (n_{Animal} = 4, n_{ROI} = 37). **b,** Cell-type composition across tumours following cisplatin treatment (n_{Animal} = 4, n_{ROI} = 37).

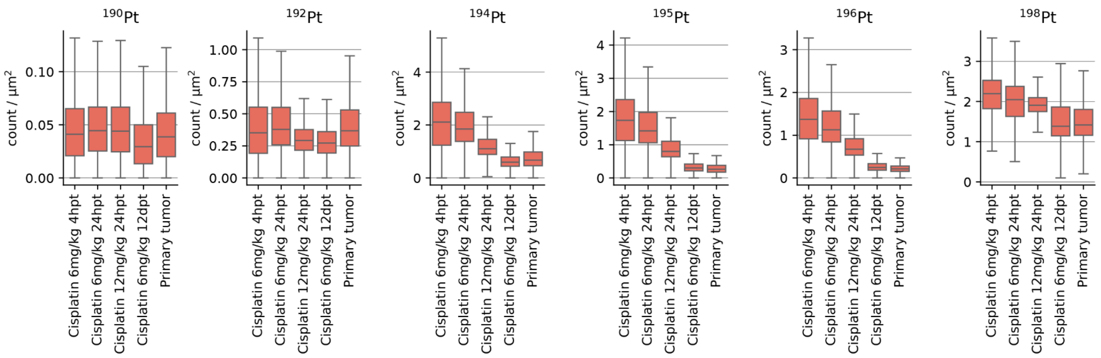

Supplementary Fig.23 | Intracellular Pt isotope distribution

Distribution of intracellular concentrations of six observationally stable Pt isotopes measured by IMC across conditions (centre line: median, box limits: upper and lower quartiles, whiskers: 1.5x interquartile range, n_{Animal} = 6, n_{ROI} = 50).

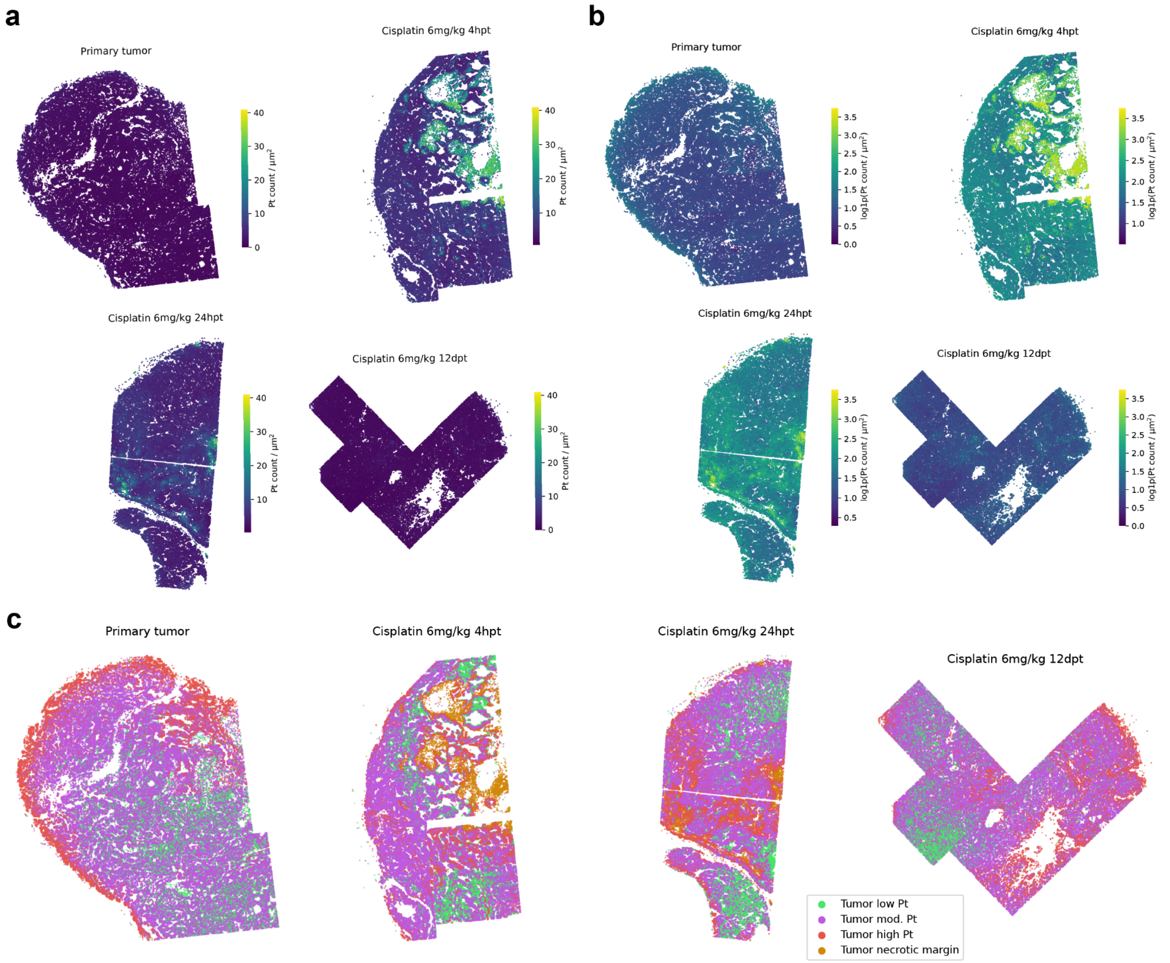

Supplementary Fig.24 | Intracellular Pt concentration in tumour cells

a, Raw intracellular Pt concentrations measured by IMC across four conditions (n_{Animal} = 7, n_{ROI} = 53). **b,** Log-transformed intracellular Pt concentrations. **c,** Classification of tumour cells based on intracellular Pt content (n_{Animal} = 7, n_{ROI} = 53).

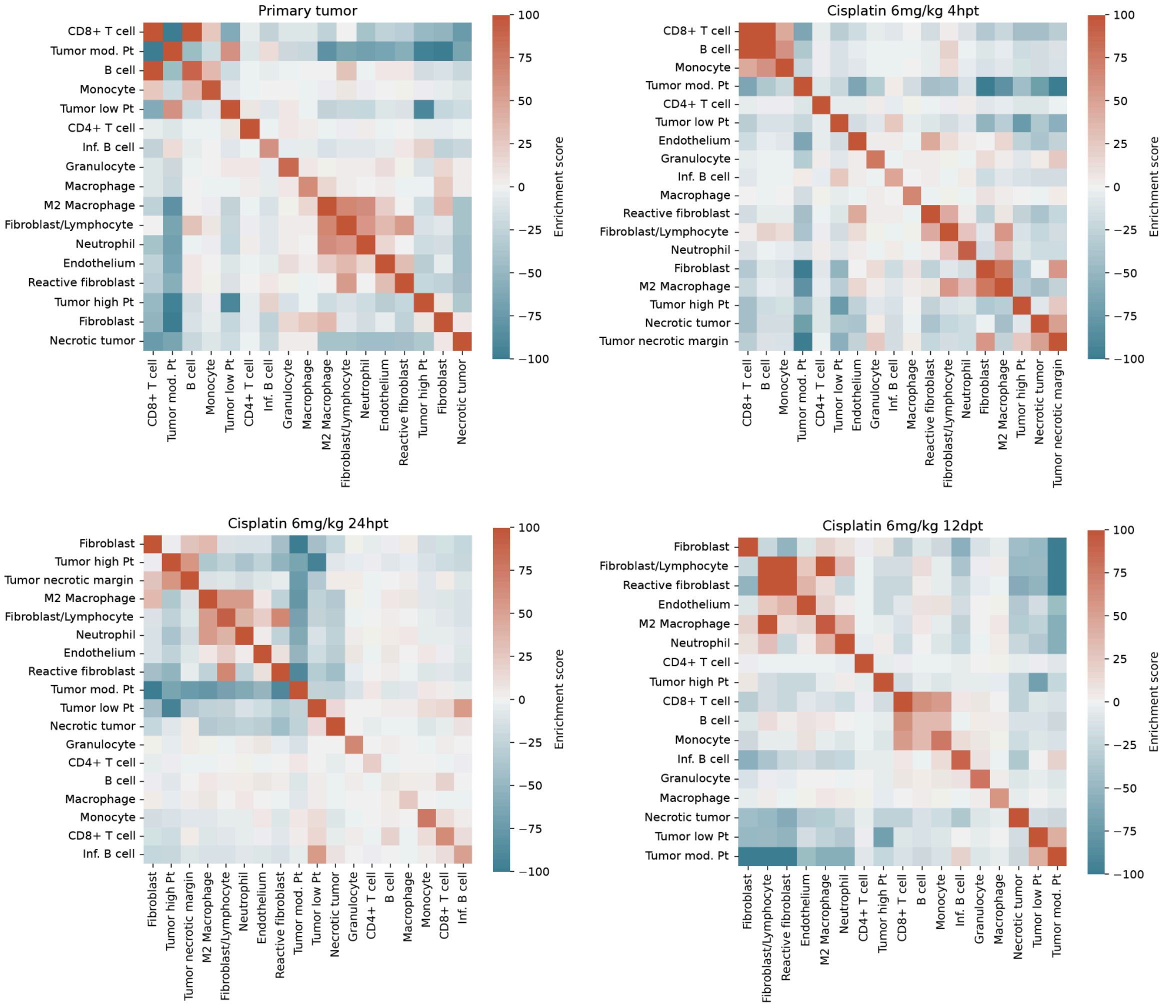

Supplementary Fig.25 | Neighborhood enrichment in IMC data

Neighbourhood enrichment scores of cell types reconstructed from IMC across four conditions (n_{Animal} = 4, n_{ROI} = 44).

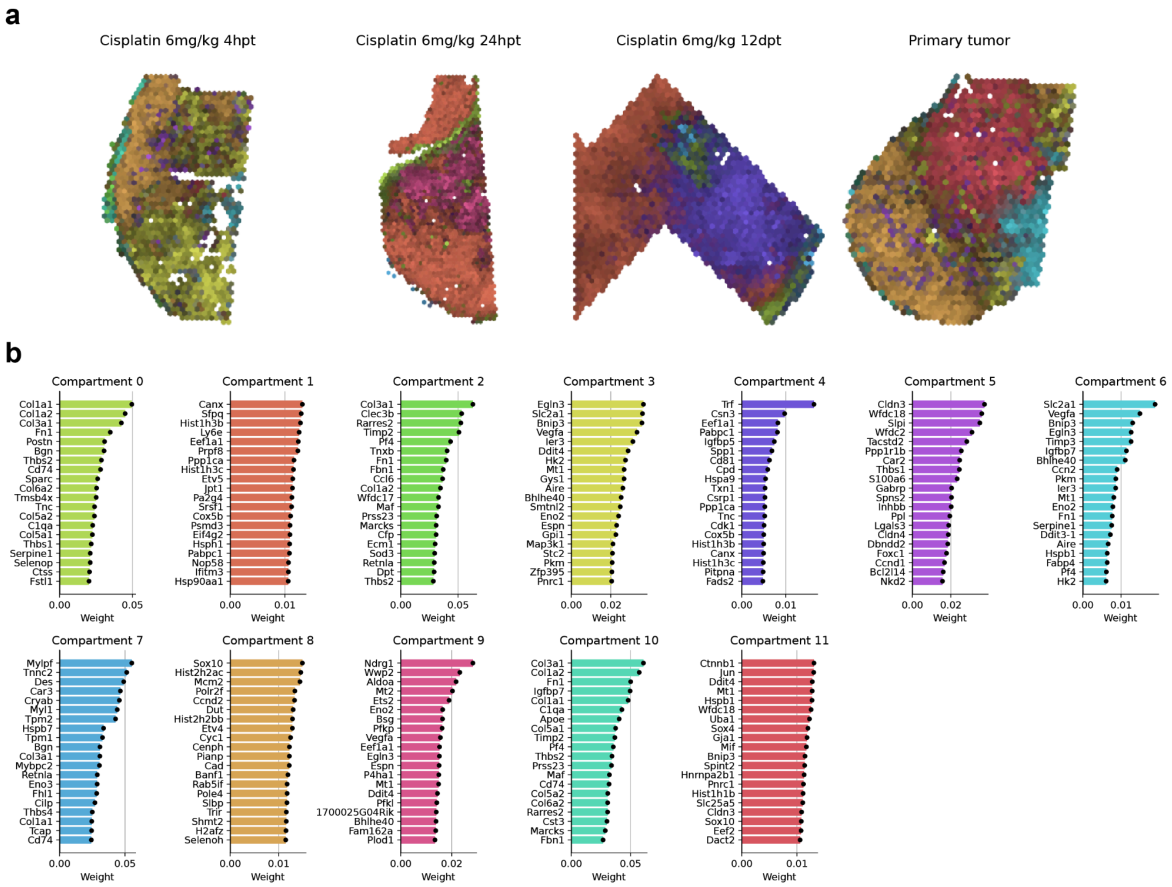

Supplementary Fig.26 | Cellular niches in the multimodal ST-IMC dataset

a, MIP of cellular niches identified by Chrysalis across the Visium component of the multimodal ST-IMC dataset (n = 4). **b,** Top 20 genes with the highest weights for each niche (n = 4).

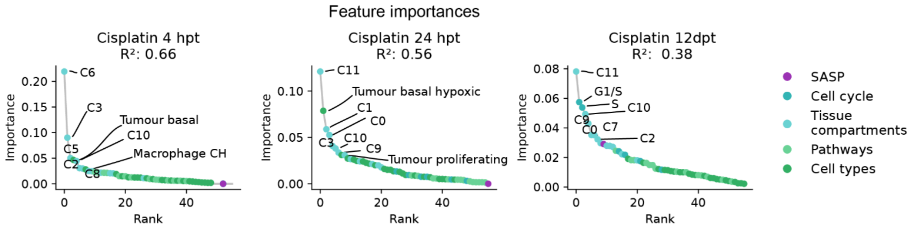

Supplementary Fig.27 | Feature importance in the MISTy model

Ranked importance of transcriptional features contributing to intracellular Pt content (*n* = 4).

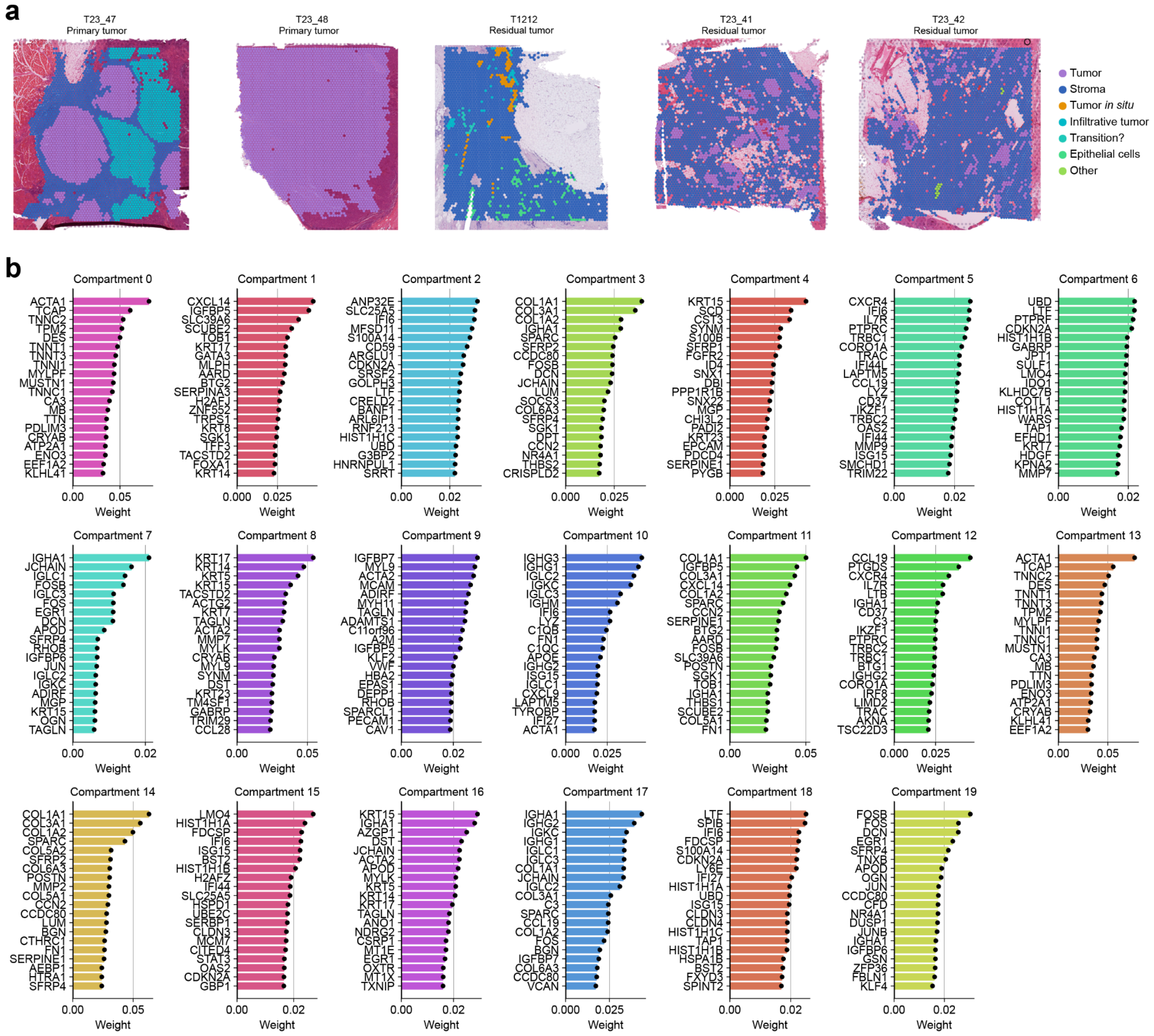

Supplementary Fig.28 | Histopathological annotation and niche gene weights in human ST
a, Labelling of capture spots based on histopathological annotations. In primary tumours, tumour tissue was annotated based on invasiveness: Tumour (typical epithelial pattern), Infiltrative tumour (cells lose cell-cell contact and intermingle with immune cells and stroma). In residual tumours, epithelial tissue was annotated based on morphology: Epithelial cells (resembling healthy ducts with no signs of malignancy), Tumour *in situ* (visually malignant but well demarcated), Infiltrative tumour (visually malignant and invading surrounding tissue), Transition (cells transitioning from normal to malignant epithelial cells), Other (undefined tissue, n_{Primary} = 2, n_{Residual} = 3, n_{Spot} = 17,340). **b,** Top 20 genes with the highest weights for each cellular niche identified in the human ST dataset (n = 5).

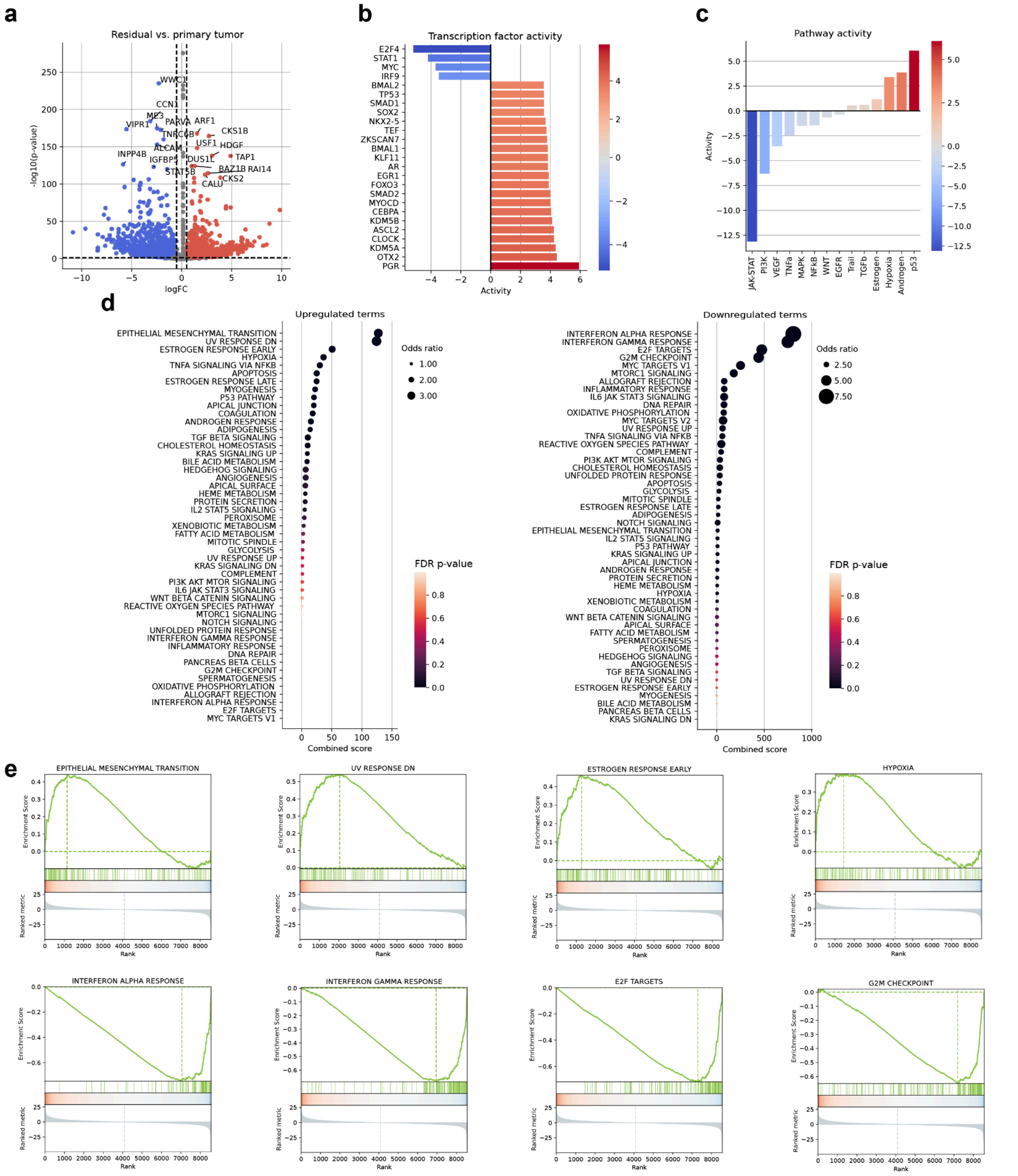

Supplementary Fig.29 | DGEA of residual and proliferating tumour niches in humans

Across all panels, differential gene expression and enrichment analyses were performed using $n_{Primary}$ = 2, $n_{Residual}$ = 3, n_{Spot} = 17,340, n_{Gene} = 10,525. **a,** Pseudo-bulk DGEA results for capture spots with dominant EMT and proliferating niche scores. **b,** Transcription factor activity inferred from the DGEA results. c, Pathway activity inference based on differentially expressed genes. **d,** Dot plots showing upregulated and downregulated terms from the Hallmarks gene set collection. **e,** Enrichment plots of the top five upregulated and downregulated gene sets from the Hallmarks gene set collection.

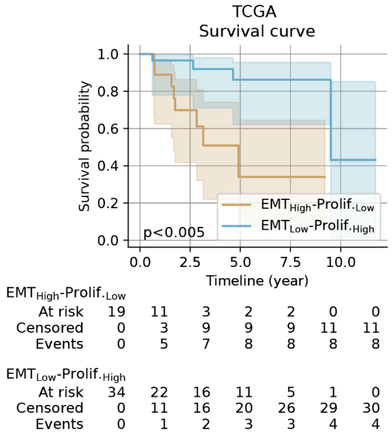

Supplementary Fig.30 | Survival stratification by EMT–proliferative signatures

Kaplan–Meier survival curves of TNBC patients (n = 53) from the TCGA cohort, stratified by EMT and proliferative signature expression (log-rank test $p < 0.005$, solid lines represent the cumulative survival probability, shaded areas denote the 95% confidence interval).

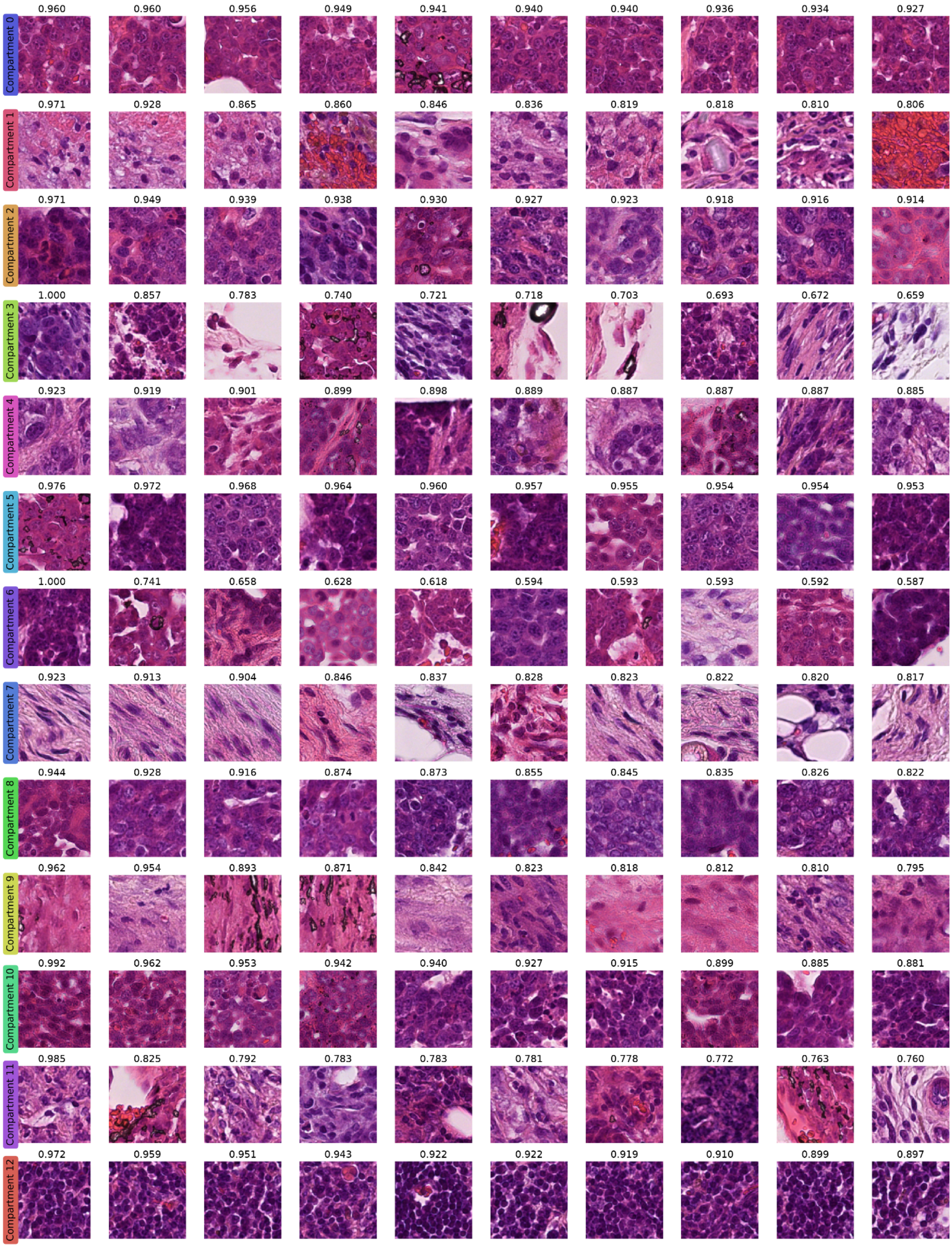

Supplementary Fig.31 | Image tiles of highest-scoring capture spots for cellular niches
Image tiles (55 × 55 µm) of the 10 highest scoring capture spots for each cellular niche in the main ST dataset (n = 25, n_{Spot} = 45,543).

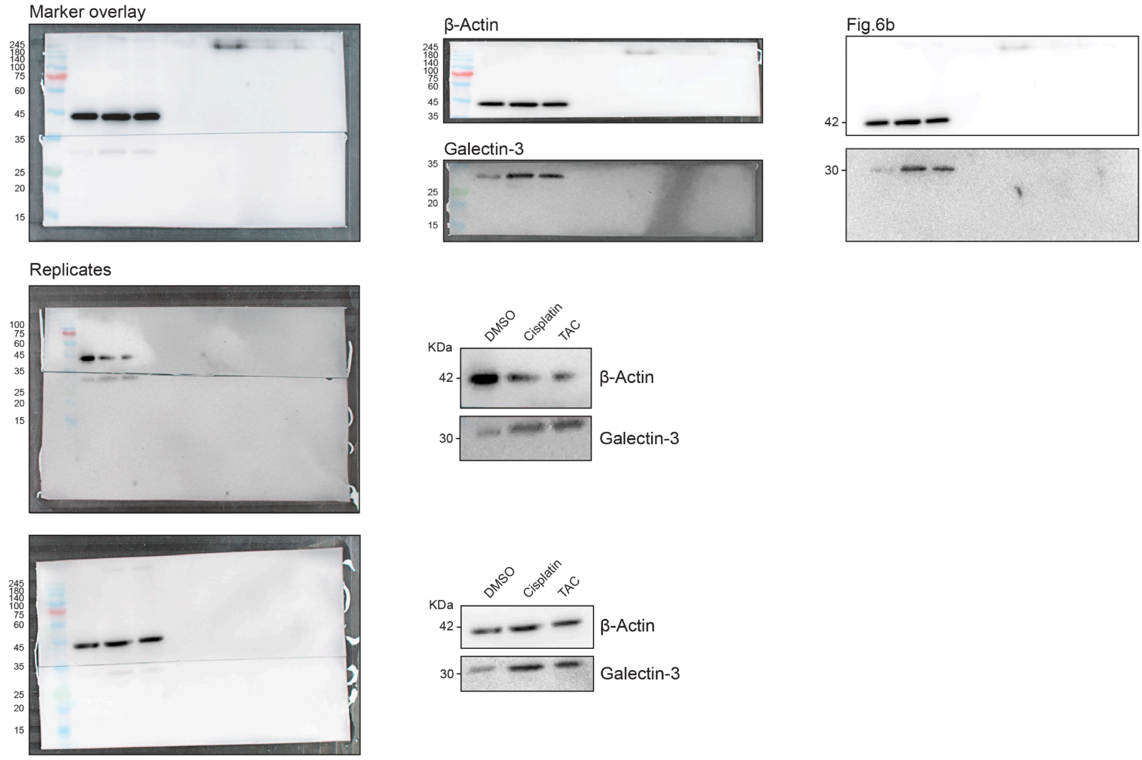

Supplementary Fig.32 | Source data for western blots
Uncropped western blot source data and replicates.
