# Supplementary material for: Spatiotemporal organisation of residual disease in mouse and human BRCA1-deficient mammary tumours and breast cancer
Source: Nat Commun. 2026 Jun 11;17:7456. doi: 10.1038/s41467-026-74125-6 (PMC13408813; doi:10.1038/s41467-026-74125-6)
Supplement: Supplementary file 2 — Description of Additional Supplementary Information [file 41467_2026_74125_MOESM2_ESM.pdf]

Description of Additional Supplementary Files

File name: Supplementary Data 1 - Mouse residual vs. primary DGEA (SC)
Description: Pseudo-bulk (single-cell) differential gene expression analysis results for mouse mammary tumour cells derived from residual and primary tumours.

File name: Supplementary Data 2 - Cell type fractions inferred using cell2location
Description: Spatial maps of inferred cell type abundances.

File name: Supplementary Data 3 - Mouse residual vs. primary DGEA (ST)
Description: Pseudo-bulk (spatial omics) differential gene expression analysis results for mouse mammary tumour cells derived from residual and primary tumours.

File name: Supplementary Data 4 - Mouse cellular niche weights
Description: Weights of spatially variable genes for cellular niches identified using Chrysalis in the primary mouse spatial omics dataset.

File name: Supplementary Data 5 - Mouse cellular niche gene sets
Description: Gene sets identified based on the weight distribution of spatially variable genes (mean + 2 SD) across cellular niches identified using Chrysalis in the primary mouse spatial omics dataset.

File name: Supplementary Data 6 - Cell-cell colocalization clusters
Description: Hierarchical clustering results describing cell-cell colocalization clusters across experimental conditions.

File name: Supplementary Data 7 - IMC channels
Description: Mapping of IMC isotope channels to the antibodies used for identifying cell types and biological processes.

File name: Supplementary Data 8 - Mouse cellular niche weights post-Pt
Description: Weights of spatially variable genes for cellular niches identified using Chrysalis in the multimodal (IMC-ST) mouse spatial omics dataset.

File name: Supplementary Data 9 - Human residual vs. primary DGEA (ST)
Description: Pseudo-bulk (spatial omics) differential gene expression analysis results for human mammary tumour cells derived from residual and primary tumours.

File name: Supplementary Data 10 - Human cellular niche weights
Description: Weights of spatially variable genes for cellular niches identified using Chrysalis in the human spatial omics dataset.

File name: Supplementary Data 11 - Small-molecule inhibitors for EMT signature genes
Description: Small-molecule inhibitors queried from PubChem for genes included in the EMT signature.

File name: Supplementary Data 12 - Mouse scRNA-seq metadata
Description: Metadata providing sample-specific details on treatment and data preprocessing for all mouse scRNA-seq samples.

File name: Supplementary Data 13 - Mouse ST metadata
Description: Metadata providing sample-specific details on treatment and data preprocessing for all mouse Visium samples.

File name: Supplementary Data 14 - Human ST metadata
Description: Metadata providing sample-specific details on treatment and data preprocessing for human Visium samples.

File name: Supplementary Data 15 - IMC metadata
Description: Metadata describing sample-specific details and pairing between IMC and Visium samples.
