# Supplementary material for: Spatiotemporal organisation of residual disease in mouse and human BRCA1-deficient mammary tumours and breast cancer
Source: Nat Commun. 2026 Jun 11;17:7456. doi: 10.1038/s41467-026-74125-6 (PMC13408813; doi:10.1038/s41467-026-74125-6)

Antigen-presenting cell

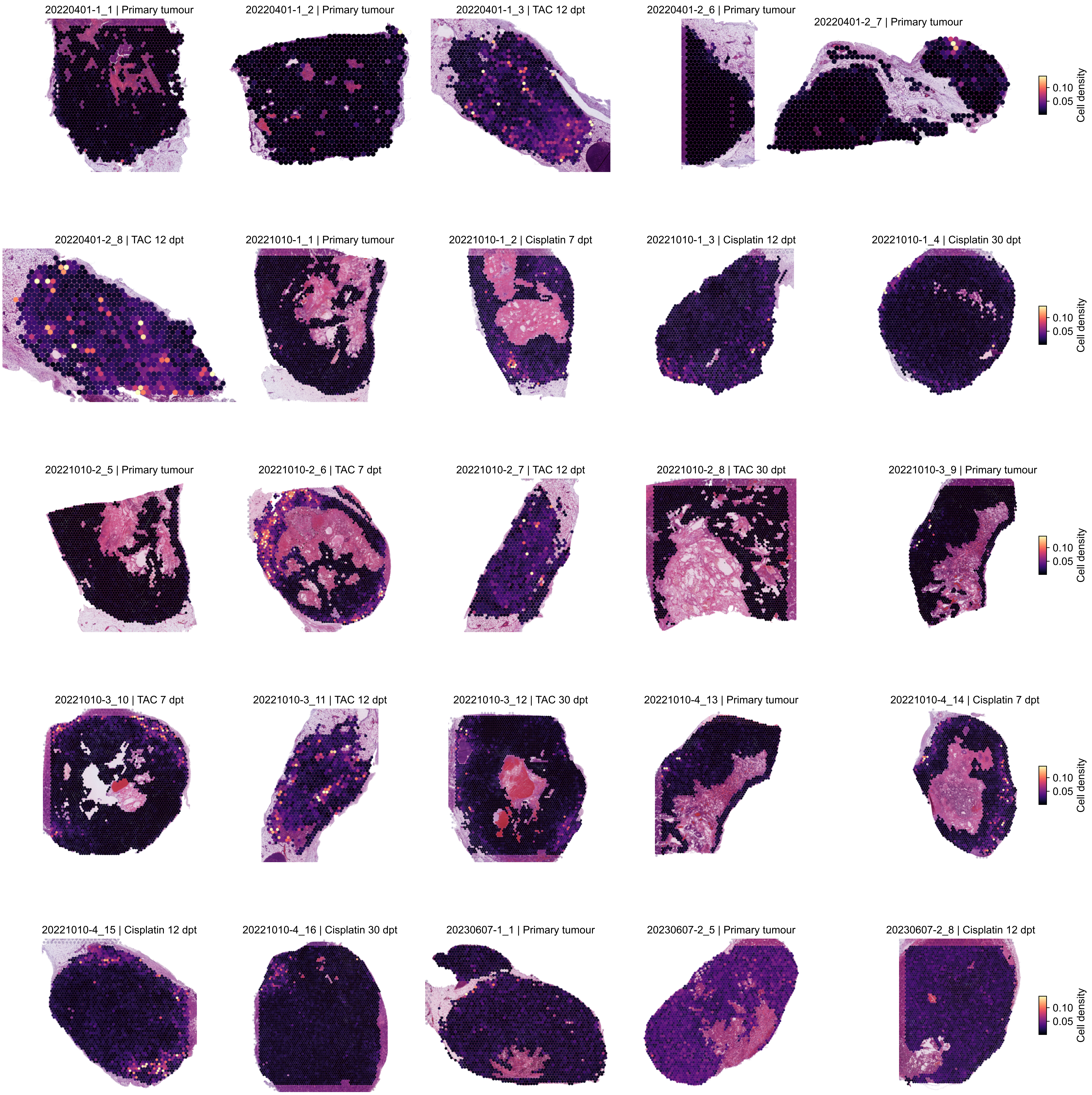

B cell

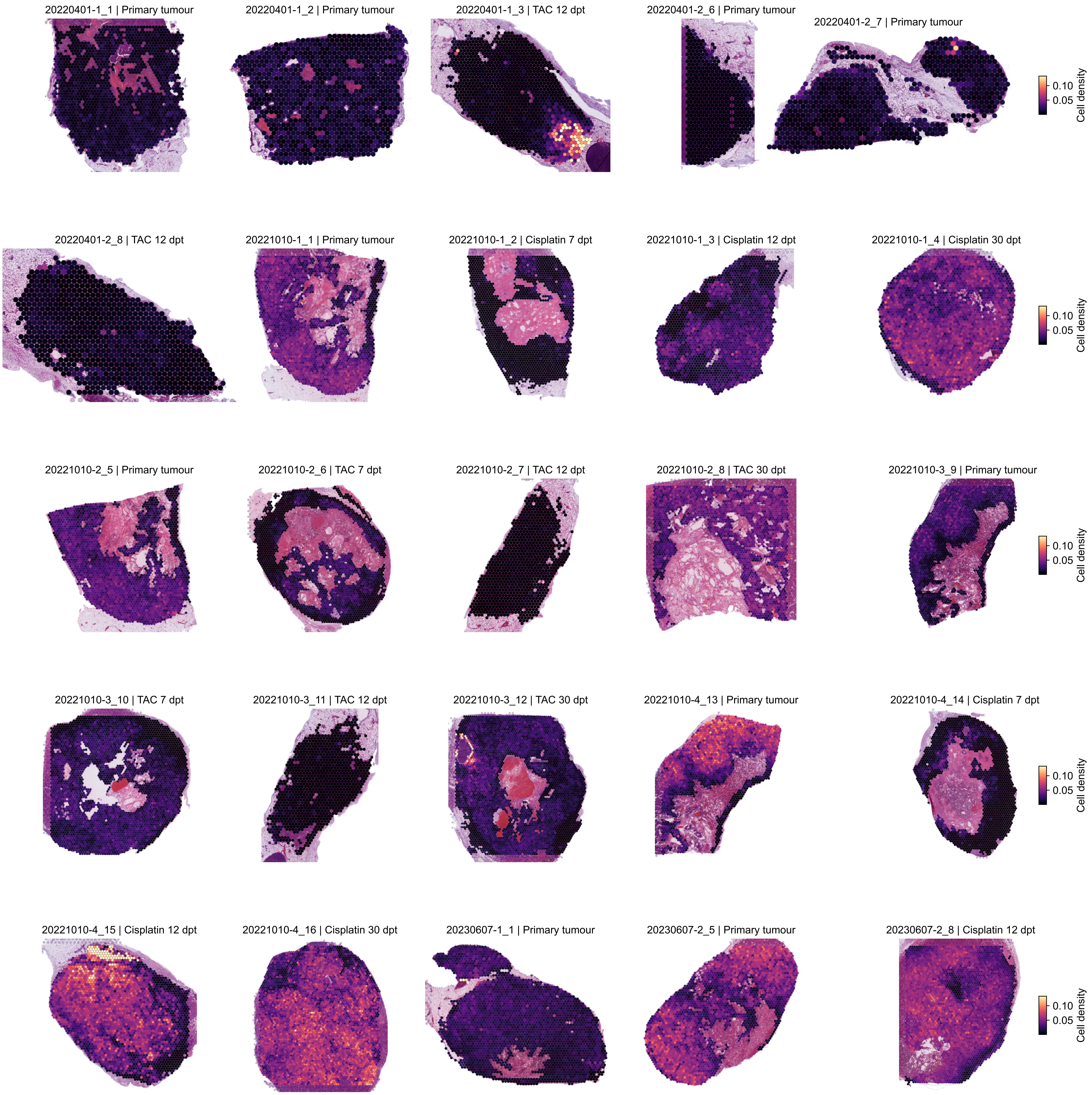

20220401-1_1 | Primary tumour
20220401-1_2 | Primary tumour
20220401-1_3 | TAC 12 dpt
20220401-2_6 | Primary tumour
20220401-2_7 | Primary tumour
0.10
0.05
Cell density
20220401-2_8 | TAC 12 dpt
20221010-1_1 | Primary tumour
20221010-1_2 | Cisplatin 7 dpt
20221010-1_3 | Cisplatin 12 dpt
20221010-1_4 | Cisplatin 30 dpt
0.10
0.05
Cell density
20221010-2_5 | Primary tumour
20221010-2_6 | TAC 7 dpt
20221010-2_7 | TAC 12 dpt
20221010-2_8 | TAC 30 dpt
20221010-3_9 | Primary tumour
0.10
0.05
Cell density
20221010-3_10 | TAC 7 dpt
20221010-3_11 | TAC 12 dpt
20221010-3_12 | TAC 30 dpt
20221010-4_13 | Primary tumour
20221010-4_14 | Cisplatin 7 dpt
0.10
0.05
Cell density
20221010-4_15 | Cisplatin 12 dpt
20221010-4_16 | Cisplatin 30 dpt
20230607-1_1 | Primary tumour
20230607-2_5 | Primary tumour
20230607-2_8 | Cisplatin 12 dpt
0.10
0.05
Cell density

T cell CD4+

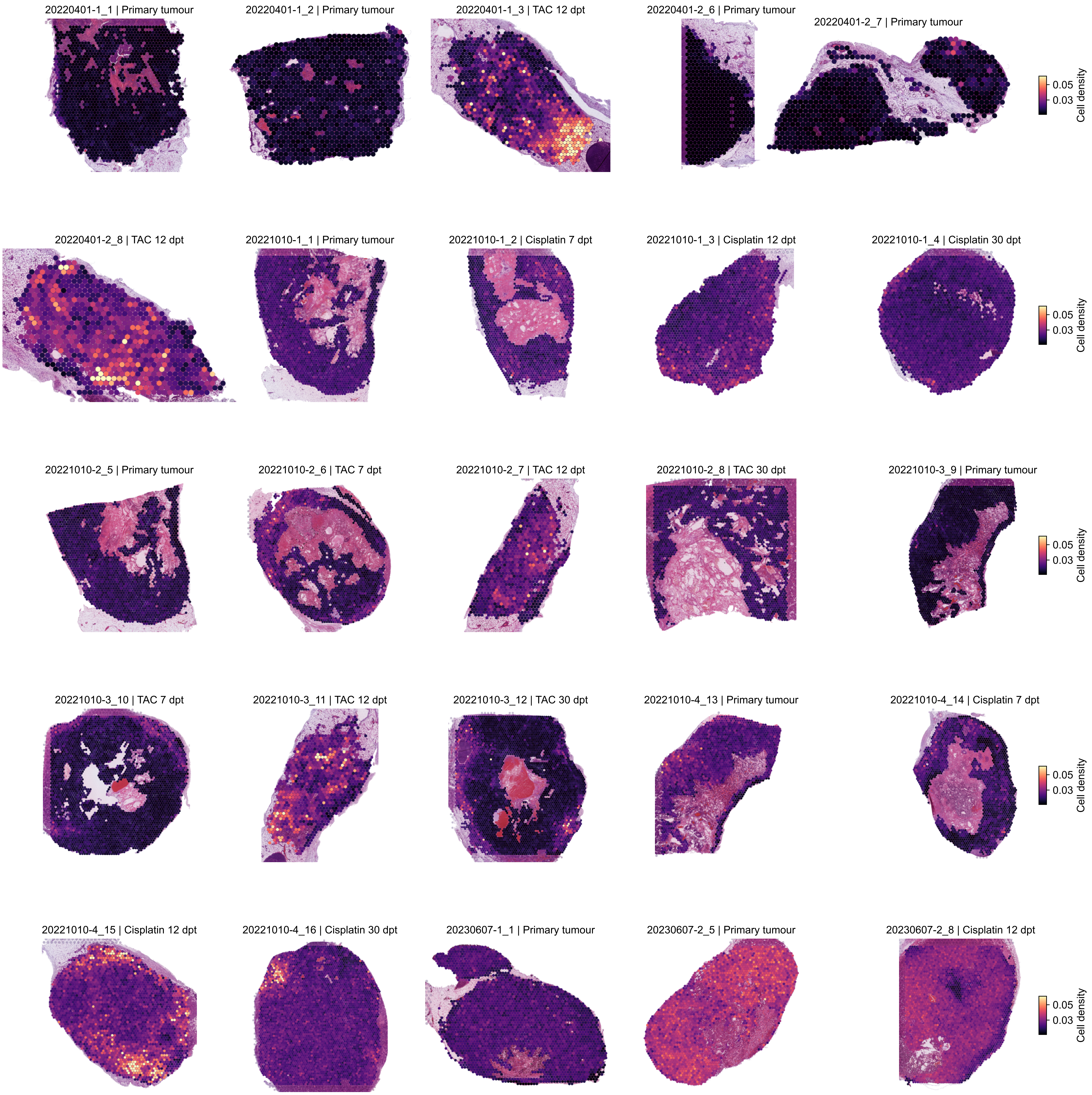
20220401-1_1 | Primary tumour
20220401-1_2 | Primary tumour
20220401-1_3 | TAC 12 dpt
20220401-2_6 | Primary tumour
20220401-2_7 | Primary tumour
0.05
0.03
Cell density
20220401-2_8 | TAC 12 dpt
20221010-1_1 | Primary tumour
20221010-1_2 | Cisplatin 7 dpt
20221010-1_3 | Cisplatin 12 dpt
20221010-1_4 | Cisplatin 30 dpt
0.05
0.03
Cell density
20221010-2_5 | Primary tumour
20221010-2_6 | TAC 7 dpt
20221010-2_7 | TAC 12 dpt
20221010-2_8 | TAC 30 dpt
20221010-3_9 | Primary tumour
0.05
0.03
Cell density
20221010-3_10 | TAC 7 dpt
20221010-3_11 | TAC 12 dpt
20221010-3_12 | TAC 30 dpt
20221010-4_13 | Primary tumour
20221010-4_14 | Cisplatin 7 dpt
0.05
0.03
Cell density
20221010-4_15 | Cisplatin 12 dpt
20221010-4_16 | Cisplatin 30 dpt
20230607-1_1 | Primary tumour
20230607-2_5 | Primary tumour
20230607-2_8 | Cisplatin 12 dpt
0.05
0.03
Cell density

T cell CD8+

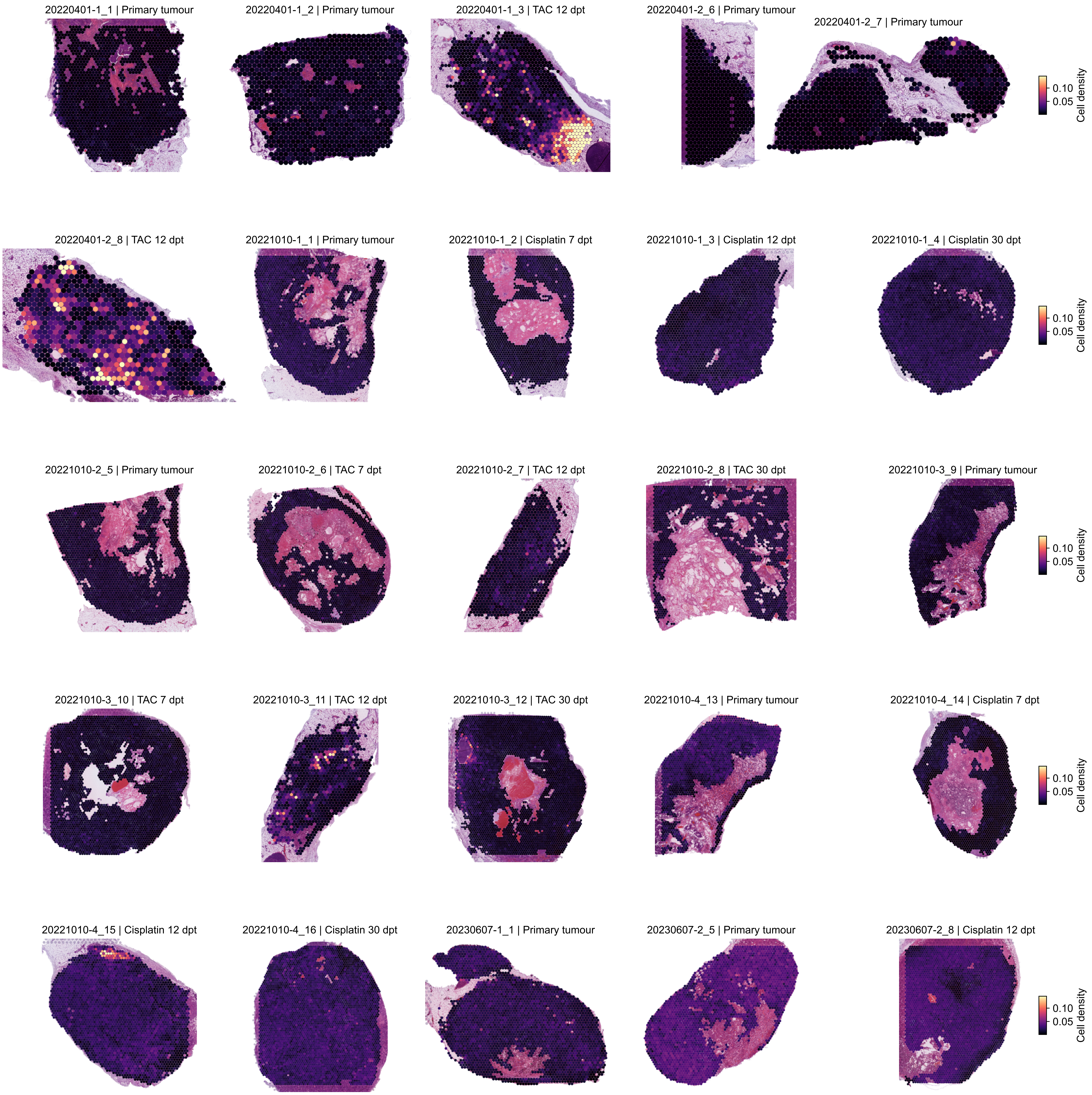
20220401-1_1 | Primary tumour
20220401-1_2 | Primary tumour
20220401-1_3 | TAC 12 dpt
20220401-2_6 | Primary tumour
20220401-2_7 | Primary tumour
0.10
0.05
Cell density
20220401-2_8 | TAC 12 dpt
20221010-1_1 | Primary tumour
20221010-1_2 | Cisplatin 7 dpt
20221010-1_3 | Cisplatin 12 dpt
20221010-1_4 | Cisplatin 30 dpt
0.10
0.05
Cell density
20221010-2_5 | Primary tumour
20221010-2_6 | TAC 7 dpt
20221010-2_7 | TAC 12 dpt
20221010-2_8 | TAC 30 dpt
20221010-3_9 | Primary tumour
0.10
0.05
Cell density
20221010-3_10 | TAC 7 dpt
20221010-3_11 | TAC 12 dpt
20221010-3_12 | TAC 30 dpt
20221010-4_13 | Primary tumour
20221010-4_14 | Cisplatin 7 dpt
0.10
0.05
Cell density
20221010-4_15 | Cisplatin 12 dpt
20221010-4_16 | Cisplatin 30 dpt
20230607-1_1 | Primary tumour
20230607-2_5 | Primary tumour
20230607-2_8 | Cisplatin 12 dpt
0.10
0.05
Cell density

Dendritic cell

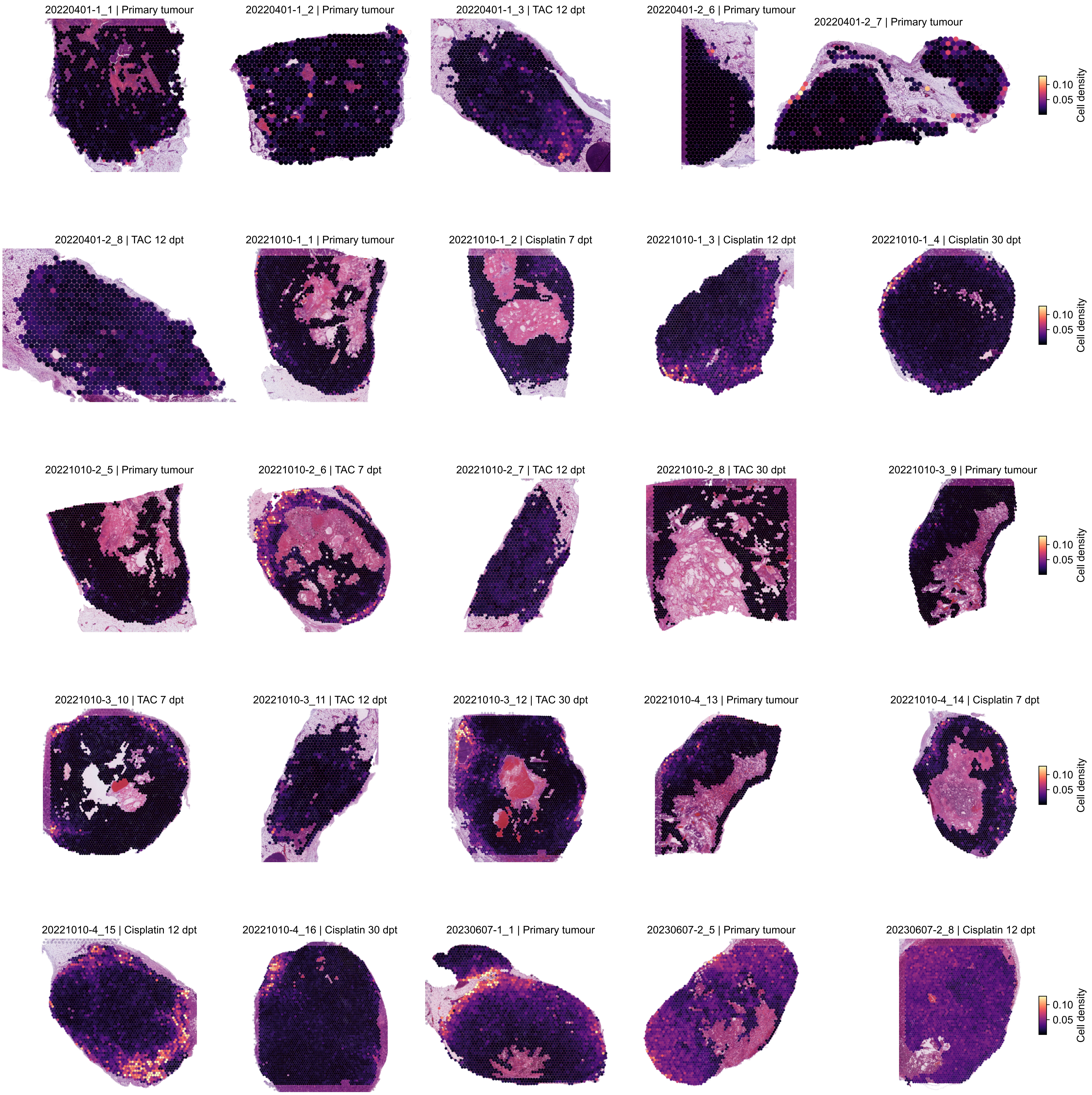

Macrophage CH

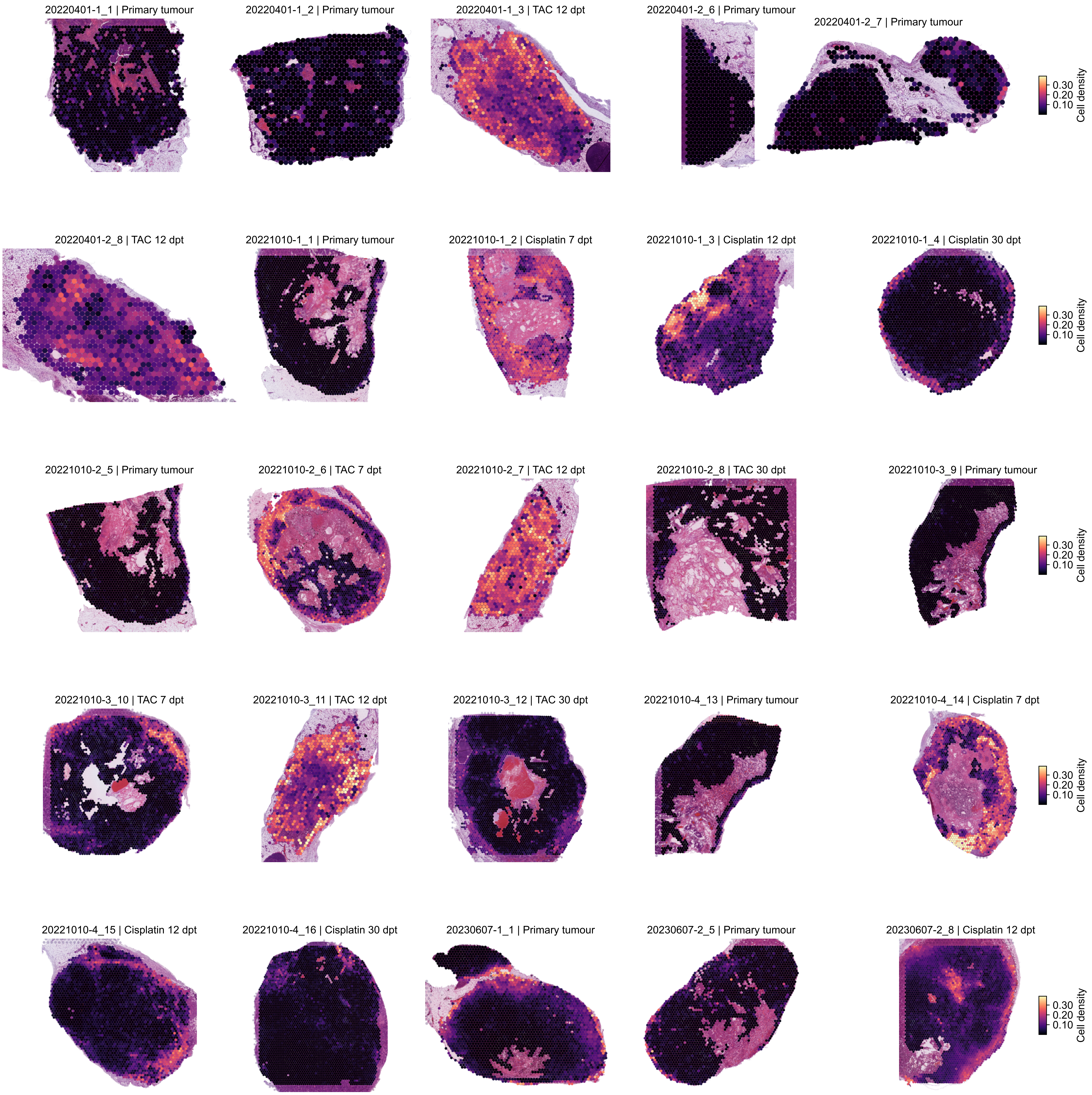

Endothelial cell

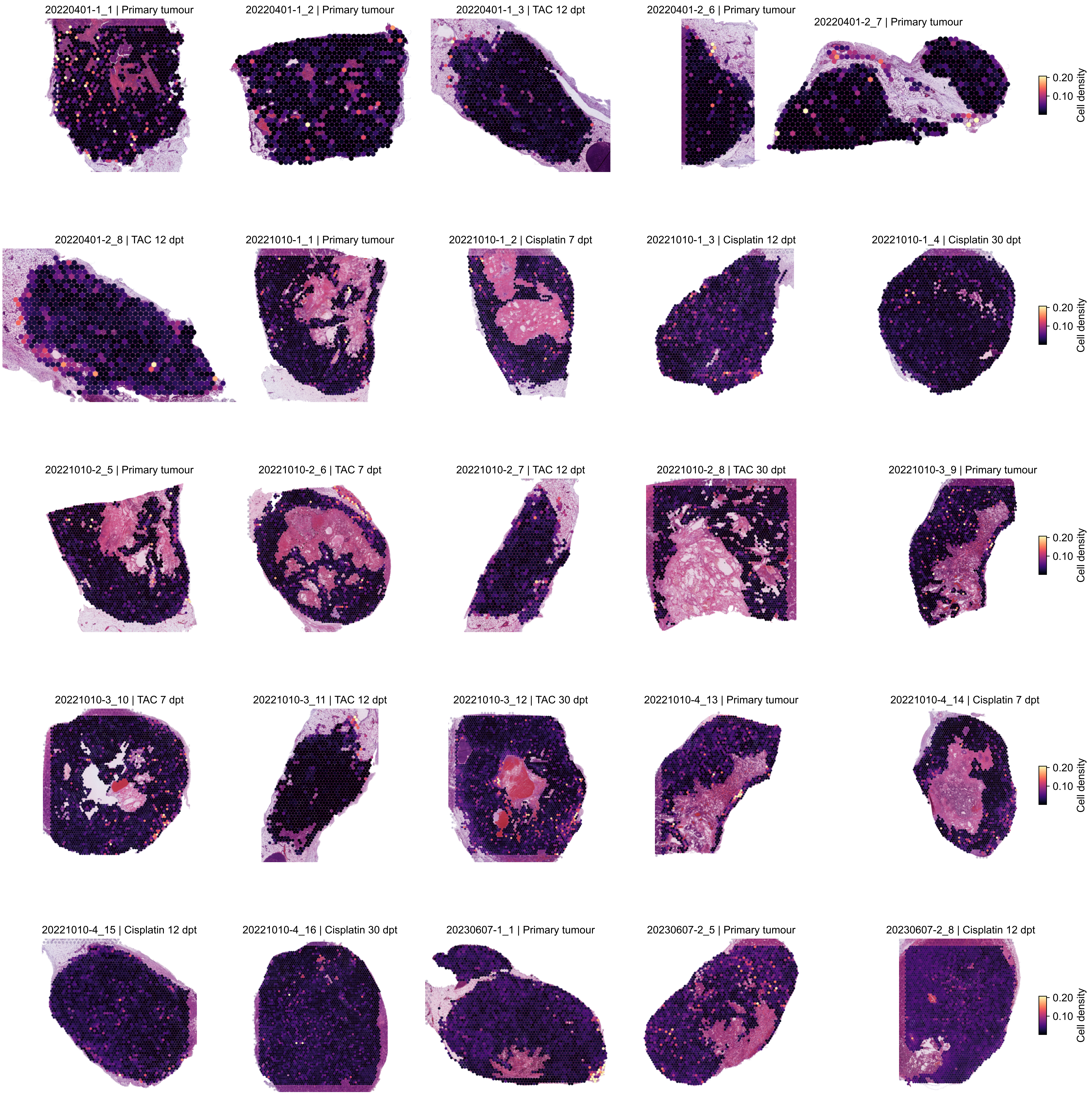

Fibroblast

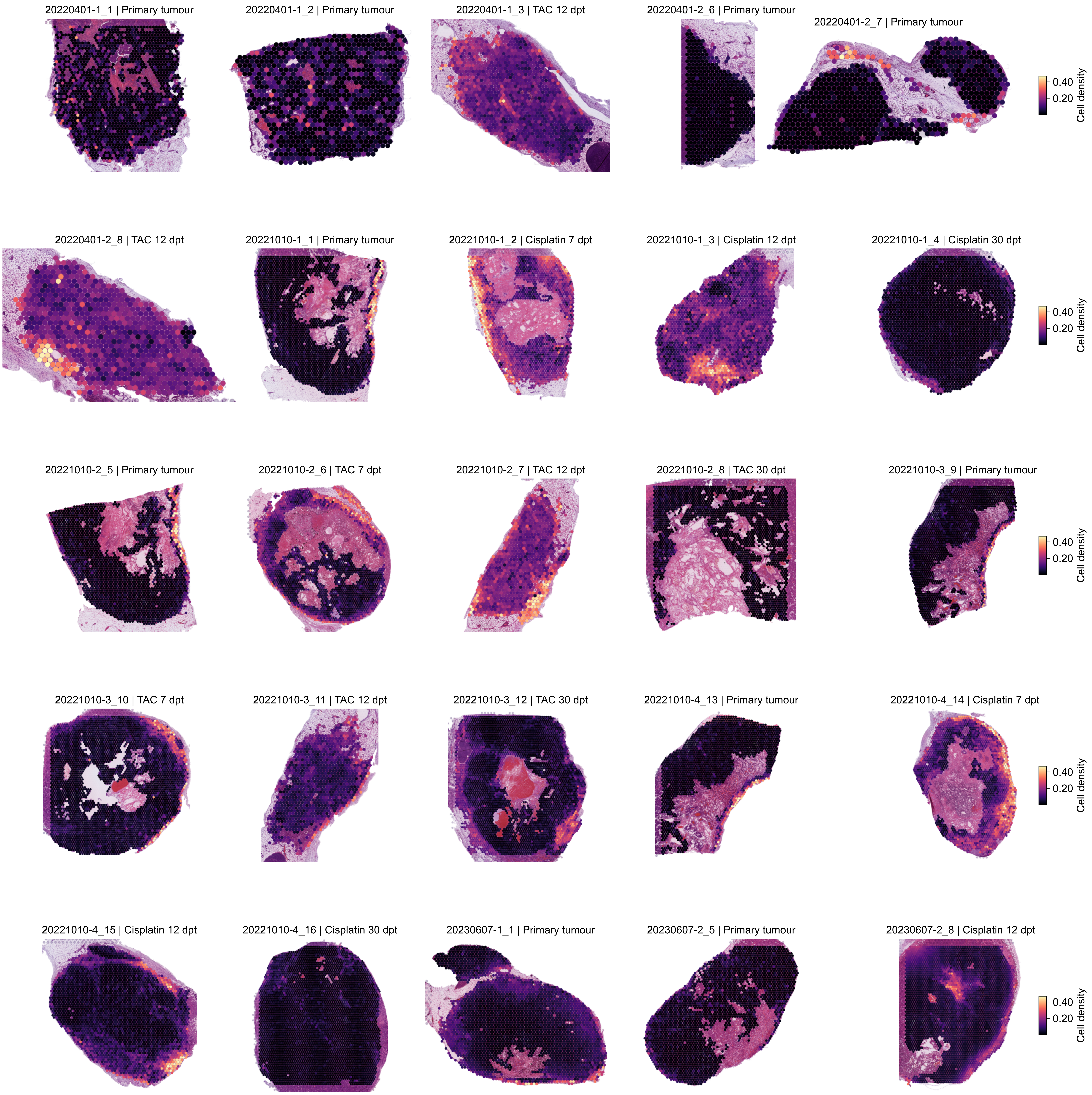
20220401-1_1 | Primary tumour
20220401-1_2 | Primary tumour
20220401-1_3 | TAC 12 dpt
20220401-2_6 | Primary tumour
20220401-2_7 | Primary tumour
20220401-2_8 | TAC 12 dpt
20221010-1_1 | Primary tumour
20221010-1_2 | Cisplatin 7 dpt
20221010-1_3 | Cisplatin 12 dpt
20221010-1_4 | Cisplatin 30 dpt
20221010-2_5 | Primary tumour
20221010-2_6 | TAC 7 dpt
20221010-2_7 | TAC 12 dpt
20221010-2_8 | TAC 30 dpt
20221010-3_9 | Primary tumour
20221010-3_10 | TAC 7 dpt
20221010-3_11 | TAC 12 dpt
20221010-3_12 | TAC 30 dpt
20221010-4_13 | Primary tumour
20221010-4_14 | Cisplatin 7 dpt
20221010-4_15 | Cisplatin 12 dpt
20221010-4_16 | Cisplatin 30 dpt
20230607-1_1 | Primary tumour
20230607-2_5 | Primary tumour
20230607-2_8 | Cisplatin 12 dpt
0.40
0.20
Cell density

Macrophage Inf.

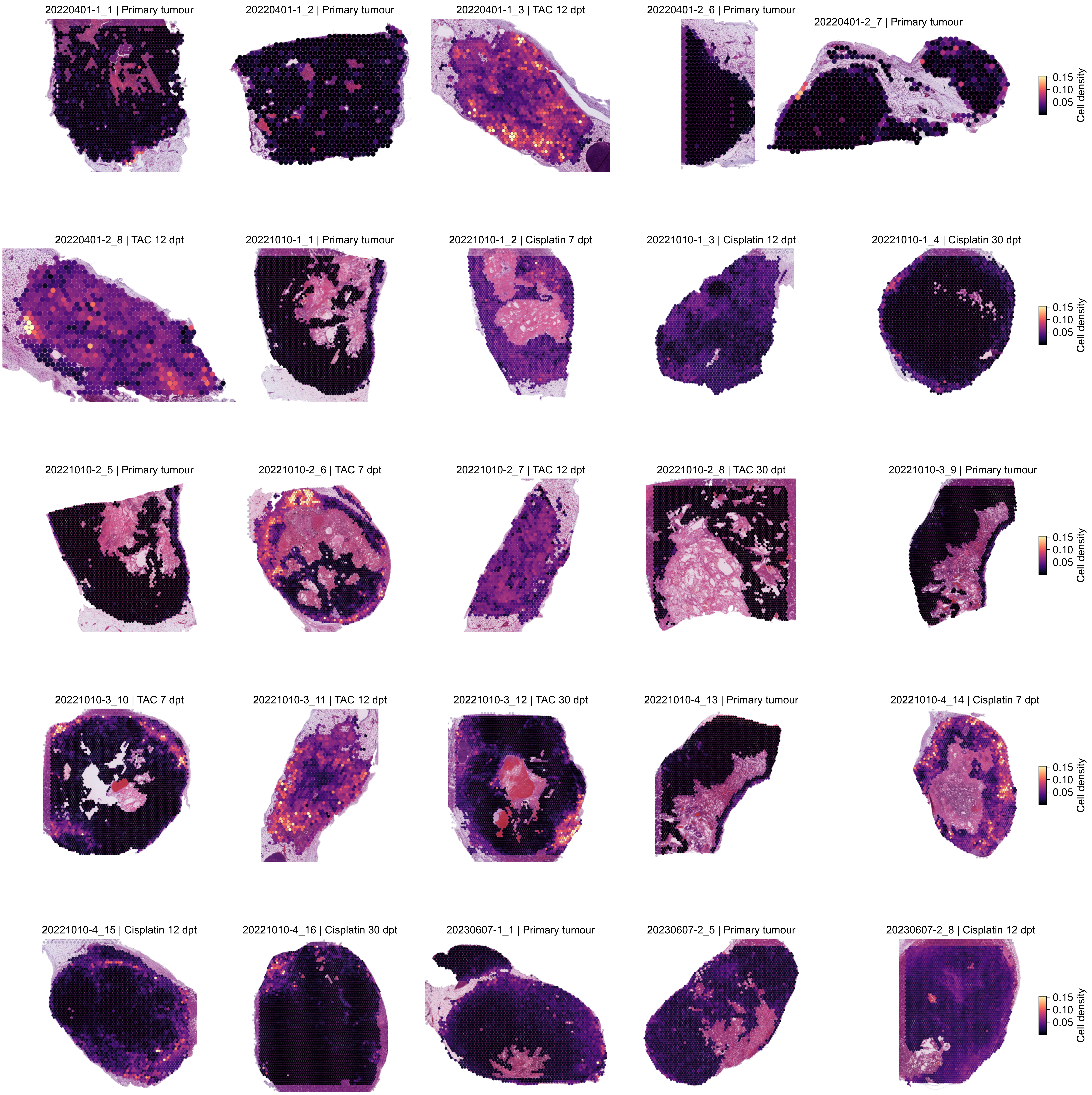

NK cell

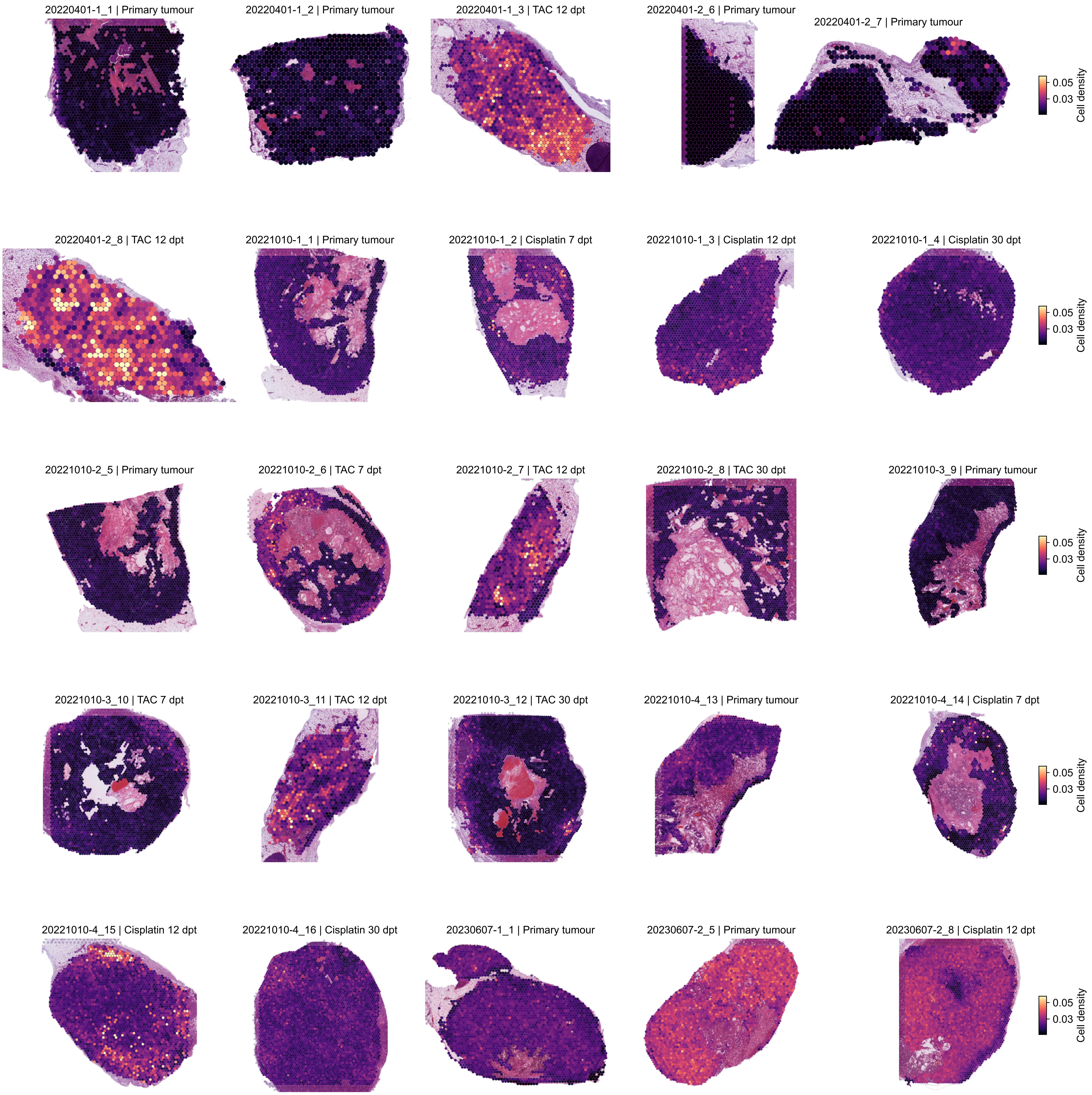

20220401-1_1 | Primary tumour
20220401-1_2 | Primary tumour
20220401-1_3 | TAC 12 dpt
20220401-2_6 | Primary tumour
20220401-2_7 | Primary tumour
20220401-2_8 | TAC 12 dpt
20221010-1_1 | Primary tumour
20221010-1_2 | Cisplatin 7 dpt
20221010-1_3 | Cisplatin 12 dpt
20221010-1_4 | Cisplatin 30 dpt
20221010-2_5 | Primary tumour
20221010-2_6 | TAC 7 dpt
20221010-2_7 | TAC 12 dpt
20221010-2_8 | TAC 30 dpt
20221010-3_9 | Primary tumour
20221010-3_10 | TAC 7 dpt
20221010-3_11 | TAC 12 dpt
20221010-3_12 | TAC 30 dpt
20221010-4_13 | Primary tumour
20221010-4_14 | Cisplatin 7 dpt
20221010-4_15 | Cisplatin 12 dpt
20221010-4_16 | Cisplatin 30 dpt
20230607-1_1 | Primary tumour
20230607-2_5 | Primary tumour
20230607-2_8 | Cisplatin 12 dpt
0.05
0.03
Cell density

Dendritic cell plasmacytoid

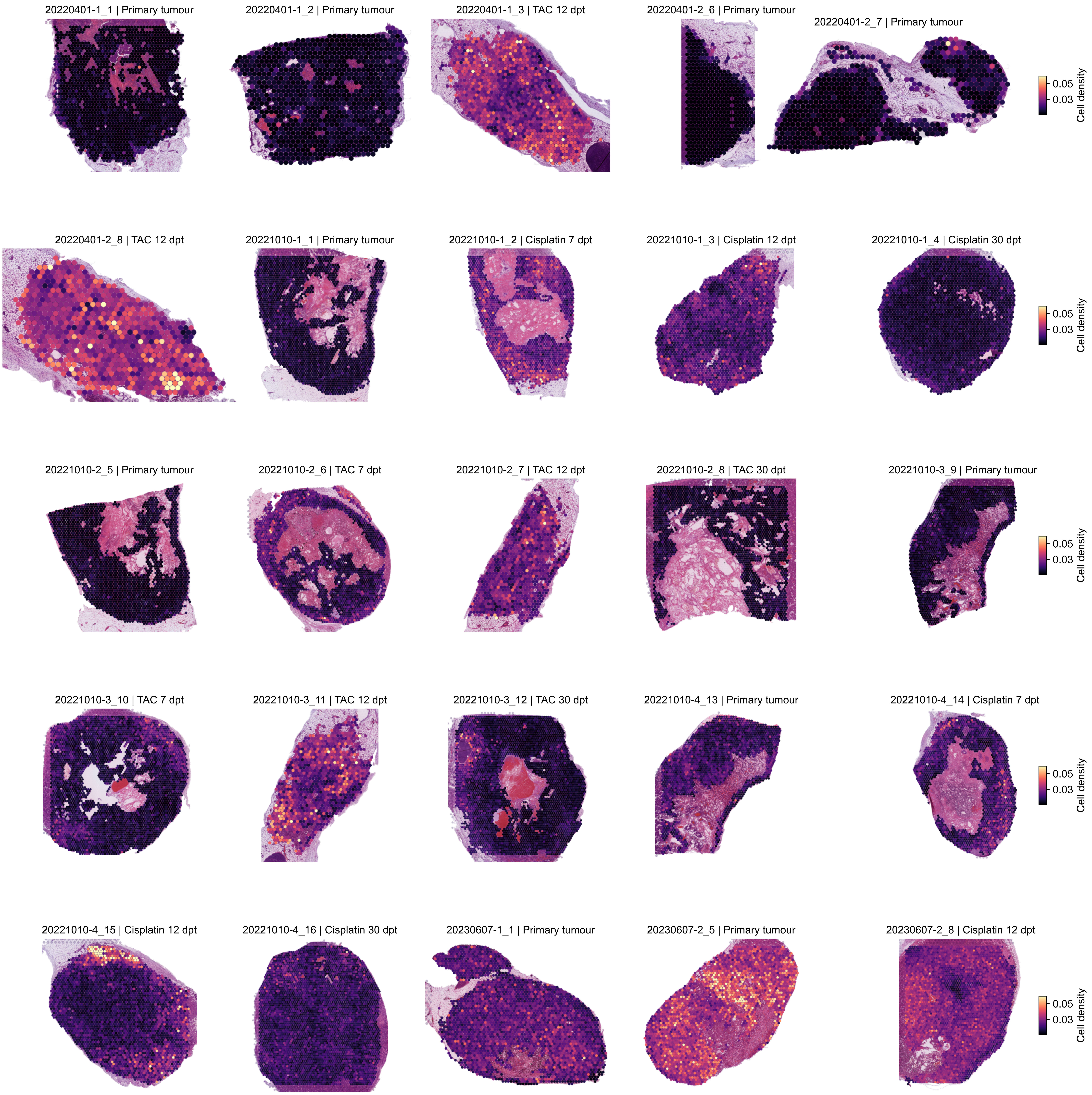

Plasma cell

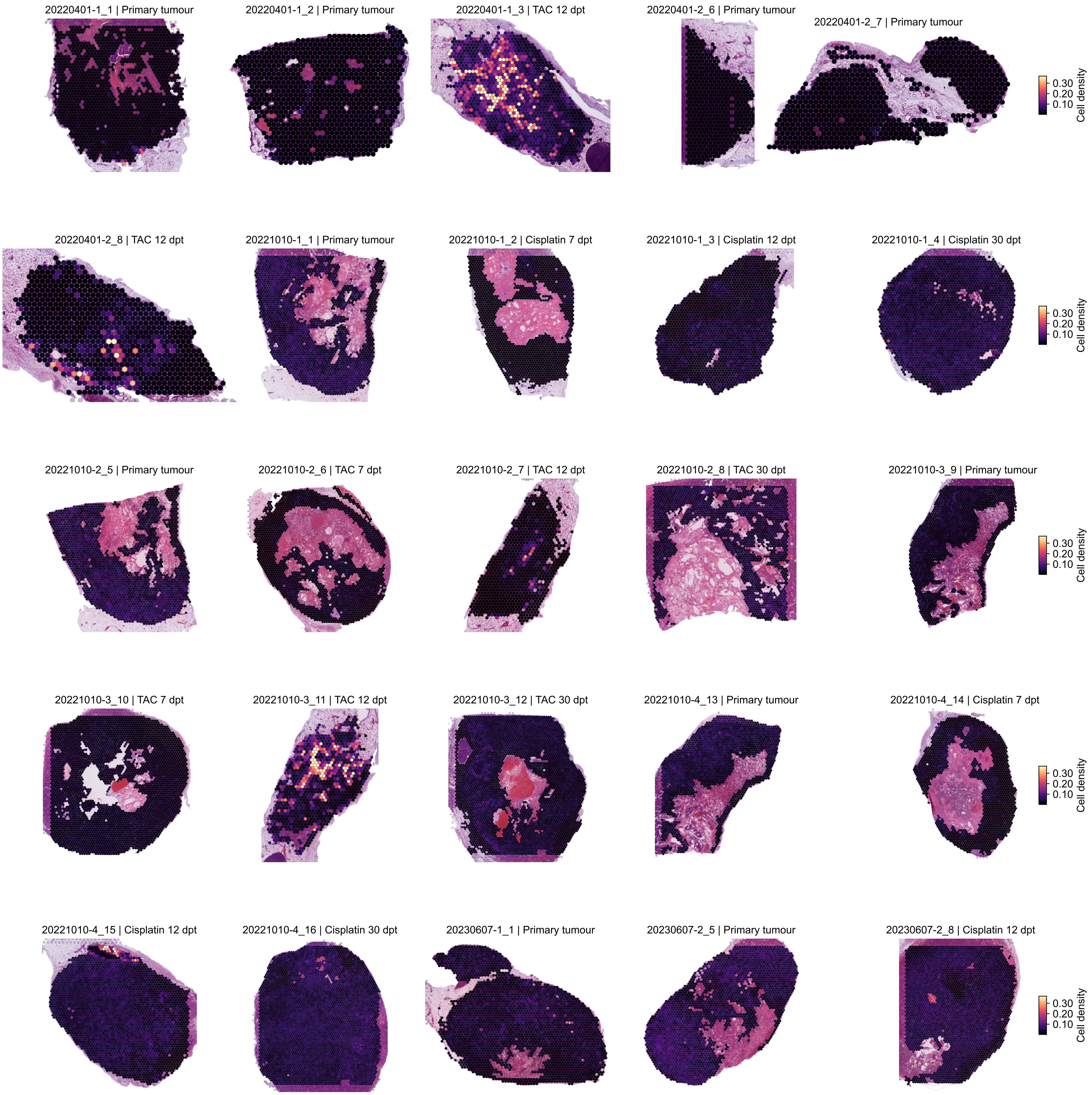
20220401-1_1 | Primary tumour
20220401-1_2 | Primary tumour
20220401-1_3 | TAC 12 dpt
20220401-2_6 | Primary tumour
20220401-2_7 | Primary tumour
20220401-2_8 | TAC 12 dpt
20221010-1_1 | Primary tumour
20221010-1_2 | Cisplatin 7 dpt
20221010-1_3 | Cisplatin 12 dpt
20221010-1_4 | Cisplatin 30 dpt
20221010-2_5 | Primary tumour
20221010-2_6 | TAC 7 dpt
20221010-2_7 | TAC 12 dpt
20221010-2_8 | TAC 30 dpt
20221010-3_9 | Primary tumour
20221010-3_10 | TAC 7 dpt
20221010-3_11 | TAC 12 dpt
20221010-3_12 | TAC 30 dpt
20221010-4_13 | Primary tumour
20221010-4_14 | Cisplatin 7 dpt
20221010-4_15 | Cisplatin 12 dpt
20221010-4_16 | Cisplatin 30 dpt
20230607-1_1 | Primary tumour
20230607-2_5 | Primary tumour
20230607-2_8 | Cisplatin 12 dpt
0.30
0.20
0.10
Cell density

Macrophage SPP1+

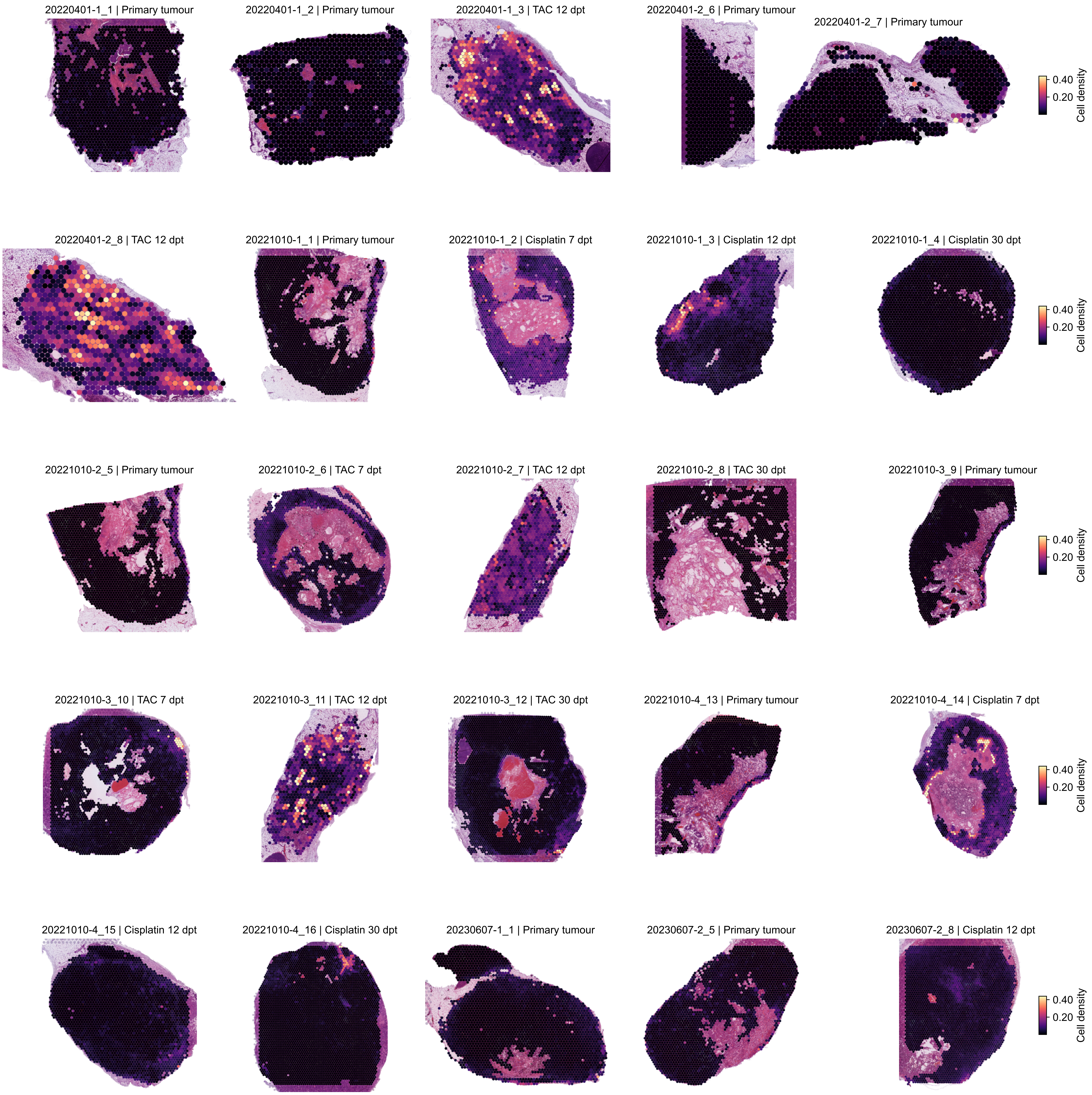

Tumour basal-EMT

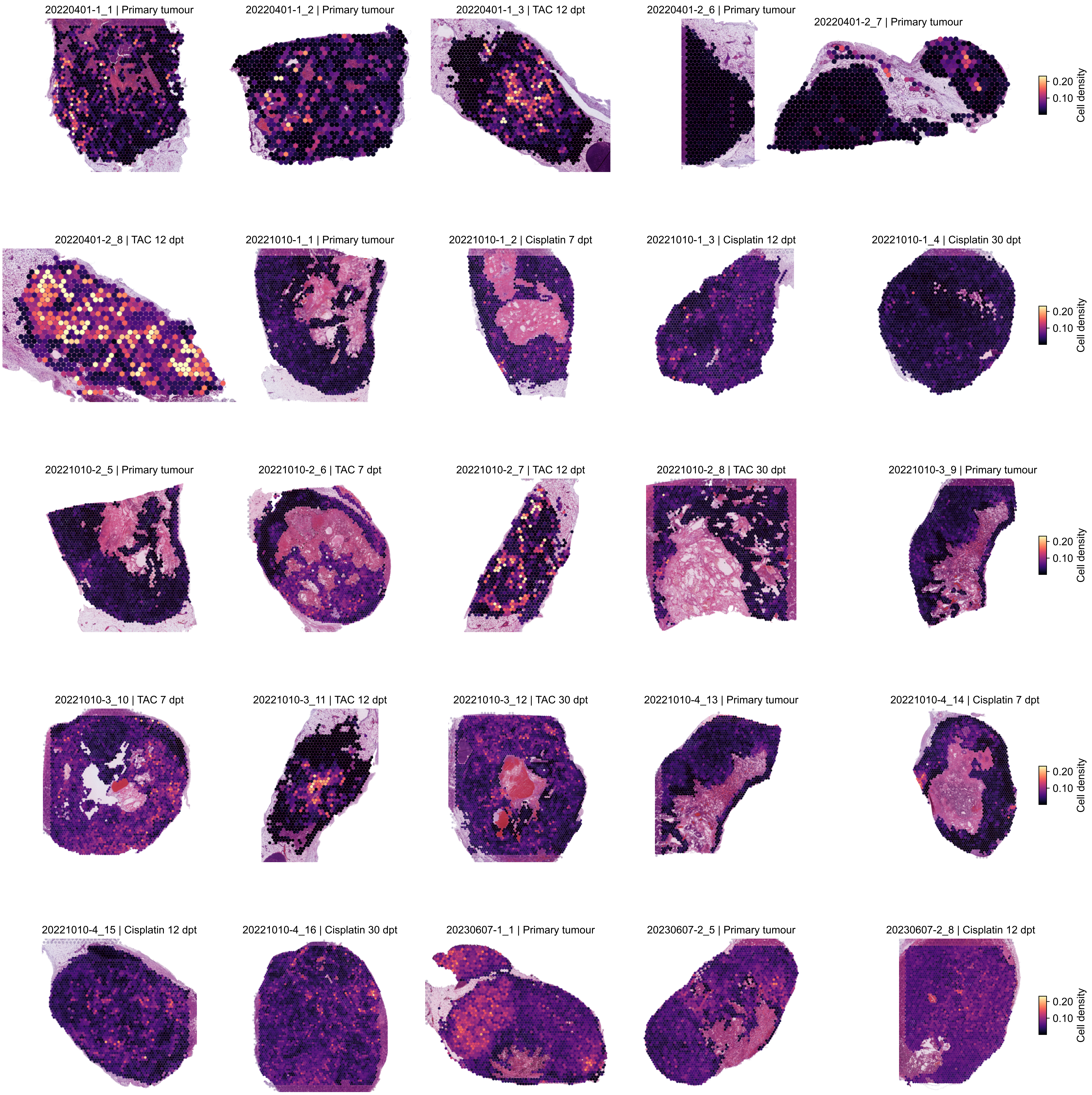

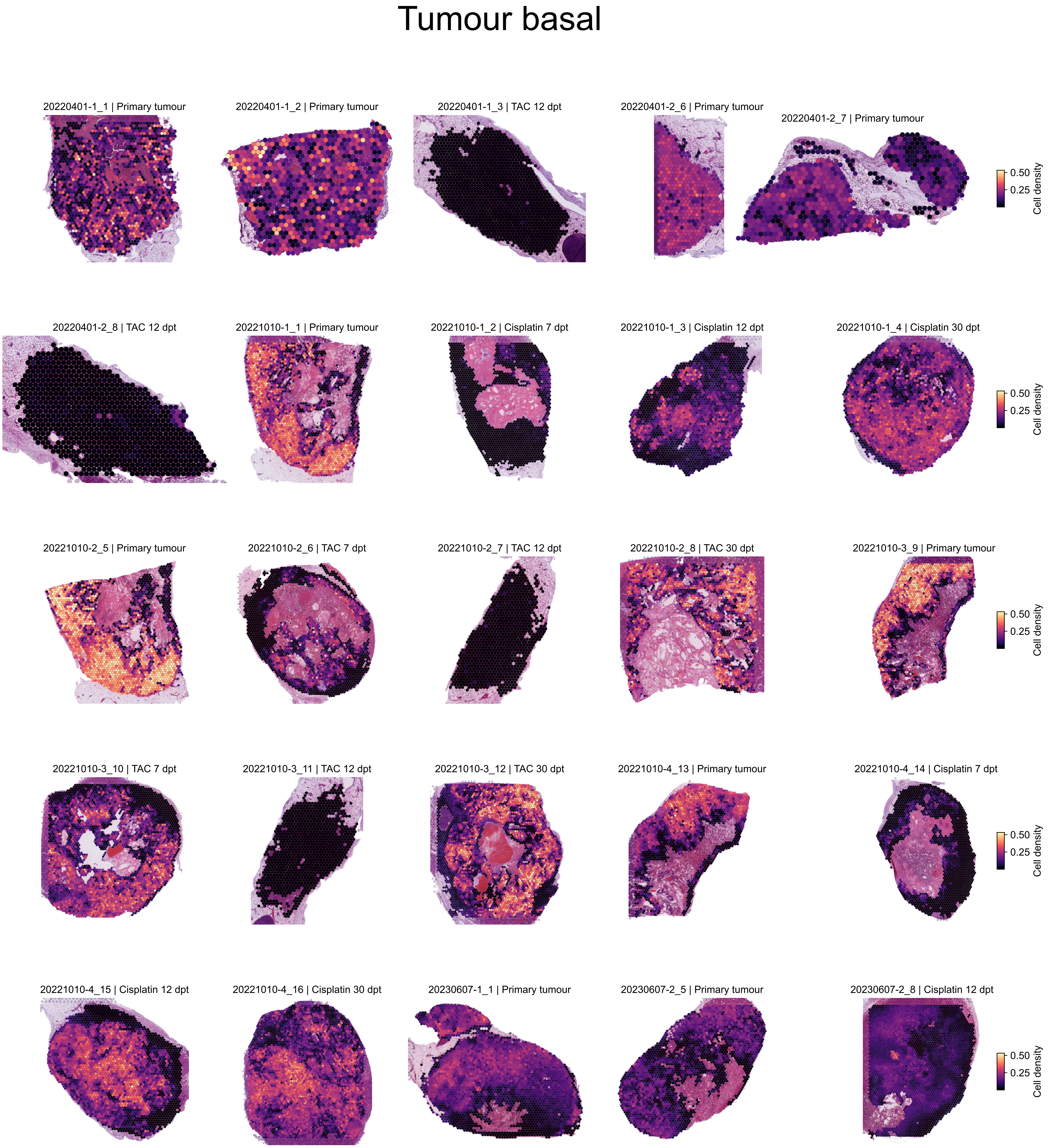
Tumour basal
20220401-1_1 | Primary tumour
20220401-1_2 | Primary tumour
20220401-1_3 | TAC 12 dpt
20220401-2_6 | Primary tumour
20220401-2_7 | Primary tumour
20220401-2_8 | TAC 12 dpt
20221010-1_1 | Primary tumour
20221010-1_2 | Cisplatin 7 dpt
20221010-1_3 | Cisplatin 12 dpt
20221010-1_4 | Cisplatin 30 dpt
20221010-2_5 | Primary tumour
20221010-2_6 | TAC 7 dpt
20221010-2_7 | TAC 12 dpt
20221010-2_8 | TAC 30 dpt
20221010-3_9 | Primary tumour
20221010-3_10 | TAC 7 dpt
20221010-3_11 | TAC 12 dpt
20221010-3_12 | TAC 30 dpt
20221010-4_13 | Primary tumour
20221010-4_14 | Cisplatin 7 dpt
20221010-4_15 | Cisplatin 12 dpt
20221010-4_16 | Cisplatin 30 dpt
20230607-1_1 | Primary tumour
20230607-2_5 | Primary tumour
20230607-2_8 | Cisplatin 12 dpt
0.50
0.25
Cell density

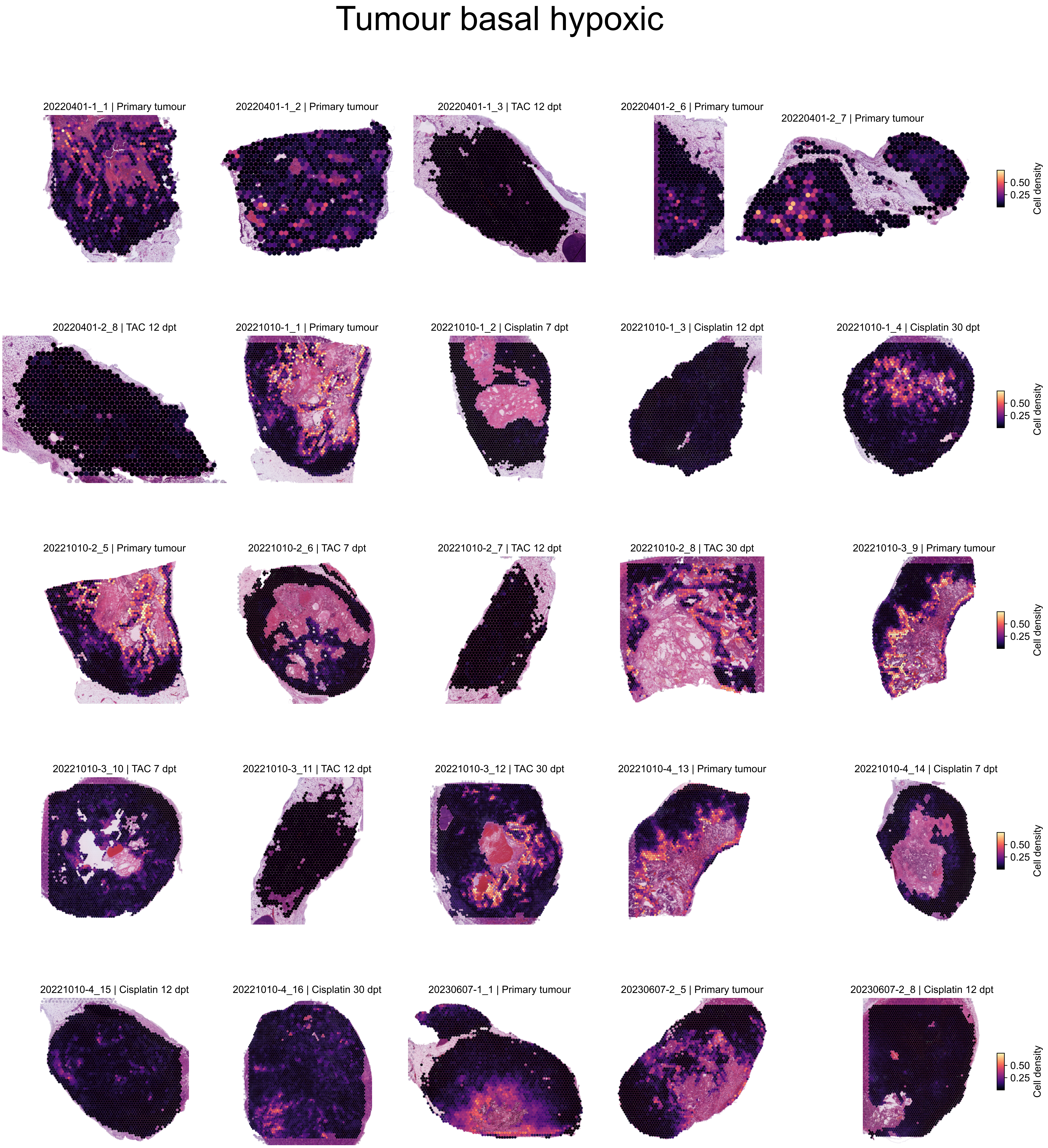
Tumour basal hypoxic
20220401-1_1 | Primary tumour
20220401-1_2 | Primary tumour
20220401-1_3 | TAC 12 dpt
20220401-2_6 | Primary tumour
20220401-2_7 | Primary tumour
20220401-2_8 | TAC 12 dpt
20221010-1_1 | Primary tumour
20221010-1_2 | Cisplatin 7 dpt
20221010-1_3 | Cisplatin 12 dpt
20221010-1_4 | Cisplatin 30 dpt
20221010-2_5 | Primary tumour
20221010-2_6 | TAC 7 dpt
20221010-2_7 | TAC 12 dpt
20221010-2_8 | TAC 30 dpt
20221010-3_9 | Primary tumour
20221010-3_10 | TAC 7 dpt
20221010-3_11 | TAC 12 dpt
20221010-3_12 | TAC 30 dpt
20221010-4_13 | Primary tumour
20221010-4_14 | Cisplatin 7 dpt
20221010-4_15 | Cisplatin 12 dpt
20221010-4_16 | Cisplatin 30 dpt
20230607-1_1 | Primary tumour
20230607-2_5 | Primary tumour
20230607-2_8 | Cisplatin 12 dpt
0.50
0.25
Cell density

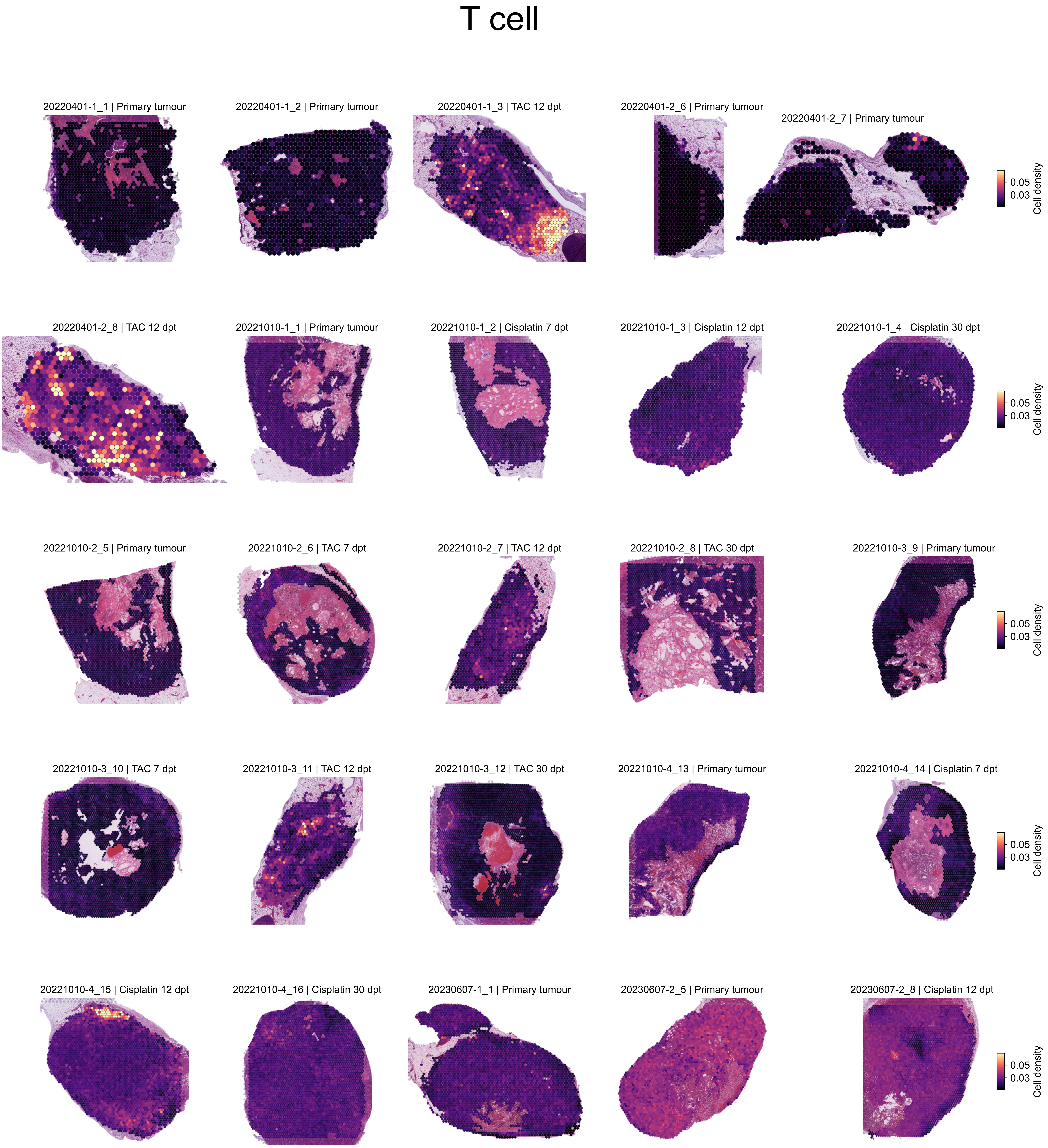
T cell
20220401-1_1 | Primary tumour
20220401-1_2 | Primary tumour
20220401-1_3 | TAC 12 dpt
20220401-2_6 | Primary tumour
20220401-2_7 | Primary tumour
20220401-2_8 | TAC 12 dpt
20221010-1_1 | Primary tumour
20221010-1_2 | Cisplatin 7 dpt
20221010-1_3 | Cisplatin 12 dpt
20221010-1_4 | Cisplatin 30 dpt
20221010-2_5 | Primary tumour
20221010-2_6 | TAC 7 dpt
20221010-2_7 | TAC 12 dpt
20221010-2_8 | TAC 30 dpt
20221010-3_9 | Primary tumour
20221010-3_10 | TAC 7 dpt
20221010-3_11 | TAC 12 dpt
20221010-3_12 | TAC 30 dpt
20221010-4_13 | Primary tumour
20221010-4_14 | Cisplatin 7 dpt
20221010-4_15 | Cisplatin 12 dpt
20221010-4_16 | Cisplatin 30 dpt
20230607-1_1 | Primary tumour
20230607-2_5 | Primary tumour
20230607-2_8 | Cisplatin 12 dpt
0.05
0.03
Cell density

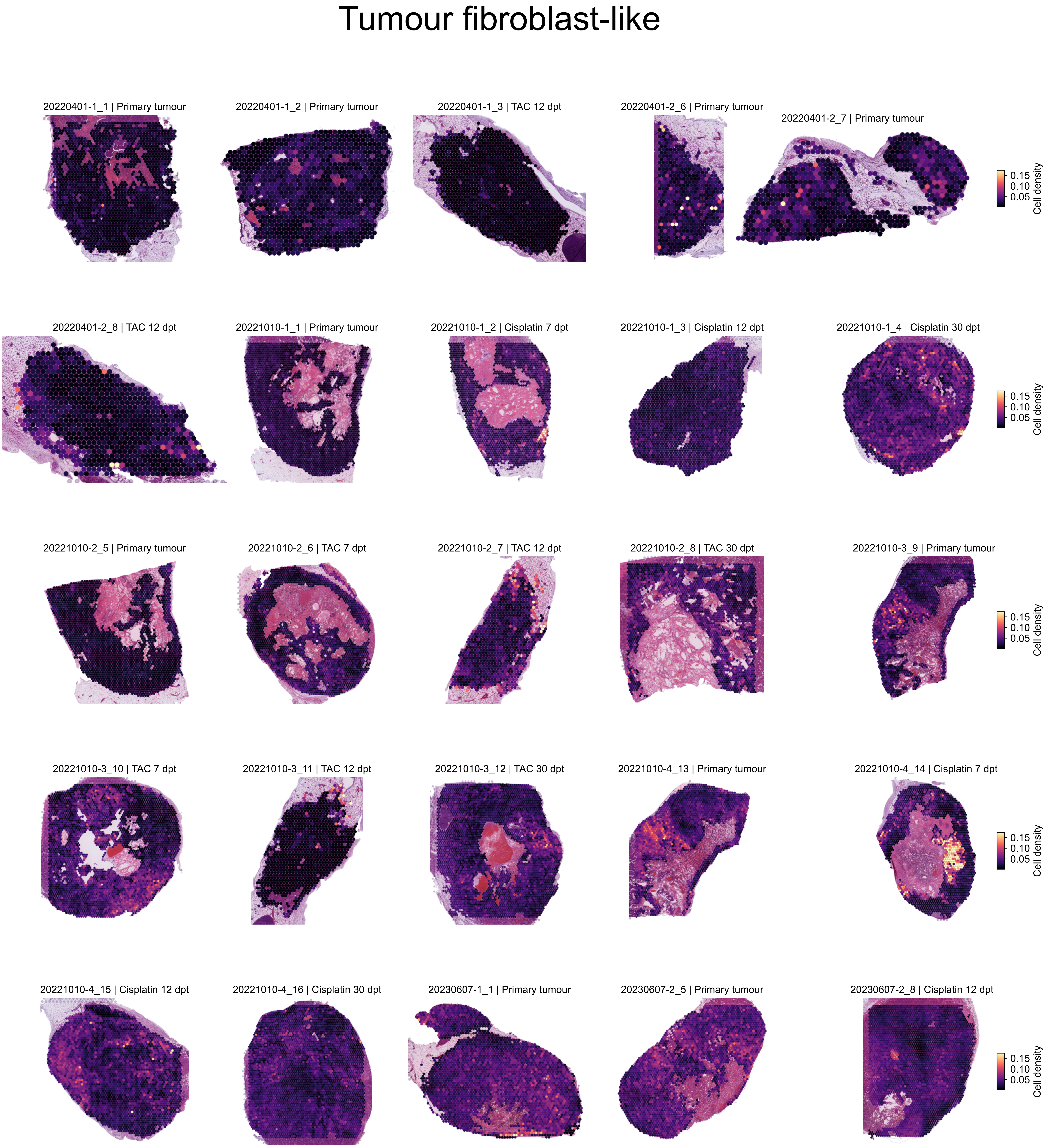
Tumour fibroblast-like
20220401-1_1 | Primary tumour
20220401-1_2 | Primary tumour
20220401-1_3 | TAC 12 dpt
20220401-2_6 | Primary tumour
20220401-2_7 | Primary tumour
20220401-2_8 | TAC 12 dpt
20221010-1_1 | Primary tumour
20221010-1_2 | Cisplatin 7 dpt
20221010-1_3 | Cisplatin 12 dpt
20221010-1_4 | Cisplatin 30 dpt
20221010-2_5 | Primary tumour
20221010-2_6 | TAC 7 dpt
20221010-2_7 | TAC 12 dpt
20221010-2_8 | TAC 30 dpt
20221010-3_9 | Primary tumour
20221010-3_10 | TAC 7 dpt
20221010-3_11 | TAC 12 dpt
20221010-3_12 | TAC 30 dpt
20221010-4_13 | Primary tumour
20221010-4_14 | Cisplatin 7 dpt
20221010-4_15 | Cisplatin 12 dpt
20221010-4_16 | Cisplatin 30 dpt
20230607-1_1 | Primary tumour
20230607-2_5 | Primary tumour
20230607-2_8 | Cisplatin 12 dpt
0.15
0.10
0.05
Cell density

Tumour luminal-alveolar

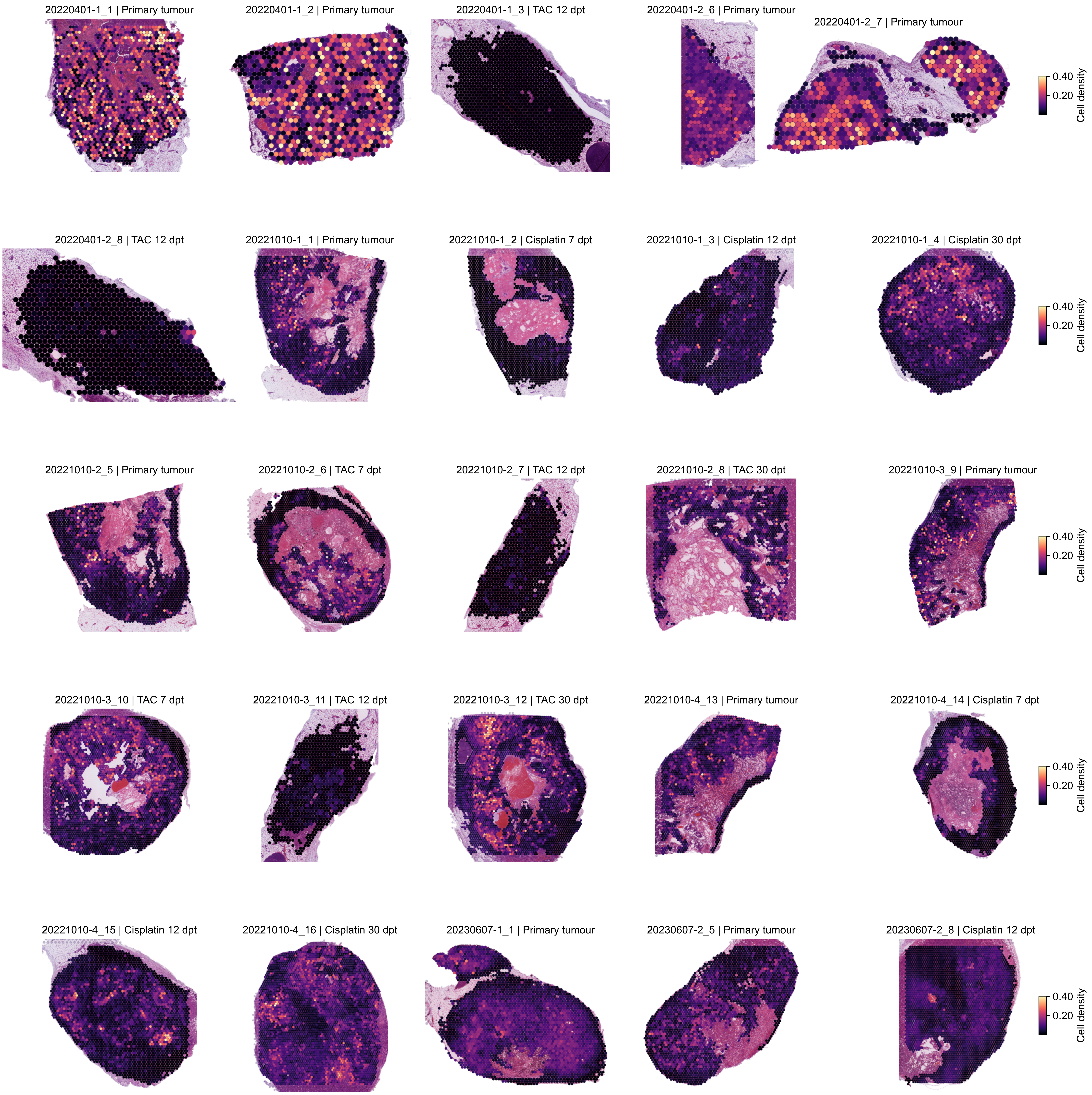

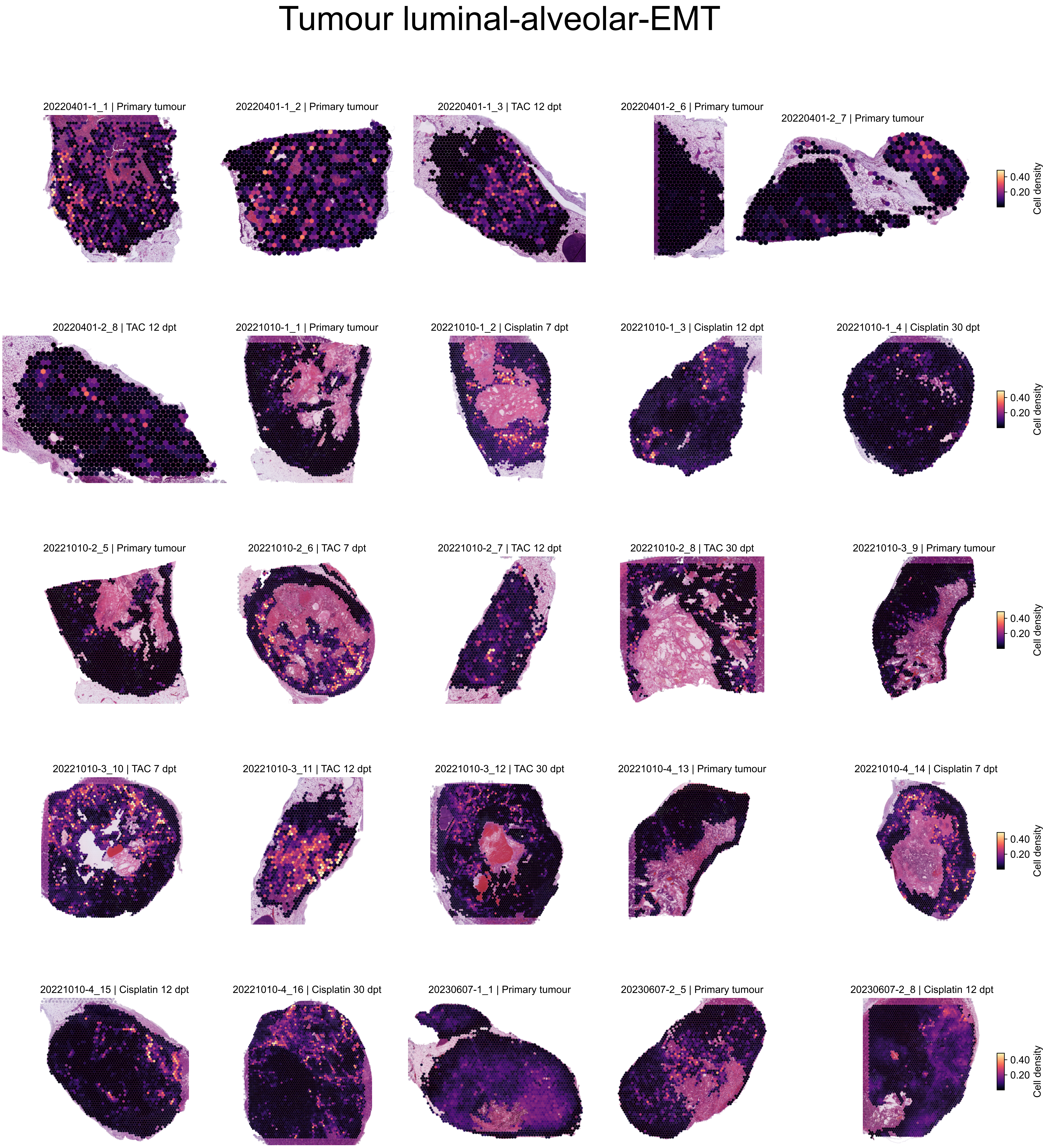
Tumour luminal-alveolar-EMT
20220401-1_1 | Primary tumour
20220401-1_2 | Primary tumour
20220401-1_3 | TAC 12 dpt
20220401-2_6 | Primary tumour
20220401-2_7 | Primary tumour
20220401-2_8 | TAC 12 dpt
20221010-1_1 | Primary tumour
20221010-1_2 | Cisplatin 7 dpt
20221010-1_3 | Cisplatin 12 dpt
20221010-1_4 | Cisplatin 30 dpt
20221010-2_5 | Primary tumour
20221010-2_6 | TAC 7 dpt
20221010-2_7 | TAC 12 dpt
20221010-2_8 | TAC 30 dpt
20221010-3_9 | Primary tumour
20221010-3_10 | TAC 7 dpt
20221010-3_11 | TAC 12 dpt
20221010-3_12 | TAC 30 dpt
20221010-4_13 | Primary tumour
20221010-4_14 | Cisplatin 7 dpt
20221010-4_15 | Cisplatin 12 dpt
20221010-4_16 | Cisplatin 30 dpt
20230607-1_1 | Primary tumour
20230607-2_5 | Primary tumour
20230607-2_8 | Cisplatin 12 dpt
0.40
0.20
Cell density

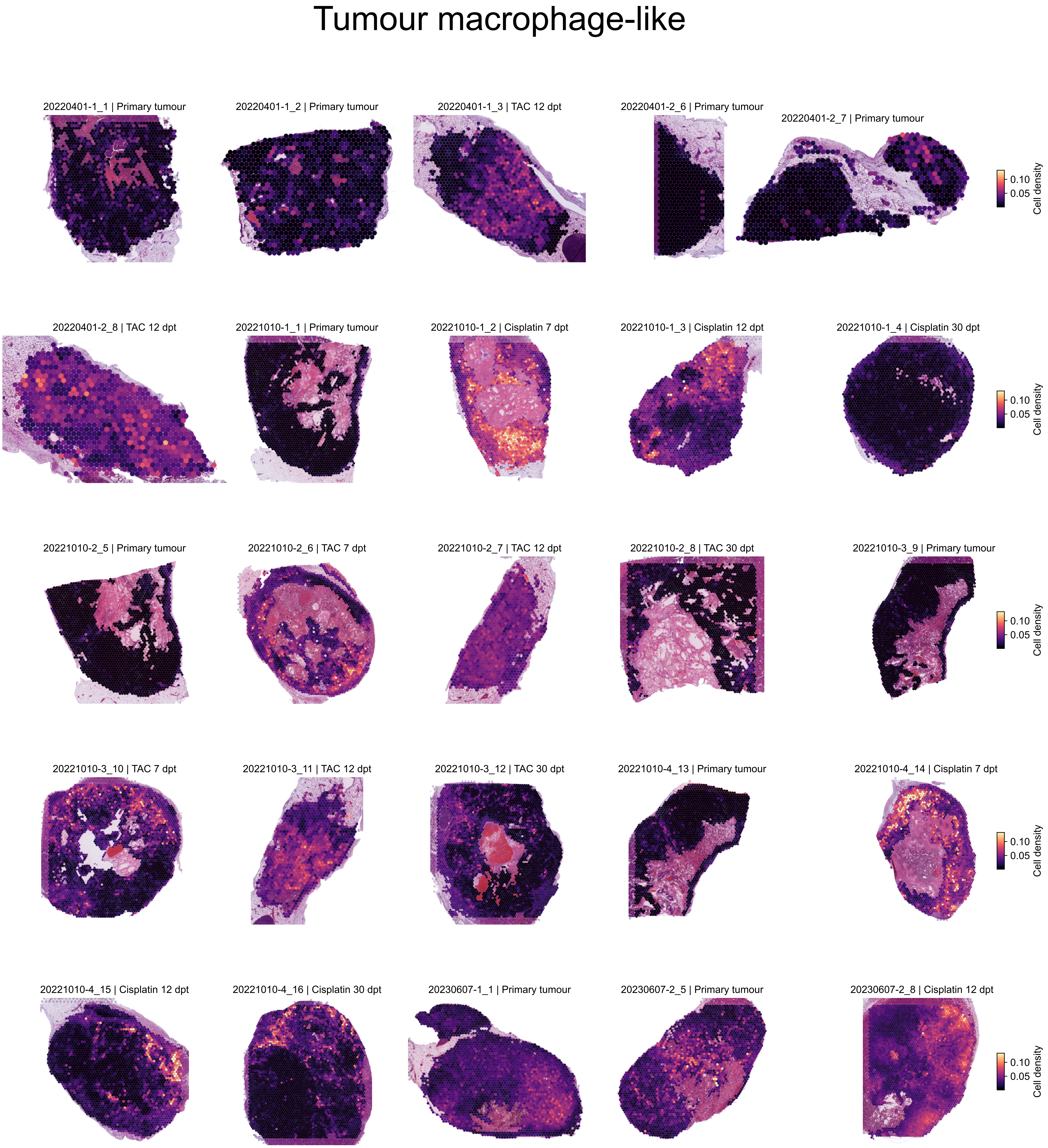

Tumour macrophage-like
20220401-1_1 | Primary tumour
20220401-1_2 | Primary tumour
20220401-1_3 | TAC 12 dpt
20220401-2_6 | Primary tumour
20220401-2_7 | Primary tumour
20220401-2_8 | TAC 12 dpt
20221010-1_1 | Primary tumour
20221010-1_2 | Cisplatin 7 dpt
20221010-1_3 | Cisplatin 12 dpt
20221010-1_4 | Cisplatin 30 dpt
20221010-2_5 | Primary tumour
20221010-2_6 | TAC 7 dpt
20221010-2_7 | TAC 12 dpt
20221010-2_8 | TAC 30 dpt
20221010-3_9 | Primary tumour
20221010-3_10 | TAC 7 dpt
20221010-3_11 | TAC 12 dpt
20221010-3_12 | TAC 30 dpt
20221010-4_13 | Primary tumour
20221010-4_14 | Cisplatin 7 dpt
20221010-4_15 | Cisplatin 12 dpt
20221010-4_16 | Cisplatin 30 dpt
20230607-1_1 | Primary tumour
20230607-2_5 | Primary tumour
20230607-2_8 | Cisplatin 12 dpt
0.10
0.05
Cell density

Tumour proliferating

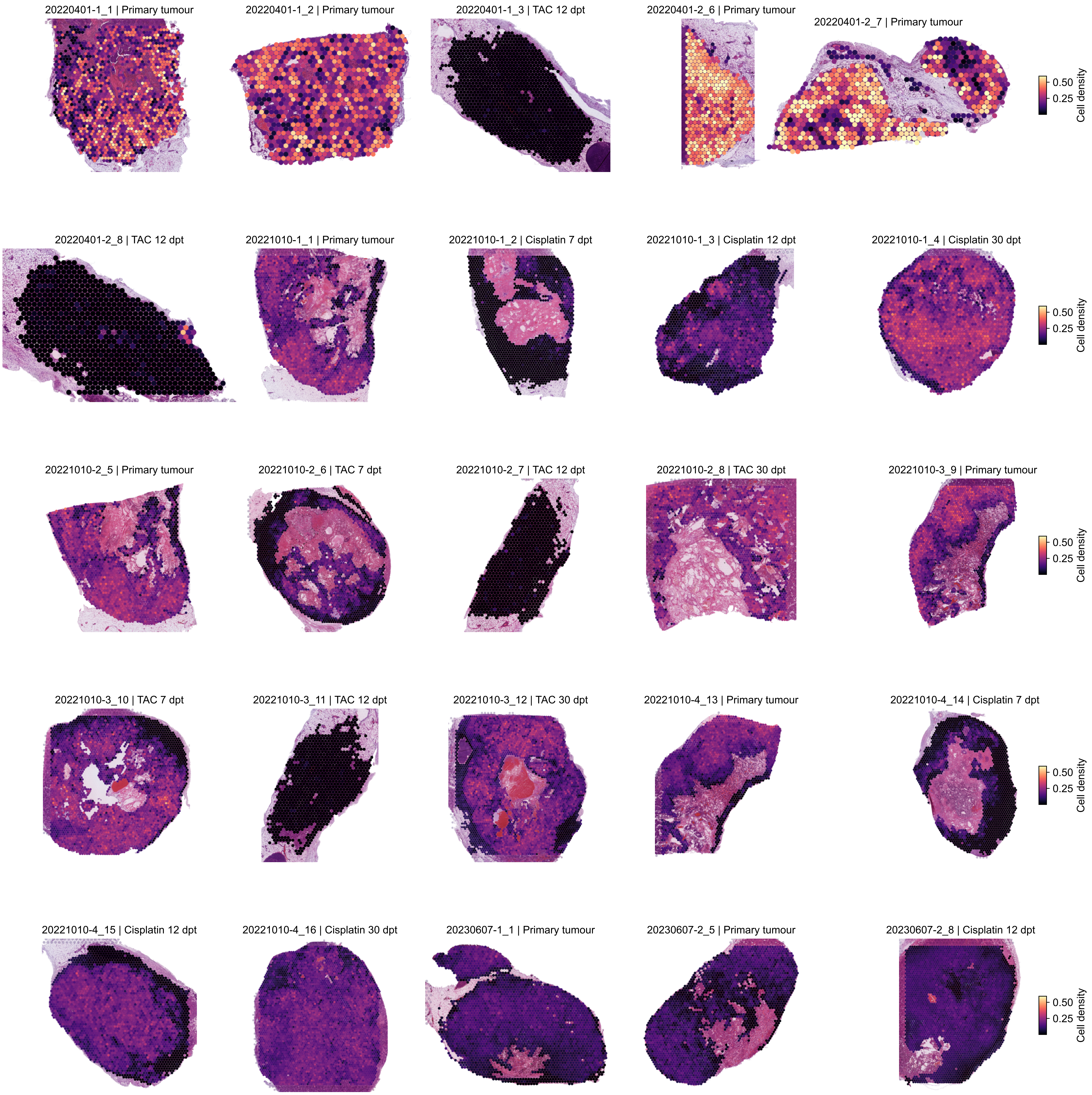

T cell regulatory

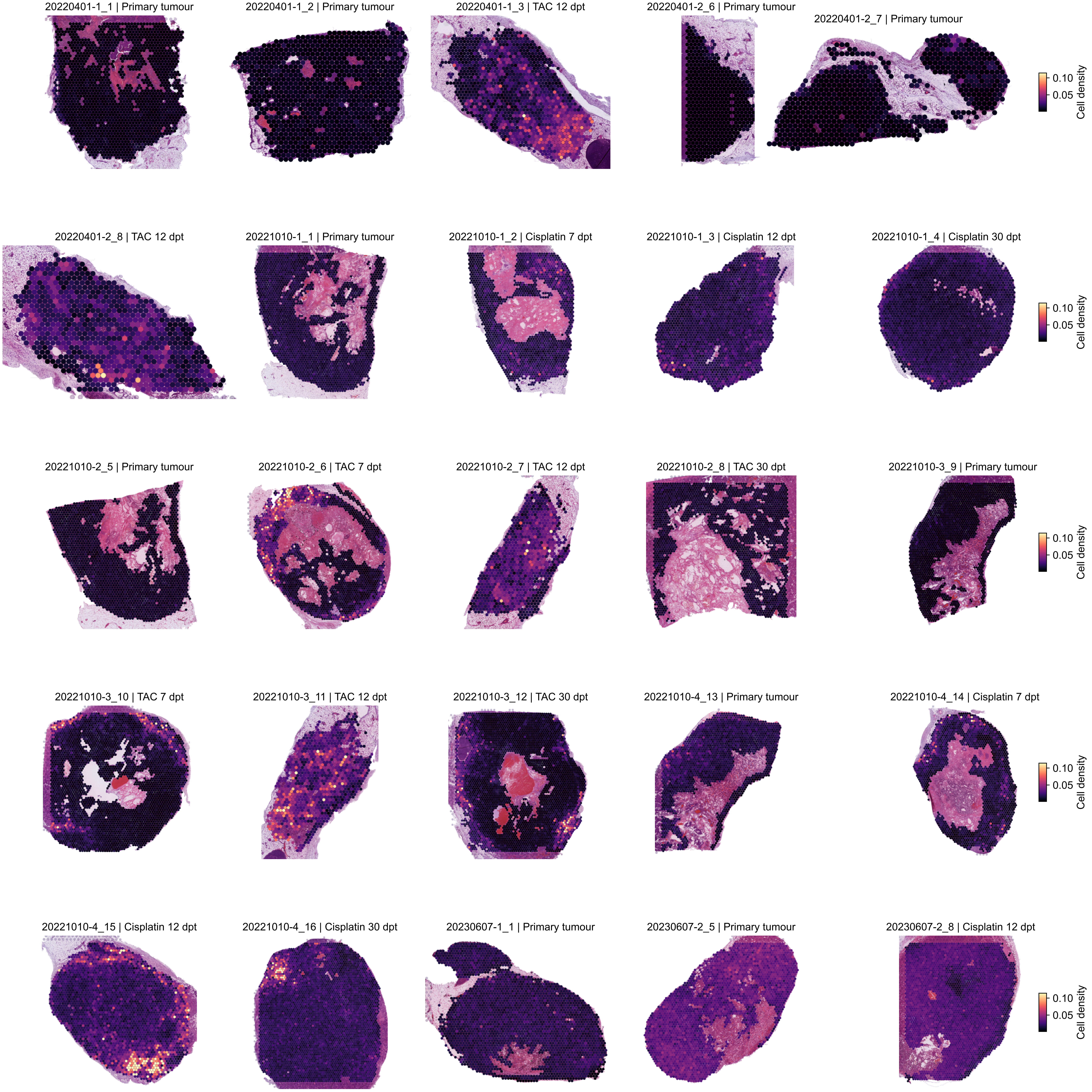

Supplement: Supplementary file 4 — Supplementary Data 2 [file 41467_2026_74125_MOESM4_ESM.pdf]
