# Supplementary material for: Spatiotemporal organisation of residual disease in mouse and human BRCA1-deficient mammary tumours and breast cancer
Source: Nat Commun. 2026 Jun 11;17:7456. doi: 10.1038/s41467-026-74125-6 (PMC13408813; doi:10.1038/s41467-026-74125-6)
Supplement: Supplementary file 18 — Reporting Summary [file 41467_2026_74125_MOESM18_ESM.pdf]

Corresponding author(s): Sven Rottenberg

Last updated by author(s): Apr 2, 2026

Reporting Summary

Nature Portfolio wishes to improve the reproducibility of the work that we publish. This form provides structure for consistency and transparency in reporting. For further information on Nature Portfolio policies, see our Editorial Policies and the Editorial Policy Checklist.

Statistics

For all statistical analyses, confirm that the following items are present in the figure legend, table legend, main text, or Methods section.

n/a	Confirmed	
[ ]	[x]	The exact sample size (*n*) for each experimental group/condition, given as a discrete number and unit of measurement
[ ]	[x]	A statement on whether measurements were taken from distinct samples or whether the same sample was measured repeatedly
[ ]	[x]	The statistical test(s) used AND whether they are one- or two-sided<br>*Only common tests should be described solely by name; describe more complex techniques in the Methods section.*
[x]	[ ]	A description of all covariates tested
[ ]	[x]	A description of any assumptions or corrections, such as tests of normality and adjustment for multiple comparisons
[ ]	[x]	A full description of the statistical parameters including central tendency (e.g. means) or other basic estimates (e.g. regression coefficient) AND variation (e.g. standard deviation) or associated estimates of uncertainty (e.g. confidence intervals)
[ ]	[x]	For null hypothesis testing, the test statistic (e.g. F, t, r) with confidence intervals, effect sizes, degrees of freedom and P value noted<br>*Give P values as exact values whenever suitable.*
[x]	[ ]	For Bayesian analysis, information on the choice of priors and Markov chain Monte Carlo settings
[x]	[ ]	For hierarchical and complex designs, identification of the appropriate level for tests and full reporting of outcomes
[ ]	[x]	Estimates of effect sizes (e.g. Cohen's *d*, Pearson's *r*), indicating how they were calculated

Our web collection on statistics for biologists contains articles on many of the points above.

Software and code

Policy information about availability of computer code

Data collection	No specific software was used for data collection.
Data analysis	Software packages used for data analysis are listed in the Methods section of the article. Furthermore, the source code is accessible on GitHub at https://github.com/rottenberglab/residual-disease and on Zenodo (doi:10.5281/zenodo.19365629). To run the provided source code, the following software environment was used:<br><br>Python 3.9.20; access 1.1.9; adjusttext 1.3.0; affine 2.4.0; aiobotocore 2.5.4; aiohappyeyeballs 2.6.1; aiohttp 3.13.3; aioitertools 0.13.0; aiosignal 1.4.0; anndata 0.10.8; archetypes 0.4.2; array-api-compat 1.11.2; asciitree 0.3.3; async-timeout 5.0.1; attrs 26.1.0; autograd 1.8.0; autograd-gamma 0.5.0; av 15.1.0; beautifulsoup4 4.14.3; botocore 1.31.17; certifi 2026.2.25; cfgv 3.4.0; charset-normalizer 3.4.6; chrysalis-st 0.2.0; click 8.1.8; click-plugins 1.1.1.2; cligj 0.7.2; cloudpickle 3.1.2; colorcet 3.1.0; contourpy 1.3.0; cycler 0.12.1; dask 2024.2.1; dask-expr 1.1.10; dask-image 2023.8.1; datashader 0.17.0; decoupler 1.8.0; deprecation 2.1.0; distlib 0.4.0; distributed 2024.2.1; docrep 0.3.2; dynaconf 3.2.13; esda 2.5.1; exceptiongroup 1.3.1; fasteners 0.20; filelock 3.19.1; fiona 1.10.1; fonttools 4.60.2; formulaic 1.2.1; frozenlist 1.8.0; fsspec 2023.6.0; geopandas 1.0.1; get-annotations 0.1.2; giddy 2.3.5; h5py 3.14.0; identify 2.6.15; idna 3.11; igraph 1.0.0; imageio 2.37.2; importlib-metadata 8.7.1; importlib-resources 6.5.2; inequality 1.0.0; inflect 7.5.0; interface-meta 1.3.0; jinja2 3.1.6; jmespath 1.1.0; joblib 1.5.3; jpype1 1.6.0; kiwisolver 1.4.7; lazy-loader 0.5; legacy-api-wrap 1.5; leidenalg 0.11.0; liana 1.2.1; libpysal 4.8.1; lifelines 0.30.0; llvmlite 0.43.0; locket 1.0.0; mapclassify 2.8.1; markdown-it-py 3.0.0; markupsafe 3.0.3; matplotlib 3.9.4; matplotlib-scalebar 0.9.0; mdurl 0.1.2; mgwr 2.2.1; mizani 0.11.4; momepy 0.6.0; more-itertools 10.8.0; mpmath 1.3.0; msgpack 1.1.2; mudata 0.2.4; multidict 6.7.1; multipledispatch 1.0.0; multiscale-spatial-image 0.11.2; narwhals 2.18.1; natsort 8.4.0; networkx 3.2.1; nodeenv 1.10.0; numba 0.60.0; numcodecs 0.12.1; numpy 1.26.4; ome-zarr 0.10.2; omnipath 1.0.12; opencv-python 4.13.0.92; packaging 26.0; pandas 2.3.3; paquo 0.9.0; param 2.2.1; partd 1.4.2; patsy 1.0.2; pillow 11.3.0; pims 0.7; platformdirs 4.4.0; plotnine 0.13.6; pointpats 2.4.0; pooch 1.9.0; pre-commit 4.3.0; propcache 0.4.1; psutil 7.2.2; pulp 3.3.0; pyarrow 21.0.0; pyct 0.6.0; pydeseq2 0.4.12; pygeos 0.14; pygments 2.19.2; pynndescent 0.6.0; pyogrio 0.11.1; pyparsing 3.3.2; pyproj

3.6.1; pysal 24.1; python-dateutil 2.9.0.post0; python-discovery 1.2.0; pytz 2026.1.post1; pywavelets 1.6.0; pyyaml 6.0.3; quantecon 0.11.1; rasterio 1.4.3; rasterstats 0.20.0; redis 7.0.1; requests 2.32.5; rich 14.3.3; rtree 1.4.1; s3fs 2023.6.0; scanpy 1.10.3; scikit-image 0.24.0; scikit-learn 1.6.1; scipy 1.13.1; seaborn 0.13.2; segregation 2.5.4; session-info 1.0.1; setuptools 69.5.1; shapely 2.0.7; simplejson 3.20.2; six 1.17.0; slicerator 1.1.0; sortedcontainers 2.4.0; soupsieve 2.8.3; spaghetti 1.7.4; spatial-image 0.3.0; spatialdata 0.1.2; spatialdata-plot 0.2.7; spglm 1.1.0; spint 1.0.7; splot 1.1.7; spopt 0.5.0; spreg 1.8.5; spvcm 0.3.0; squidpy 1.3.1; statsmodels 0.14.6; stdlib-list 0.12.0; sympy 1.14.0; tblib 3.2.2; texttable 1.7.0; threadpoolctl 3.6.0; tifffile 2024.8.30; tobler 0.12.1; toolz 1.1.0; tornado 6.5.5; tqdm 4.67.3; typeguard 4.5.1; typing-extensions 4.15.0; tzdata 2025.3; umap-learn 0.5.11; urllib3 1.26.20; validators 0.35.0; virtualenv 21.2.0; wrapt 1.17.3; xarray 2024.7.0; xarray-dataclasses 1.9.1; xarray-datatree 0.0.15; xarray-schema 0.0.3; xarray-spatial 0.5.3; yarl 1.22.0; zarr 2.18.2; zict 3.0.0; zipp 3.23.0

For manuscripts utilizing custom algorithms or software that are central to the research but not yet described in published literature, software must be made available to editors and reviewers. We strongly encourage code deposition in a community repository (e.g. GitHub). See the Nature Portfolio guidelines for submitting code & software for further information.

Data

Policy information about availability of data

All manuscripts must include a data availability statement. This statement should provide the following information, where applicable:

- Accession codes, unique identifiers, or web links for publicly available datasets
- A description of any restrictions on data availability
- For clinical datasets or third party data, please ensure that the statement adheres to our policy

All raw RNA sequencing data have been deposited in the Gene Expression Omnibus (GEO) database under accession number GSE299631 (https://www.ncbi.nlm.nih.gov/geo/query/acc.cgi?acc=GSE299631). All processed ST (10x Visium), scRNA-seq (10x Chromium), and IMC (Hyperion) data, together with the supplementary data required to reproduce the analyses presented in this study, are available through the following Zenodo repositories (ST: 15102983 (https://zenodo.org/records/15102983), scRNA-seq: 15103411 (https://zenodo.org/records/15103412), IMC co-registered with Visium: 15096025 (https://zenodo.org/records/15096026)). The deposited datasets include count matrices (Space Ranger and Cell Ranger outputs), AnnData objects containing cell type deconvolution results, tissue compartment inference, histopathological annotations, and gene set signatures, as well as raw and processed IMC acquisitions stored as SpatialData Zarr archives. Source data are provided with this paper. The remaining data are available within the Article, Supplementary Information or Source Data file.

Research involving human participants, their data, or biological material

Policy information about studies with human participants or human data. See also policy information about sex, gender (identity/presentation), and sexual orientation and race, ethnicity and racism.

Reporting on sex and gender	All five donors were female.
Reporting on race, ethnicity, or other socially relevant groupings	No information on race or ethnicity was documented.
Population characteristics	Tumor samples were collected at the time of surgery from five women carrying germline mutations of BRCA1 and treated for breast cancer. Three out of five patients received neoadjuvant chemotherapy before tumor removal.
Recruitment	Patients were treated for breast cancer and underwent genetic testing at Geneva University Hospitals, Switzerland or Centre Leon Berard, Lyon, France.
Ethics oversight	Primary human breast tissue was obtained from the Division of Clinical Pathology, Geneva University Hospitals (Geneva, Switzerland) and the Department of Pathology, Centre Léon Bérard (Lyon, France), in compliance with local ethical regulations. The study was approved by the local ethical committee of Geneva (Commission Cantonale d'éthique de la recherche Genève: CCER 2019-00004) and Lyon (Ethics Committee of Lyon Sud-Est IV and Institutional local Translational Research Review Committee: AC-2024-6625 and DC-2008-99). Tumour samples (tumourectomy) were obtained from women with germline mutations of BRCA1 with (n = 2) or without (n = 3) neoadjuvant chemotherapy. The patients provided informed consent for the use of biological samples and clinical data for research purposes. There was no compensation in accordance with local ethical and legal regulations in France and Switzerland. Participants were not specifically recruited for this study. Sex was determined based on clinical records, only females were included in this study as the research focuses on BRCA1-mutated breast cancer. No sex-based disaggregated analysis was performed due to the single-sex nature of the cohort. All donors were coded.

Note that full information on the approval of the study protocol must also be provided in the manuscript.

Field-specific reporting

Please select the one below that is the best fit for your research. If you are not sure, read the appropriate sections before making your selection.

[x] Life sciences [ ] Behavioural & social sciences [ ] Ecological, evolutionary & environmental sciences

For a reference copy of the document with all sections, see nature.com/documents/nr-reporting-summary-flat.pdf

Life sciences study design

All studies must disclose on these points even when the disclosure is negative.

Sample size	No statistical method was used to predetermine the sample size. Instead, sample size was determined based on empirical considerations and

	cost constraints. For mouse samples, we aimed for at least three tumors per condition, aligning with standard experimental designs in spatial transcriptomics. For human samples, we processed all available specimens.
Data exclusions	No samples were excluded from the analysis. In spatial transcriptomics, individual capture spots were filtered based on sample-specific quality control thresholds. Similarly, in single-cell RNA sequencing, cells were removed using the same criteria. The specific thresholds are provided in the metadata within the supplementary files, and the filtering process is detailed in the Methods section.
Replication	We generated multiple biological and technical replicates to ensure reproducibility in our spatial transcriptomics dataset. Technical and biological replicates were processed on separate Visium slides, and consistency was confirmed by comparing quality control metrics and gene expression signatures.
Randomization	During chemotherapy treatment and tumor sampling, mice were randomly assigned to different treatment groups. Human samples were not randomized due to the limited sample size and constraints related to genetic background and availability.
Blinding	Investigators were blinded during the experiment and sample processing phases but not during data analysis to ensure accurate interpretation of expression data.

Reporting for specific materials, systems and methods

We require information from authors about some types of materials, experimental systems and methods used in many studies. Here, indicate whether each material, system or method listed is relevant to your study. If you are not sure if a list item applies to your research, read the appropriate section before selecting a response.

Materials & experimental systems

n/a	Involved in the study
☐	☒ Antibodies
☒	☐ Eukaryotic cell lines
☒	☐ Palaeontology and archaeology
☐	☒ Animals and other organisms
☒	☐ Clinical data
☒	☐ Dual use research of concern
☒	☐ Plants

Methods

n/a	Involved in the study
☒	☐ ChIP-seq
☒	☐ Flow cytometry
☒	☐ MRI-based neuroimaging

Antibodies

Antibodies used	The primary antibodies used for this study were rat monoclonal anti-Galectin 3 (clone eBioM4/38 (M3/38), eBioscience/Invitrogen, Cat# 14-5301-82, RRID: AB_837132) at a 1:1000 dilution and mouse monoclonal anti-β-Actin (clone AC-15, Sigma-Aldrich, Cat# A1978, RRID: AB_476697) at a 1:1000 dilution. Horseradish peroxidase (HRP)-linked secondary antibodies were used for detection: anti-mouse IgG (Cell Signaling Technology, Cat# 7076, RRID: AB_330924) and anti-rat IgG (DAKO, Cat# P0450, RRID: AB_2617139), both used at a 1:2500 dilution.
Validation	All antibodies are commercially available and have been validated by the manufacturers for the species and applications tested. Anti-Galectin 3 (clone M3/38) is validated for use in human and mouse tissues by eBioscience, with performance confirmed in numerous peer-reviewed studies (e.g., PMID: 30674531). Anti-β-Actin (clone AC-15) is an industry-standard loading control validated across a wide range of species and applications, with extensive documentation provided by Sigma-Aldrich and supported by thousands of citations (e.g., PMID: 17296734). Secondary antibodies were validated by the manufacturers for use in Western Blotting and were tested in-house for specificity through the omission of primary antibodies to ensure no non-specific binding. Further in-house validation of primary antibodies was confirmed by observing the expected protein band sizes (30 kDa for Galectin 3 and 42 kDa for β-Actin) in the cell and tissue lysates analyzed.

Animals and other research organisms

Policy information about studies involving animals; ARRIVE guidelines recommended for reporting animal research, and Sex and Gender in Research

Laboratory animals	FVB/NJ mice were purchased from Charles River, Germany, at 6–8 weeks of age. Upon arrival at the mouse facility at Länggassstrasse 122, they underwent a 2-week acclimatization period. Following acclimatization, tumor fragments were orthotopically transplanted into the 4th mammary fat pad. Tumors were collected once they reached approximately 8 × 6 mm in size, or the mice were treated with chemotherapy. Residual and recurrent tumors were harvested 7–12 days after treatment or once they regrew to approximately 8 × 6 mm, respectively.
Wild animals	The study did not involve wild animals.
Reporting on sex	All mice used in this study were female, as breast cancer is predominantly a disease of females, with an exceptionally low incidence in males.
Field-collected samples	The study did not involve field-corrected samples.

Ethics oversight	All animal experiments were approved by the Animal Ethics Committee of the canton of Bern (BE60/2023) and are in accordance with the current Swiss Acts on Animal Experimentation.

Note that full information on the approval of the study protocol must also be provided in the manuscript.

Plants

Seed stocks	N/A
Novel plant genotypes	N/A
Authentication	N/A
