# Supplementary material for: Spatiotemporal organisation of residual disease in mouse and human BRCA1-deficient mammary tumours and breast cancer
Source: Nat Commun. 2026 Jun 11;17:7456. doi: 10.1038/s41467-026-74125-6 (PMC13408813; doi:10.1038/s41467-026-74125-6)
Supplement: Supplementary file 19 — Transparent Peer Review file [file 41467_2026_74125_MOESM19_ESM.pdf]

nature portfolio

Peer Review File

Spatiotemporal organisation of residual disease in mouse and human BRCA1-deficient mammary tumours and breast cancer

Corresponding Author: Professor Sven Rottenberg

This file contains all reviewer reports in order by version, followed by all author rebuttals in order by version.

Version 0:

Reviewer comments:

Reviewer #1

(Remarks to the Author)
Turos et al. submitted a manuscript titled: Spatiotemporal organisation of residual disease in mouse and human BRCA1-deficient mammary tumours and breast cancer" to be considered for publication in Nature Communications.
The manuscript is overall very well written. The data presented are convincing and nicely support the claims given in the manuscript.
This said, I would have only one question – Figure 2c, under "Primary", the two tissue sections (cisplatin and TAC threated) look very much the same, only their spatial orientation is slightly different. Why is this? It looks like it is the same image - please double check that the figure is correct.

(Remarks on code availability)

Reviewer #2

(Remarks to the Author)
Turos et al present an interesting study describing spatiotemporal patterns of therapeutic resistance in BRCA1;p53-deficient mice and human samples through a complex longitudinal integration of scRNA-seq, spatial transcriptomics and and imaging mass cytometry. This is a beautiful and data rich study tracking mice as they acquire resistance to chemotherapy, profiled before treatment, at the MRD and recurrence time points. The focus is on describing what drives MRD, and the authors find a quiescent EMT niche programme linked with MRD that persists upon therapy, can be recapitulated in human tumours and is associated with worse prognosis. While the finding of a quiescent EMT niche being linked with resistance to therapy and worse survival is not surprising, with other studies reporting similar findings (e.g. PMID 32503922, 38999963), the strength of the manuscript consists in the rich profiling of developing resistance in a bespoke mouse model, which constitutes a highly valuable resource. In this respect, I appreciate the authors have made the processed data and code readily available to the community. The cisplatin uptake experiment is also novel and yields informative results. The analyses appear robust and conducted to the highest standards in the field. I have the following questions/comments which I believe would improve the manuscript:

1. The authors have largely focused on the tumour cells, with the purpose of identifying those cells that drive MRD. They loosely define proliferative, hypoxic, EMT and other niches or compartments, but those compartments must contain more than the tumour cells. In this respect, it is not entirely clear how immune and stromal cells within these compartments might influence the state of the cancer cells. Cell-cell communication and ligand-receptor analyses would be helpful to shed light on this.

2. A brief analysis of SASP expression is performed in fig 3, alluding to a senescent phenotype linked with residual disease – but this does not coincide with the EMT niches. What is it that is more likely to drive MRD then – senescence or a quiescent EMT state? Is it still not the same tumour cells? The SASP is elevated in the TME, as would be expected, as it is secreted. How can the authors assess that these signals are not originating from the tumour cells?

3. The CNV analysis is performed across all compartments, but CNVs should only be called in the tumour cells.

4. Figure 1c/d: immune and EMT cells are described to be enriched in the residual tumour – can this be quantified? Also, residual downregulated genes are described in a confusing manner (inconsistent, either describes what the gene does or what the effect of downregulation is – one or the other?).

5. Figure 3: when describing pathways it is a bit confusing how they list the pathways as they are not all upregulated: "Similar trends were observed for p53,EGFR, TGFb, and JAK-STAT pathways."

6. Figure 4a: why is cisplatin uptake higher at 24hrs?

7. Figure 4j is not referenced in the text.

8. Figure 5e: compartment similarities between human and mouse are shown, but it is difficult to quantify how similar overall these are. Why are there so many more compartments in human versus mouse? Although I understand this is derived based on the elbow method of the Chrysalis tool, this does not seem biologically intuitive. Could a reduced number of human compartments explain the data similarly well and be more in line with what we see in mouse? If not, one could argue that this mouse model is not a good model for human tumours, at least not in this setting.

9. Figure 5f: is there a more quantitative way of showing EMT remains similar across primary and residual?

10. The link between EMT and worse survival is not surprising and it's been widely reported before. It is not clear why the authors chose an EMT-high/low split in the SCAN-B cohort but an EMT+proliferation status split in the TCGA cohort. Furthermore, associations with survival are shown, but what would be more relevant is if the signature proposed by the authors would be linked with recurrence.

11. Several sections, most prominently "Chemotherapy-induced changes in the transcriptional tissue landscape of MRD", list a myriad of genes whose expression has led to identification of the cellular compartments. This made up a lengthy part of the results section, which is rather tedious to follow, while interesting findings, such as the dynamic reorganisation of compartments post-chemotherapy, are concisely described. Shortening the gene descriptions and highlighting just a handful of key genes (perhaps supplemented by a lengthier table) would enhance the readability of the manuscript.

12. "These findings, validated across two different therapeutic strategies, suggest that the primary mechanisms of drug tolerance may be drug-independent." – this conclusion may be overstretched, as only two drugs have been tried in mouse and one in human, and mutation accumulation has not been investigated in this context. I would tone down.
13. The authors discuss a "permissive TME" that enables drug resistance, which of course makes a lot of sense – however, it is not clear to me from the study what exactly this permissive TME is.

14. Sometimes, the depictions/arrangement in the figure panels are puzzling:
- Figure 1a/b: confusing order of schematics, and volume is both here and in b. Figure 1b is rather difficult to follow, and the time course not clear. It could benefit from a better structure and more ample caption to describe what is shown.

- Figure 1i does not really seem to belong to Figure 1, as it is not really linked to any of the previous panels
- Figure 2a – there are too many colours, arrows and labels. A better clarification of cluster number/colour/label is needed. Why highlight key genes both here and in b?
- Figure 2c: it is difficult to match the colours to the niche.
- Figure 2e: it is not obvious at which time point MRD occurs.
- Figure 2f shows the cell type contribution to the defined tissue compartments – it would make much more sense to define this from the start, i.e. after panel 2b, and then explore differences after treatment (panels d-e, which are the key points you want to leave the readers with).
- Throughout figure 2 it is difficult to follow when the authors refer to numbered compartments rather than biological niches – it would be beneficial to assign all compartments a biological meaning (e.g. proliferative, EMT, hypoxic, rather than compartment 5, 2, 10) and use those labels throughout the downstream analysis. I believe the authors have even confused themselves, when referring in the text to "proliferating (0)" but in the figure it is compartment 5.
- Figure 4a: cisplatin treatments are not in order, would make sense to show time course.

(Remarks on code availability)

Reviewer #3

(Remarks to the Author)
In this manuscript the authors aim to characterize the changes post-chemotherapy in the Brca1-/-;Trp53-/- mouse model using multimodal analysis. Through a combination of single-cell RNA-seq, spatial transcriptomics (ST), and imaging mass cytometry (IMC), the authors delineate the dynamic spatial and temporal characteristics of residual tumor cells and their microenvironment after chemotherapy. They find that an EMT-like tumor cell population that persists post-treatment and

appears to be a critical reservoir for recurrence. They show that this EMT program is conserved in human BRCA1-deficient breast cancers and correlates with poor prognosis, making it a potential therapeutic target. Overall the study is comprehensive but largely descriptive. It unclear if there are novel insights gained through the study as EMT has been previously attributed to chemoresistance. However, the level of granularity with which the study has been conducted will provide a good resource to the breast cancer community. Below are a few points that the reviewer feels need clarification.

1. In Fig 1c the authors should provide a dot plot of the marker genes used to classify the different populations. In particular how is the LA-EMT cluster defined?
2. The authors should use other tools such as stem id, CytoTRACE to understand the Luminal-alveolar to LA-EMT cluster is an undifferentiated cluster. Where is this cluster located in the spatial analysis? In the rest of the paper, when the authors refer to EMT, does it mean it is the LA-EMT cells?
3. Why are clusters 7 and 8 omitted from the RNA-velocity analysis?
4. Are the changes seen in the pseudobulk of single cells (Fig 1d) and pseudobulk of spatial (Fig. 1h) overlapping? If so, what percentage? Also, the supplementary table (ST1 and ST2) should be replaced with gene names rather than gene symbols/ids?
5. "In the recurrent stage, however, tumour architecture and cell morphology resembled those of the primary tumours once again." – this sentence needs a figure reference
6. "Hypoxic tumour cell populations were predominantly located in close proximity to these necrotic cores." Authors should delineate the hypoxic cores in the figures.
7. It is unclear if the EMT-tumor niche (even a small minority) is present before treatment or emerges post-treatment.
8. The paper in general is very dense to read and it is hard to easily evaluate the supplementary spatial transcriptomic data. Maybe the authors can come up with more summarized figures.

(Remarks on code availability)

Reviewer #4

(Remarks to the Author)
In the manuscript entitled "Spatiotemporal organisation of residual disease in mouse and human BRCA1-deficient mammary tumours and breast cancer," Turos et al. combine single-cell RNA sequencing, spatial transcriptomics, and imaging mass cytometry of BRCA1;p53-deficient mouse mammary tumors to characterize the dynamics of specific tumor cell subpopulations and their surrounding microenvironments during minimal residual disease (MRD) and recurrence. Overall, this study integrates single-cell and spatial omics approaches to study the BRCA1;p53-deficient mouse mammary tumors and their response to standard therapies. The authors also validated some gene signatures in patient samples. While the conclusions the authors are able to draw from the omic data are largely based on and consistent with published literatures, the study is largely correlative and descriptive without performing functional validations of any cell types or signaling pathways for their roles in tumor growth and/or responses to therapies. Therefore, this study provides an omic resource of this BRCA1;p53-deficient mouse mammary tumor model, but not a research article.

Specific points:

1. The study is descriptive. The authors do not provide any functional validation of the relevance of the identified tumor or stromal (e.g., macrophage) populations in MRD and recurrence. The manuscript would be more compelling if the authors attempted to functionally assess the role of some of these populations.

2. Suppl. Figure 2 is intended to support the data presented in Fig. 1F. The authors report differences in the presence of EMT cells and immune cell infiltrates (such as macrophages) based on the spatial transcriptomics data. However, Suppl. Fig. 2 includes only a single representative image, which limits interpretation. In this figure, the authors display stromal populations only in treatment-naïve tumor, but do not show how these populations appear in the residual tumor (except for macrophages, which are shown in panel D). On the opposite, they present tumor populations in residual tumors, but not in treatment-naïve primary tumors.

3. Suppl. Figure 3 is difficult to interpret due to disorganized sample nomenclature and treatment time points. It would be helpful if the authors provided a quantification of the percentage of the different areas represented in the figure. Additionally, are there observable differences in necrotic areas between treatment-naïve and residual tumors?

4. Graphs in Fig. 1F, Suppl. 4C and D, and Fig. 2E lack statistical validation.

5. In figure 2C, the authors investigated tumor evolution at four time points: pre-treatment, MRD at 7 and 12 days post-treatment, and recurrent tumor, in both TAC- and cisplatin-treated samples. In the manuscript, the authors state that MIP visualizations revealed major shifts in niche composition: non-treated tumors were dominated by the proliferative niche (5), fibrotic stroma (4), and hypoxic regions (10), while EMT niches were scattered. In MRD, compartments 11 and 12 became predominant, indicating immune infiltration and macrophage activity. Could the authors highlight these changes more clearly in Figure 2C, for example by labelling the relevant compartments, to aid interpretation?

(Remarks on code availability)
N/A

Reviewer #5

(Remarks to the Author)
The topic presented in this manuscript is both relevant and interesting, using a variety of imaging techniques as well as spatial transcriptomics (ST) and imaging mass cytometry (IMC).
The introduction provides an adequate but not excessive background on Triple Negative Breast Cancer and the reasoning behind the analyses. Special emphasis is given to the topic of Minimal Resistant Disease (MRD), a key aspect of the cancer biology subject of the paper.
The results section provides a thorough description of the exhaustive analyses conducted for this manuscript. The reviewers provide myriad figures which attempts to capture the experimental results in an encyclopedic fashion. Occasionally, we have found that it is difficult to see the actual described results within the figures. Specific examples are detailed below.
The discussion section is relatively short and lacks many strong statements regarding any patterns observed in the very large number of analyses performed within the manuscript. The most interesting trends observed are that the EMT niche appears to play a central role in tumor survival through MRD and the reinforcement of the observation that drug uptake in tumor cells occurs independent of the cellular population. Although it would be beneficial if more trends could be extracted from the large amount of data, this is not strictly necessary. We would have liked to see more of an explanation as to why certain isotopes of platinum may have proved better for analyses than other isotopes.
The methods section is thorough and provides adequate detail as to how independent investigators might replicate the study. The investigators have provided adequate detail for how biological samples were obtained, how spatial transcriptomics was conducted, and how IMC analyses were conducted. The authors have also detailed their computational methods for cell phenotyping, transcriptional analyses, cellular classification, IMC analyses, and cellular niche assignments with code that has been uploaded for common use.
Overall, the manuscript provides more description of the biology than explanation of the biology, but we believe that the thoroughness of the analyses and the important subject of experimentation merits inclusion within the journal. The quantity of data and analyses in this carefully written paper are immense and powerful in their own right.

Proposed edits:
• Please elaborate further on possible causes of 198Pt saturation in IMC detection. Do control samples demonstrate an unusual 198Pt signal? Could the cisplatin sample be tested by Mass Spec to find any unexpected distribution of Pt isotopes?
• Figure 4j was not referenced in the text. Was this intentional?
• Supplementary figure 21c was referenced in the text of the paper but does not exist in the supplementary figures

(Remarks on code availability)

Version 1:

Reviewer comments:

Reviewer #2

(Remarks to the Author)
The authors have addressed my comments adequately and the manuscript is much improved as a result. I have no further concerns and congratulate the authors for this study which will be a valuable resource for the community.

(Remarks on code availability)
The code is well structured and the repository contains the minimal details required to understand the purpose of each script. I have tested parts of the code and they worked.

Reviewer #3

(Remarks to the Author)
The authors have satisfactorily addressed all my comments. I have no further comments.

(Remarks on code availability)

Reviewer #4

(Remarks to the Author)
This revised manuscript remians largely descriptive and correlative. The added Galectin 3 knockdown data are consistent with previous publications, such as DOI: 10.1158/2159-8290.CD-17-0539, but do not gain new understanding of Galectin 3 beyond what's published. This work could be a good resource as the start of a study, but do not contribute new insights to breast cancer residual disease research and treatment at this stage.

(Remarks on code availability)

Reviewer #5

(Remarks to the Author)
The author has addressed my concerns in the most recent version of the manuscript.
No further revisions requested.

(Remarks on code availability)

Open Access This Peer Review File is licensed under a Creative Commons Attribution 4.0 International License, which permits use, sharing, adaptation, distribution and reproduction in any medium or format, as long as you give appropriate credit to the original author(s) and the source, provide a link to the Creative Commons license, and indicate if changes were made.
In cases where reviewers are anonymous, credit should be given to 'Anonymous Referee' and the source.
The images or other third party material in this Peer Review File are included in the article's Creative Commons license, unless indicated otherwise in a credit line to the material. If material is not included in the article's Creative Commons license and your intended use is not permitted by statutory regulation or exceeds the permitted use, you will need to obtain permission directly from the copyright holder.
To view a copy of this license, visit https://creativecommons.org/licenses/by/4.0/

Response to Reviewers

We sincerely thank the reviewers for their time and for providing insightful feedback on our manuscript. Their thorough and constructive comments have substantially helped to enhance the quality and clarity of the manuscript.

Based on the reviewers' feedback, we have significantly strengthened the study with functional and clinical validation. The major points of this revision include:

1. Functional validation of the EMT niche**.** We performed new functional experiments using a KB1P residual tumoroid model.
2. Expanded clinical analysis of recurrence. We incorporated an additional large-scale clinical cohort (METABRIC) to assess the EMT signature in the context of disease recurrence.
3. Enhanced clarity. We streamlined the manuscript by introducing biological annotations for all spatial compartments (**Table 1** and **Table 2**) and redesigning complex figure panels (e.g. **Fig.1** and **Fig.2**). We also addressed technical queries regarding SASP cellular origins, CNV baselining, and IMC isotopic quantification.

We also addressed technical queries regarding SASP cellular origins, CNV baselining, and IMC isotopic quantification.

We have carefully addressed all points raised and believe that these revisions have significantly improved the clarity and robustness of our work. We thank the reviewers again for their valuable contributions.

Reviewer comments are presented in grey boxes, and our responses are provided in plain text throughout the response letter.

> *Reviewer #1 (Remarks to the Author)*
> *Turos et al. submitted a manuscript titled: Spatiotemporal organisation of residual disease in mouse and human BRCA1-deficient mammary tumours and breast cancer" to be considered for publication in Nature Communications.*
> *The manuscript is overall very well written. The data presented are convincing and nicely support the claims given in the manuscript.*
> *This said, I would have only one question – Figure 2c, under "Primary", the two tissue sections (cisplatin and TAC treated) look very much the same, only their spatial orientation is slightly different. Why is this? It looks like it is the same image - please double check that the figure is correct.*

We thank the reviewer for their positive assessment of our manuscript and for noting that the data are convincing and support our claims.

We thank the reviewer for pointing out the similarity between the two samples used to demonstrate the heterogeneity of the primary tumours in **Fig.2c**. The two samples in question were biological replicates sectioned from the same tumour block. To ensure clarity and avoid potential confusion, we have replaced one of these samples with a different specimen in the updated figure.

> *Reviewer #2 (Remarks to the Author)*

Turos et al present an interesting study describing spatiotemporal patterns of therapeutic resistance in BRCA1;p53-deficient mice and human samples through a complex longitudinal integration of scRNA-seq, spatial transcriptomics and imaging mass cytometry. This is a beautiful and data rich study tracking mice as they acquire resistance to chemotherapy, profiled before treatment, at the MRD and recurrence time points. The focus is on describing what drives MRD, and the authors find a quiescent EMT niche programme linked with MRD that persists upon therapy, can be recapitulated in human tumours and is associated with worse prognosis. While the finding of a quiescent EMT niche being linked with resistance to therapy and worse survival is not surprising, with other studies reporting similar findings (e.g. PMID 32503922, 38999963), the strength of the manuscript consists in the rich profiling of developing resistance in a bespoke mouse model, which constitutes a highly valuable resource. In this respect, I appreciate the authors have made the processed data and code readily available to the community. The cisplatin uptake experiment is also novel and yields informative results. The analyses appear robust and conducted to the highest standards in the field.

We thank the reviewer for this very positive and thoughtful assessment of our work, and for recognising both the value of the longitudinal, multimodal profiling strategy and the utility of the KB1P mouse model as a resource for the community. We appreciate the reviewer's acknowledgement of the robustness of the analyses and the novelty of the cisplatin uptake experiments. We also agree with the reviewer that quiescent EMT-like programmes have previously been linked to therapeutic resistance and adverse outcome, as reported for example by studies such as PMID: 32503922 and PMID: 38999963. In the revised manuscript, we now explicitly cite these works and further clarify how our study builds on this literature by providing a spatiotemporal and spatially resolved view of how such EMT-associated, drug-tolerant states emerge, persist during MRD, and revert upon recurrence, both in a controlled *in vivo* model and in human tumours.

Included section in the discussion:

"*While EMT-associated and quiescent drug-tolerant states have been previously linked to therapeutic resistance and adverse prognosis[97–99], our study extends these observations by resolving their spatial organisation, temporal stability, and reversibility during minimal residual disease using longitudinal multimodal profiling.*"

I have the following questions/comments which I believe would improve the manuscript:

1. The authors have largely focused on the tumour cells, with the purpose of identifying those cells that drive MRD. They loosely define proliferative, hypoxic, EMT and other niches or compartments, but those compartments must contain more than the tumour cells. In this respect, it is not entirely clear how immune and stromal cells within these compartments might influence the state of the cancer cells. Cell-cell communication and ligand-receptor analyses would be helpful to shed light on this.

We thank the reviewer for their thorough assessment of the manuscript and for this suggestion. To investigate cell-cell interactions across cellular niches, we performed ligand-receptor analysis with LIANA+, a recently developed cell communication framework. This analysis provided additional insight into the cellular composition and functional characteristics to the identified niches. The corresponding results have been added to the **Functional characterisation of MRD** subsection of the **Results** and to **Supplementary Fig.21**.

Included section in the main text:

"To characterise cell-cell communication networks within the identified cellular niches, we performed ligand-receptor interaction analysis using LIANA+[74], identifying highly correlated signalling axes for

*each niche (**Supplementary Fig.21**). The proliferative niche (5) was characterised by collagen-Ddr1 and Igf1-integrin signalling, emphasizing a growth-promoting environment[75,76]. Conversely, the EMT-associated niche (2) utilised Wnt7b-Fzd1/Lrp6 pathways, facilitating cellular plasticity and migration[77]. Furthermore, Calml3-Kcnq1 interaction was found to be highly associated with this niche, revealing the role of the potassium channel Kcnq1, which has been implicated in disrupting epithelial homeostasis[78]. In contrast, the hypoxic niche (10) exhibited high enrichment for the Vegfa-Cd44 axis, suggesting a coordinated programme for metabolic adaptation and survival under stress[79]."*

> *2. A brief analysis of SASP expression is performed in fig 3, alluding to a senescent phenotype linked with residual disease – but this does not coincide with the EMT niches. What is it that is more likely to drive MRD then – senescence or a quiescent EMT state? Is it still not the same tumour cells? The SASP is elevated in the TME, as would be expected, as it is secreted. How can the authors assess that these signals are not originating from the tumour cells?*

Our data suggest that the elevated SASP expression signature is primarily associated with the TME. As reported in recent literature [1], CAFs and other stromal cells express higher levels of SASP factors, creating a permissive environment for tumour cells, including the quiescent EMT phenotype described here. We corroborated this association by assessing the Pearson correlation coefficient between the SASP gene expression signature and the tissue compartment scores associated with the cellular niches, as reported in **Fig.3**.

To further confirm this, we investigated the SASP signature in the scRNA-seq data (**Fig.R1a**), where it showed a lower expression signature in tumour cells compared to endothelial cells and fibroblasts. Finally, we assessed the SASP signature in the spatial data relative to the tumour fraction inferred with cell type deconvolution, confirming a strong negative correlation with tumour cells found in situ (**Fig.R1b**).

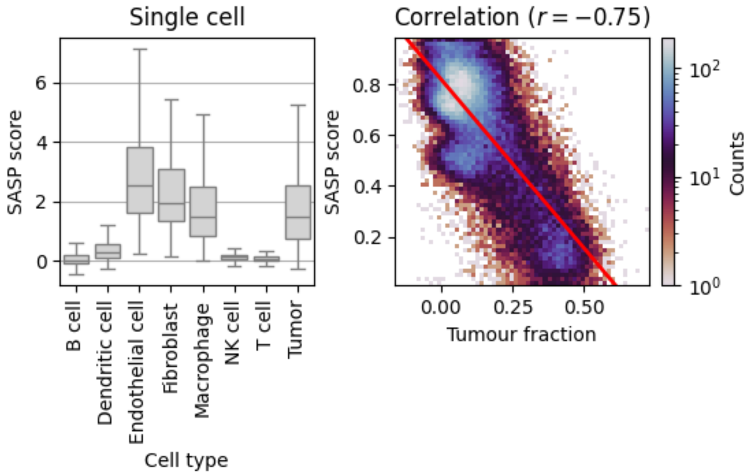

Fig.R1 | SASP signature distribution across cell types in the scRNA-seq dataset (left panel). Correlation between the SASP score and tumour fraction in the ST dataset at the capture spot level (right panel).

> *3. The CNV analysis is performed across all compartments, but CNVs should only be called in the tumour cells.*

We thank the reviewer for this observation. To infer copy number variations (CNV) across the tumour samples, we utilised histopathological annotations to restrict calculations to tumour-containing capture spots (**Supplementary Fig.5**). CNV inference was performed by defining the surrounding stroma and

healthy mammary gland samples as a reference baseline and calling CNV events at the level of Visium capture spots.

As 10x Visium does not resolve individual cells, we acknowledge that infiltrating stromal cells may partially influence the inferred CNV profiles. To investigate this potential effect, we performed CNV analysis on the single-cell data, which confirmed that the identified CNV patterns are consistent and reflect the genetic alterations of the tumour cells *in situ* (**Fig.R2**).

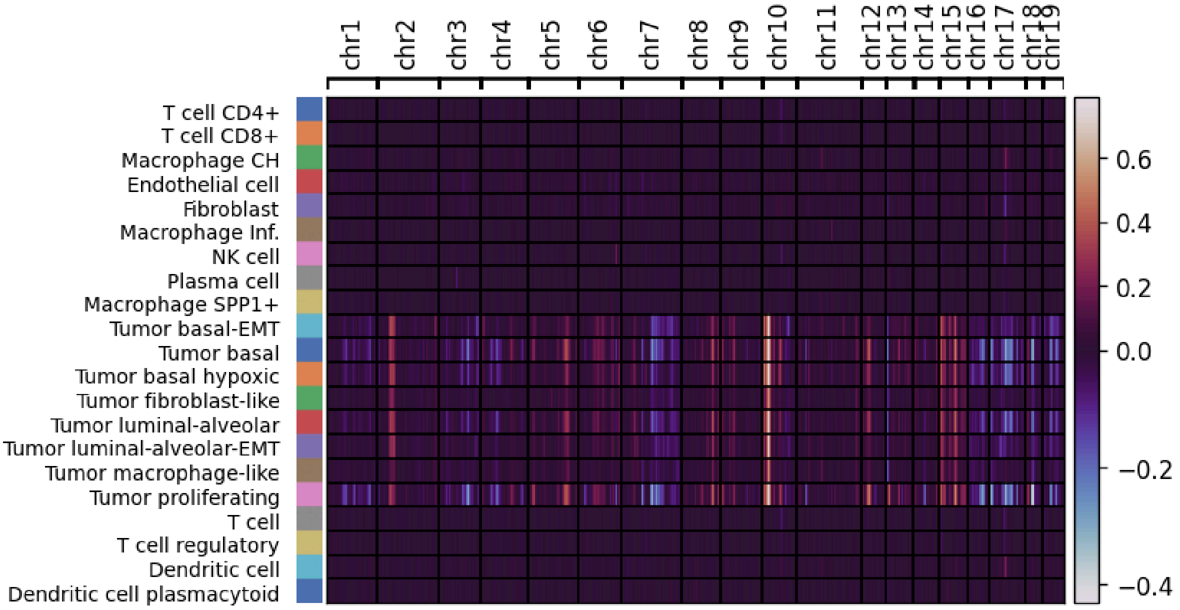

Fig.R2 | CNV profiles inferred from scRNA-seq data across different cell types.

> *4. Figure 1c/d: immune and EMT cells are described to be enriched in the residual tumour – can this be quantified? Also, residual downregulated genes are described in a confusing manner (inconsistent, either describes what the gene does or what the effect of downregulation is – one or the other?).*

We thank the reviewer for this suggestion. Our intention was to direct the reader's attention to cell type enrichment *in situ*, as it provides a more accurate representation of cellular composition within the tumour. These results are detailed now in **Fig.1g** and **Supplementary Fig.6**. Nevertheless, we have added a cell number-based overview (**Fig.R3 and Supplementary Fig.3**) using the single-cell data to provide further quantification.

In addition, we have revised the corresponding paragraph to ensure a consistent description based on the biological functions of the highlighted genes. To further improve clarity, we have added a table to the main text detailing these genes and their associated functions (**Table 1**).

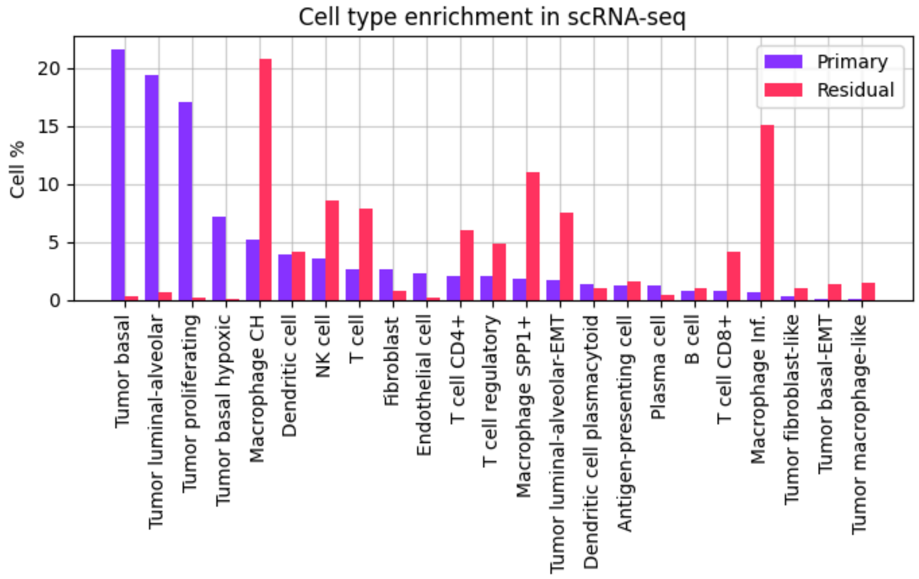

Fig.R3 | Percent-based changes in cell type composition in primary and residual tumours of the scRNA-seq data.

> *5. Figure 3: when describing pathways it is a bit confusing how they list the pathways as they are not all upregulated: "Similar trends were observed for p53,EGFR, TGFb, and JAK-STAT pathways."*

We have clarified the text by separating the p53, JAK-STAT pathways from the EGFR and TGFb pathways, which display similar trends within their respective groups during tumour progression.

Included section in the main text:

"*In primary tumours, we observed strong downregulation of the Trail apoptotic pathway, which partially recovered at the time of maximal treatment response but was further reduced in recurrent tumours. When inhibited, the Trail apoptotic pathway has been implicated in tumour initiation and progression*[64]*. Similar temporal trends were detected for p53 and JAK-STAT pathways, while the activity of EGFR and TGF-β pathways were increased. While JAK-STAT activation has been linked to early cancer progression, in our data it was predominantly induced during MRD*[65]*. In contrast, the MAPK signalling pathway was significantly downregulated during MRD, consistent with the diminishing proliferating tumour cell population.*"

> *6. Figure 4a: why is cisplatin uptake higher at 24hrs?*

Cisplatin uptake at 24 hours is lower than at 4 hours, as indicated by the reduced raw platinum (Pt) signal intensities in **Fig.4c** and by the corresponding kernel density plots in **Fig.4d**.

> *7. Figure 4j is not referenced in the text.*

We thank the reviewer for this observation. As part of a major restructuring to improve the clarity and flow of the manuscript, we have moved **Fig.4j** to the supplementary materials (**Supplementary Fig.27**).

8. Figure 5e: compartment similarities between human and mouse are shown, but it is difficult to quantify how similar overall these are. Why are there so many more compartments in human versus mouse? Although I understand this is derived based on the elbow method of the Chrysalis tool, this does not seem biologically intuitive. Could a reduced number of human compartments explain the data similarly well and be more in line with what we see in mouse? If not, one could argue that this mouse model is not a good model for human tumours, at least not in this setting.

We thank the reviewer for drawing attention to this point. The higher number of compartments identified in the human dataset reflects the inherent interpatient heterogeneity characteristic of clinical samples. Human tumours follow more diverse developmental trajectories, whereas our mouse model utilizes distinct tumours within a controlled genetic background to minimize extraneous variance.

While the total number of compartments is greater in the human dataset, we observed strong associations (cosine similarity > 0.5) between human compartments and the key tumour niches identified in our mouse model. This includes the EMT and proliferating niches, as well as TME-related compartments such as fibrotic and lymphocyte-infiltrated niches. This high degree of conservation in core functional niches supports the validity of the mouse model in this setting, despite the broader complexity of human samples.

9. Figure 5f: is there a more quantitative way of showing EMT remains similar across primary and residual?

We thank the reviewer for this question. The purpose of **Fig.5f** (now **Fig.5g**) was to illustrate the spatial dependency of the EMT expression program in human clinical samples and its association with lesions annotated on the histology. To provide a quantitative assessment, we analysed the difference between signatures across primary and residual tumours resulting in statistically significant differences as shown in **Fig.5h**.

10. The link between EMT and worse survival is not surprising and it's been widely reported before. It is not clear why the authors chose an EMT-high/low split in the SCAN-B cohort but an EMT+proliferation status split in the TCGA cohort. Furthermore, associations with survival are shown, but what would be more relevant is if the signature proposed by the authors would be linked with recurrence.

We thank the reviewer for raising this point. As the TCGA and SCAN-B cohorts do not contain longitudinal recurrence data, we have now included an additional large-scale clinical cohort, METABRIC[2], to address this. Our original intention with the TCGA analysis was to illustrate the potentially opposing effects of proliferative and EMT signatures on patient survival. We have clarified this in the text and moved the TCGA results to the supplementary materials due to the smaller cohort size.

Included section in the main text:

"*First, we evaluated the association between EMT signature expression and patient outcome in TNBC patients from the Sweden Cancerome Analysis Network - Breast (SCAN-B) cohort[90] (n = 315) (**Fig.5i**). Patients were stratified into EMT^{High} and EMT^{Low} groups based on EMT signature expression. Kaplan-Meier analysis revealed significantly poorer overall survival for EMT^{High} patients compared to EMT^{Low} patients, as assessed by the log-rank test.*

To further validate the prognostic relevance of the EMT programme in an independent cohort, we analysed TNBC patients from the METABRIC dataset (n = 319)[91], focusing on 5-year recurrence-free survival (RFS). Consistent with the SCAN-B results, EMT^{Low} patients exhibited significantly improved

*RFS compared to EMT^{High} patients (log-rank χ^2 = 0.0171) (**Fig.5j**), supporting a link between elevated EMT signature expression and adverse clinical outcome. An additional analysis using the Cancer Genome Atlas Breast Invasive Carcinoma Collection (TCGA-BRCA)[92] cohort (n = 53), incorporating both EMT and proliferating signatures, yielded concordant results (**Supplementary Fig.30**).*"

Furthermore, we have now unified our stratification strategy (using median expression) across both the SCAN-B and METABRIC cohorts. Crucially, we assessed 5-year recurrence-free survival in METABRIC (**Fig.R4**), observing significantly worse outcomes for patients with high EMT signatures. These results are now reported in **Fig.5j**.

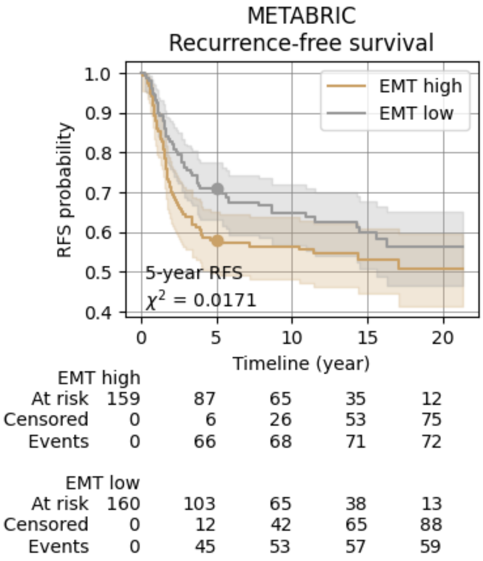

Fig.R4 | 5-year recurrence-free survival analysis of the METABRIC cohort shows significantly worse outcomes for patients with high EMT signatures. Now reported in **Fig.5j.**

> *11. Several sections, most prominently "Chemotherapy-induced changes in the transcriptional tissue landscape of MRD", list a myriad of genes whose expression has led to identification of the cellular compartments. This made up a lengthy part of the results section, which is rather tedious to follow, while interesting findings, such as the dynamic reorganisation of compartments post-chemotherapy, are concisely described. Shortening the gene descriptions and highlighting just a handful of key genes (perhaps supplemented by a lengthier table) would enhance the readability of the manuscript.*

We thank the reviewer for this constructive suggestion. We have significantly condensed the text in this section to improve readability, focusing the narrative on the dynamic reorganization of compartments. To maintain the technical details, we have introduced two new tables to the main text (**Table 1, Table 2**) which summarize the cellular niches and their associated genes.

> *12. "These findings, validated across two different therapeutic strategies, suggest that the primary mechanisms of drug tolerance may be drug-independent." – this conclusion may be overstretched, as only two drugs have been tried in mouse and one in human, and mutation accumulation has not been investigated in this context. I would tone down.*

We agree that the conclusion should more accurately reflect the scope of our data. While our CNV inference showed no evidence of genomic diversification in this context, we acknowledge that the investigation was limited to the treatments described. We have toned down the statement to indicate that, within the scope of the drugs examined, tolerance appears to be associated with a convergent transcriptional state rather than specific drug-induced mechanisms.

Included section in the main text:

"*While our analysis does not identify a specific tumour cell subpopulation responsible for recurrence, it provides insight into the heterogeneity and plasticity underlying drug tolerance. Notably, the consistency of these observations across distinct therapeutic regimens suggests that, under these conditions, drug tolerance is associated with a unified transcriptional state rather than being drug specific.*"

> *13. The authors discuss a "permissive TME" that enables drug resistance, which of course makes a lot of sense – however, it is not clear to me from the study what exactly this permissive TME is.*

We thank the reviewer for this comment. We have clarified in the text that the permissive TME is defined by elevated SASP factors, tumour-promoting cell-cell interactions, enriched inflammatory pathways, and the presence of immunosuppressive macrophages. These features undergo extensive remodelling following treatment, paralleling the changes seen in the tumour compartments and supporting their role in enabling drug tolerance.

Included section in the main text:

"This process appears to be supported by a permissive TME, characterised by elevated SASP factors, tumour-promoting cell interactions, inflammatory pathways, immunosuppressive macrophages, which, similarly to the tumour compartments, undergo extensive remodelling following treatment."

> *14. Sometimes, the depictions/arrangement in the figure panels are puzzling:*
> *- Figure 1a/b: confusing order of schematics, and volume is both here and in b. Figure 1b is rather difficult to follow, and the time course not clear. It could benefit from a better structure and more ample caption to describe what is shown.*

We have now completely redesigned **Fig.1a** and **Fig.1b** to streamline the schematics and more clearly convey the experimental time course.

> *- Figure 1i does not really seem to belong to Figure 1, as it is not really linked to any of the previous panels*

Following the reviewer's suggestion, we have removed panel **i** from **Fig.1** and moved it to **Fig.4a**, where it better contextualizes and supports the IMC experiments.

> *- Figure 2a – there are too many colours, arrows and labels. A better clarification of cluster number/colour/label is needed. Why highlight key genes both here and in b?*

We have streamlined this panel to improve clarity and reduce visual complexity. We would like to clarify the distinct roles of these two panels: **Fig.2a** displays the top-weighted genes to highlight the primary drivers of the specific compartments shown, whereas **Fig.2b** presents z-scored weights to emphasize unique markers that distinguish each respective niche.

> *- Figure 2c: it is difficult to match the colours to the niche.*

In **Fig.2c**, we used maximum intensity projection to visualize multiple niches simultaneously, providing a critical overview of the shifts in overall tumour composition. To assist the reader in distinguishing

individual niches, we have provided independent visualizations of each niche in **Supplementary Fig.11**, as now referenced in the main text.

- Figure 2e: it is not obvious at which time point MRD occurs.

We have added indicators to the x-axis labels in **Fig.2e** to explicitly mark the time points corresponding to MRD.

- Figure 2f shows the cell type contribution to the defined tissue compartments – it would make much more sense to define this from the start, i.e. after panel 2b, and then explore differences after treatment (panels d-e, which are the key points you want to leave the readers with).

We appreciate the reviewer's perspective on the sequence of analysis. In our workflow, tissue compartments were first defined based on shared spatial transcriptional programs, independent of cell-type identity. Cell-type contributions were subsequently overlaid to aid in the biological interpretation of these niches, rather than to define them. This approach allowed us to focus on spatially coherent functional states before assessing how treatment reshapes their cellular composition.

- Throughout figure 2 it is difficult to follow when the authors refer to numbered compartments rather than biological niches – it would be beneficial to assign all compartments a biological meaning (e.g. proliferative, EMT, hypoxic, rather than compartment 5, 2, 10) and use those labels throughout the downstream analysis. I believe the authors have even confused themselves, when referring in the text to "proliferating (0)" but in the figure it is compartment 5.

We thank the reviewer for the suggestion to use biological labels, which we have now implemented throughout the manuscript to improve readability. We would like to clarify that compartment 0 and compartment 5 are distinct niches, both characterised by proliferative markers but representing different spatial or functional contexts within the tumour. To resolve any potential confusion arising from this intra-tumoural heterogeneity, we have redesigned **Fig.2a** to clearly associate each compartment number with a specific biological label. Additionally, we have included **Table 2** in the Results section, which provides a definitive annotation for each compartment, including a brief description and representative marker genes.

- Figure 4a: cisplatin treatments are not in order, would make sense to show time course.

We thank the reviewer for the comment. The order used in **Fig.4a** (4 hpt, 1 dpt, 12 dpt, control) follows the temporal progression after cisplatin treatment. We deliberately placed the control (primary tumour) next to the 12 dpt sample to highlight the clearance of Pt over time and the resulting similarity in intracellular Pt content distribution between these two conditions.

Reviewer #3 (Remarks to the Author)

In this manuscript the authors aim to characterize the changes post-chemotherapy in the Brca1-/-;Trp53-/- mouse model using multimodal analysis. Through a combination of single-cell RNA-seq, spatial transcriptomics (ST), and imaging mass cytometry (IMC), the authors delineate the dynamic spatial and temporal characteristics of residual tumor cells and their microenvironment after chemotherapy. They find that an EMT-like tumor cell population that persists post-treatment and appears to be a critical reservoir for recurrence. They show that this EMT program is conserved in human BRCA1-deficient breast cancers and correlates with poor prognosis, making it a potential therapeutic target. Overall the study is comprehensive but largely descriptive. It unclear if there are novel insights gained through the study as EMT has been previously attributed to chemoresistance. However, the level of granularity with which the study has been conducted will provide a good resource to the breast cancer community.

We thank the reviewer for their careful evaluation of our manuscript and for recognising the breadth, technical rigor, and value of the multimodal dataset generated in this study. We agree that EMT has previously been implicated in chemoresistance. However, the central advance of our work lies not in identifying EMT per se, but in resolving its spatiotemporal organisation, persistence, and reversibility during minimal residual disease using longitudinal single-cell, spatial, and proteomic profiling in an immunocompetent BRCA1-deficient model. By integrating these modalities across defined treatment stages, we provide mechanistic insight into how EMT-associated, quiescent tumour niches are maintained during therapy, how they spatially interact with the microenvironment, and how they reconstitute tumour heterogeneity upon recurrence. We further demonstrate conservation of these programmes in human BRCA1-deficient tumours and their association with poor clinical outcome, underscoring both biological relevance and translational potential.

1. In Fig 1c the authors should provide a dot plot of the marker genes used to classify the different populations. In particular how is the LA-EMT cluster defined?

We thank the reviewer for their assessment and for this question. Cell types were annotated based on unsupervised clustering, guided by established breast cancer atlases and markers for basal and luminal alveolar lineages. Additionally, we utilised GFP expression to distinguish tumour cells from non-malignant cells. To provide the requested clarity, we have added a dot plot of the top marker genes used for cluster annotation, including those specifically defining the LA-EMT cluster, in **Supplementary Fig.2**.

2. The authors should use other tools such as stem id, CytoTRACE to understand the Luminal-alveolar to LA-EMT cluster is an undifferentiated cluster. Where is this cluster located in the spatial analysis? In the rest of the paper, when the authors refer to EMT, does it mean it is the LA-EMT cells?

We thank the reviewer for this suggestion. To assess the differentiation state of the tumour clusters, we analysed the scRNA-seq data using **CytoTRACE2**. The analysis predicted LA-EMT cells to be more differentiated relative to the proliferative and basal clusters, which is consistent with our RNA velocity–inferred trajectories (**Fig.R5**).

Regarding the spatial localization, the inferred LA-EMT cells strongly correlate with the EMT compartment (**compartment 2, Fig.2f**), whereas other tumour cell types exhibit significantly lower correlations. Consequently, throughout the manuscript, references to the EMT compartment (niche) specifically incorporate the LA-EMT cell population.

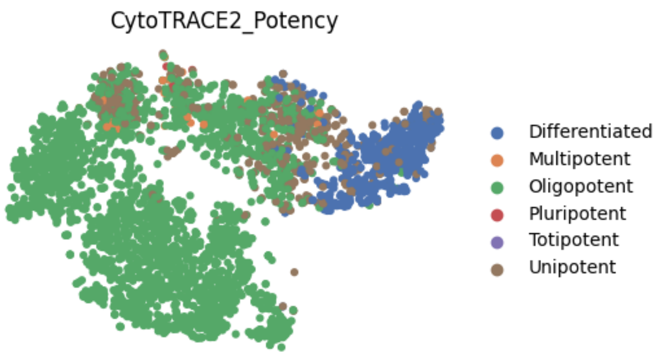

Fig.R5 | CytoTRACE2 classification results on the tumour cell clusters in the scRNA-seq dataset.

> *3. Why are clusters 7 and 8 omitted from the RNA-velocity analysis?*

Due to the low cell abundance in clusters 7 and 8, we omitted them from the RNA-velocity analysis as part of our manual curation process. RNA velocity can be susceptible to high intrinsic noise, which is often amplified in rare cell populations, potentially rendering the directional inference less reliable [3].

> *4. Are the changes seen in the pseudobulk of single cells (Fig 1d) and pseudobulk of spatial (Fig. 1h) overlapping? If so, what percentage? Also, the supplementary table (ST1 and ST2) should be replaced with gene names rather than gene symbols/ids?*

We thank the reviewer for this question. We originally omitted a direct comparison between these platforms because 10x Visium and scRNA-seq have different inherent technical biases, including our use of GFP-based sorting in the scRNA-seq to enrich tumour cells. However, to address this point, we compared the differential expression results and observed that 76% of the significant hits identified in the spatial data were also present in the scRNA-seq dataset (**Fig.R6**).

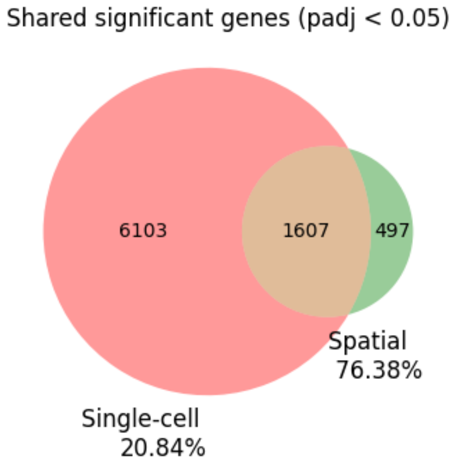

Fig.R6 | Venn diagram showing the overlap of significant genes in the differential gene expression analysis of single-cell and spatial data.

> *5. "In the recurrent stage, however, tumour architecture and cell morphology resembled those of the primary tumours once again." – this sentence needs a figure reference*

We thank the reviewer for drawing our attention to this omission. We have added a reference in the text to **Supplementary Data 1** to support this statement.

6. “Hypoxic tumour cell populations were predominantly located in close proximity to these necrotic cores.” Authors should delineate the hypoxic cores in the figures.

We have added clear labels to the necrotic cores in the corresponding spatial transcriptomics maps and histology scans to facilitate the interpretation of the hypoxic tumour cell populations and their spatial orientation.

7. It is unclear if the EMT-tumor niche (even a small minority) is present before treatment or emerges post-treatment.

We appreciate the opportunity to clarify this. Our data show that the EMT niche is present prior to treatment and does not emerge solely as a result of chemotherapy. The temporal dynamics of this niche are detailed in the "**Chemotherapy-induced changes in the transcriptional tissue landscape of MRD**" subsection of the **Results**. This is further illustrated in **Fig.2d** and **Fig.2e**, which provide the spatiotemporal distribution and quantification of the EMT niche across all stages, including primary tumours.

Included section in the discussion for clarification:

“Importantly, the EMT-associated tumour niche was already detectable at low abundance in treatment-naïve primary tumours and persisted with largely unchanged frequency throughout chemotherapy, indicating selection of a pre-existing drug-tolerant state rather than de novo induction by treatment.”

8. The paper in general is very dense to read and it is hard to easily evaluate the supplementary spatial transcriptomic data. Maybe the authors can come up with more summarized figures

We thank the reviewer for this constructive feedback. We have significantly streamlined the text to improve readability and redesigned several figure panels to better guide the reader through our multi-modal findings. Furthermore, we have introduced **Table 1** and **Table 2**, which provide consolidated summaries of differentially expressed genes and cellular niche annotations. We believe these modifications substantially enhance the presentation and accessibility of our results.

Reviewer #4, Expertise: , breast cancer mets, mouse models (Remarks to the Author):

In the manuscript entitled “Spatiotemporal organisation of residual disease in mouse and human BRCA1-deficient mammary tumours and breast cancer,” Turos et al. combine single-cell RNA sequencing, spatial transcriptomics, and imaging mass cytometry of BRCA1;p53-deficient mouse mammary tumors to characterize the dynamics of specific tumor cell subpopulations and their surrounding microenvironments during minimal residual disease (MRD) and recurrence. Overall, this study integrates single-cell and spatial omics approaches to study the BRCA1;p53-deficient mouse mammary tumors and their response to standard therapies. The authors also validated some gene signatures in patient samples. While the conclusions the authors are able to draw from the omic data are largely based on and consistent with published literatures, the study is largely correlative and descriptive without performing functional validations of any cell types or signaling pathways for their roles in tumor growth and/or responses to therapies. Therefore, this study provides an omic resource of this BRCA1;p53-deficient mouse mammary tumor model, but not a research article.

We thank the reviewer for their careful evaluation of our submission and for highlighting the need to move beyond correlative multi-omic observations towards functional validation. We agree that, in the initial version of the manuscript, the conclusions were primarily based on descriptive and correlative

analyses of tumour cell states and microenvironmental niches. In direct response to this critique, we have substantially revised the manuscript and added new functional experiments that test the biological relevance of the EMT-associated MRD programme identified in our multi-omic analyses. Specifically, we now include *ex vivo* tumoroid assays and pharmacological perturbation experiments targeting Galectin-3, a key component of the EMT niche programme, demonstrating a functional role for this pathway in tumour cell survival and drug tolerance. These new data provide experimental validation that the EMT-associated niche is not merely correlative but contributes causally to therapy response and residual disease persistence. We believe that these additions significantly strengthen the mechanistic insight of the study and increase the work beyond being descriptive.

> *Reviewer #4 (Remarks on code availability):*
> *N/A*
> *Specific points:*
> *1. The study is descriptive. The authors do not provide any functional validation of the relevance of the identified tumor or stromal (e.g., macrophage) populations in MRD and recurrence. The manuscript would be more compelling if the authors attempted to functionally assess the role of some of these populations.*

We thank the reviewer for this insightful comment. We agree that functional validation significantly strengthens the implications of our spatiotemporal mapping. In response, we have performed new functional experiments using a residual tumoroid model derived from primary cells of the KB1P mouse model.

As our primary focus was to characterize the EMT niche, we identified candidate therapeutic targets among proteins significantly enriched in the EMT expression program. We selected the Galectin-3 inhibitor GB1107 for functional assessment. Using a drug synergy assay, we observed a potent synergistic effect between Galectin-3 inhibition and chemotherapy in the residual tumoroid model (**Fig.6**). These results provide functional evidence that targeting the EMT-associated niche can sensitize residual disease to treatment, elevating the study from a descriptive resource to a research article with a functionally validated therapeutic target.

> *2. Suppl. Figure 2 is intended to support the data presented in Fig. 1F. The authors report differences in the presence of EMT cells and immune cell infiltrates (such as macrophages) based on the spatial transcriptomics data. However, Suppl. Fig. 2 includes only a single representative image, which limits interpretation. In this figure, the authors display stromal populations only in treatment-naïve tumor, but do not show how these populations appear in the residual tumor (except for macrophages, which are shown in panel D). On the opposite, they present tumor populations in residual tumors, but not in treatment-naïve primary tumors.*

We thank the reviewer for this observation. To provide a more comprehensive overview beyond representative images, we have added a new supplementary data file **(Supplementary Data 1)** containing the inferred abundances for 23 distinct cell types across all spatial transcriptomics samples. This allows for a quantitative comparison of both stromal and tumour populations across the entire transition from treatment-naïve to residual and recurrent states.

> *3. Suppl. Figure 3 is difficult to interpret due to disorganized sample nomenclature and treatment time points. It would be helpful if the authors provided a quantification of the percentage of the different areas represented in the figure. Additionally, are there observable differences in necrotic areas between treatment-naïve and residual tumors?*

Necrotic areas were assessed based on histopathological annotations, as provided in **Supplementary Fig.4**. We acknowledge the reviewer's request for quantification. However, due to the

inherent limitations of 2D sectioning, we believe that volumetric quantification would be statistically unreliable. Nevertheless, our qualitative assessment across the dataset revealed substantial variability in necrotic regions throughout tumour progression, with no consistent bias observed toward any specific treatment time point.

4. Graphs in Fig. 1F, Suppl. 4C and D, and Fig. 2E lack statistical validation.

We thank the reviewer for pointing out these omissions. We have now added statistical validation to the aforementioned panels. Where appropriate, we performed a one-way ANOVA followed by Tukey's HSD post-hoc test for multiple comparisons, with *p*-values now indicated in the figures and legends.

5. In figure 2C, the authors investigated tumor evolution at four time points: pre-treatment, MRD at 7 and 12 days post-treatment, and recurrent tumor, in both TAC- and cisplatin-treated samples. In the manuscript, the authors state that MIP visualizations revealed major shifts in niche composition: non-treated tumors were dominated by the proliferative niche (5), fibrotic stroma (4), and hypoxic regions (10), while EMT niches were scattered. In MRD, compartments 11 and 12 became predominant, indicating immune infiltration and macrophage activity. Could the authors highlight these changes more clearly in Figure 2C, for example by labelling the relevant compartments, to aid interpretation?

We thank the reviewer for this suggestion. We have redesigned **Fig.2a** to better guide the interpretation of the MIP visualizations by highlighting the relevant cellular niches. While the main figure (**Fig.2c**) provides a global overview of niche shifts, in **Fig.2d** we have focused specifically on the tumour niches. Furthermore, we direct the reader to **Supplementary Fig.11-12**, which displays individual compartments on spatial maps for more detailed information of temporal dynamics across treatment and recurrence.

Reviewer #5, Expertise: IMC (Remarks to the Author):

The topic presented in this manuscript is both relevant and interesting, using a variety of imaging techniques as well as spatial transcriptomics (ST) and imaging mass cytometry (IMC).

The introduction provides an adequate but not excessive background on Triple Negative Breast Cancer and the reasoning behind the analyses. Special emphasis is given to the topic of Minimal Resistant Disease (MRD), a key aspect of the cancer biology subject of the paper.

The results section provides a thorough description of the exhaustive analyses conducted for this manuscript. The reviewers provide myriad figures which attempts to capture the experimental results in an encyclopedic fashion. Occasionally, we have found that it is difficult to see the actual described results within the figures. Specific examples are detailed below.

The discussion section is relatively short and lacks many strong statements regarding any patterns observed in the very large number of analyses performed within the manuscript. The most interesting trends observed are that the EMT niche appears to play a central role in tumor survival through MRD and the reinforcement of the observation that drug uptake in tumor cells occurs independent of the cellular population. Although it would be beneficial if more trends could be extracted from the large amount of data, this is not strictly necessary. We would have liked to see more of an explanation as to why certain isotopes of platinum may have proved better for analyses than other isotopes.

The methods section is thorough and provides adequate detail as to how independent investigators might replicate the study. The investigators have provided adequate detail for how biological samples were obtained, how spatial transcriptomics was conducted, and how IMC analyses were conducted. The authors have also detailed their computational methods for cell phenotyping, transcriptional analyses, cellular classification, IMC analyses, and cellular niche assignments with code that has been uploaded for common use.

Overall, the manuscript provides more description of the biology than explanation of the biology, but we believe that the thoroughness of the analyses and the important subject of experimentation merits inclusion within the journal. The quantity of data and analyses in this carefully written paper are immense and powerful in their own right.

We thank the reviewer for their thoughtful and constructive evaluation of our manuscript and for recognising both the relevance of the biological question and the breadth of the multimodal analyses performed. We appreciate the reviewer's careful assessment of the Introduction, Methods, and the technical rigor of the spatial transcriptomics and imaging mass cytometry workflows, as well as their acknowledgement of the value of making the data and code publicly available. We agree that the manuscript contains a large volume of data and that, in places, the key biological messages could be more clearly highlighted. In the revised version, we have therefore focused on improving figure readability, clarifying the presentation of results, and strengthening the Discussion to more explicitly articulate the major biological patterns emerging from the analyses, particularly the central role of the EMT-associated niche in MRD and the observation that drug uptake occurs largely independently of tumour cell state. We have also expanded the discussion of the platinum isotope selection and interpretation of the IMC data to address the reviewer's comments.

Proposed edits:
• Please elaborate further on possible causes of 198Pt saturation in IMC detection. Do control samples demonstrate an unusual 198Pt signal? Could the cisplatin sample be tested by Mass Spec to find any unexpected distribution of Pt isotopes?

We thank the reviewer for this insightful question regarding the platinum signal quantification. In our quality control analysis, we observed that while ^{194}Pt, ^{195}Pt, and ^{196}Pt showed extremely high spatial co-localization (Pearson's $r > 0.95$), the ^{198}Pt signal exhibited a poor correlation with the other isotopes ($r \approx 0.65$) and lacked the expected biological distribution patterns.

We attribute this anomalous ^{198}Pt intensity to instrumental background and ion interference rather than a deviation in the cisplatin isotopic distribution. We confirmed that the ^{198}Pt signal was not specific to treated samples but appeared as an inconsistent, non-spatial background across various regions of interest, including controls. Given that ^{198}Pt represents only ~7.36% of the natural abundance and its signal-to-noise ratio in our system was insufficient to provide reliable biological data, we opted to exclude it to ensure the highest stringency for our spatial quantification. The high correlation between the other three primary isotopes (comprising ~91.2% of natural abundance) provides sufficient confidence in the localization of the platinum drug.

Included section in the results paragraph "Multimodal characterisation of cellular cisplatin uptake in mammary tumours" for clarification:

"First, we quantified intracellular Pt signal intensity to infer cisplatin uptake within tumour tissue. As Pt content was directly measured by IMC detectors without signal amplification, we first evaluated the isotopic distribution and kinetics of Pt within the tissue. The measured abundances of ^{194}Pt, ^{195}Pt, and ^{196}Pt closely matched the expected isotopic distribution (^{190}Pt: 0.01%, ^{192}Pt: 0.78%, ^{194}Pt: 32.9%, ^{195}Pt: 33.8%, ^{196}Pt: 25.2%, ^{198}Pt: 7.36%)[83], and exhibited the anticipated clearance kinetics following

*treatment. In contrast, ^{198}Pt showed higher intensity than expected and lacked spatial correlation with the other Pt isotopes, indicating nonspecific signal, leading us to exclude it from further analysis (**Supplementary Fig.23**). Signals for ^{190}Pt, and ^{191}Pt were indistinguishable from untreated controls, suggesting that isotope levels below the detection limit, and were similarly omitted from the analysis.*"

> *• Figure 4j was not referenced in the text. Was this intentional?*

We thank the reviewer for this observation. The reference was inadvertently omitted from the main text. As part of our restructuring to improve the clarity and focus of the manuscript, we have moved **Fig.4j** to **Supplementary Fig.27**. The text has been updated to correctly reference this panel within the Supplementary Materials.

> *• Supplementary figure 21c was referenced in the text of the paper but does not exist in the supplementary figures*

We thank the reviewer for identifying this error. We have corrected the reference in the text to point to the correct panel, **Supplementary Fig.21b**, and removed the erroneous reference to **21c**.

[1] Zhou L, DeMarco K D, Murphy K C, Wu Z, Li J, Johnson C, Liu B, Giwa H K, Ma B, Bhalerao N, Li J, Jiang Z, Bai S, Parikh C N, Ye T, Simin K, Zhu L J, Pitarresi J R, Wu H and Ruscetti M 2025 P21-positive senescent stromal cells promote prostate cancer immune suppression and progression that can be reversed by senolytic therapy *Cancer Discov.*
[2] Pereira B, Chin S-F, Rueda O M, Vollan H-K M, Provenzano E, Bardwell H A, Pugh M, Jones L, Russell R, Sammut S-J, Tsui D W Y, Liu B, Dawson S-J, Abraham J, Northen H, Peden J F, Mukherjee A, Turashvili G, Green A R, McKinney S, Oloumi A, Shah S, Rosenfeld N, Murphy L, Bentley D R, Ellis I O, Purushotham A, Pinder S E, Børresen-Dale A-L, Earl H M, Pharoah P D, Ross M T, Aparicio S and Caldas C 2016 The somatic mutation profiles of 2,433 breast cancers refine their genomic and transcriptomic landscapes *Nat. Commun.* **7** 11479
[3] Qiao C and Huang Y 2021 Representation learning of RNA velocity reveals robust cell transitions *Proc. Natl. Acad. Sci.* **118** e2105859118
